# *Bacillus cereus* Toxin Repertoire: Diversity of (Iso)cereulide(s)

**DOI:** 10.3390/molecules27030872

**Published:** 2022-01-27

**Authors:** Veronika Walser, Markus Kranzler, Corinna Dawid, Monika Ehling-Schulz, Timo D. Stark, Thomas F. Hofmann

**Affiliations:** 1 Food Chemistry and Molecular Sensory Science, Department of Molecular Life Sciences, School of Life Sciences, Technical University of Munich, Lise-Meitner-Str. 34, 85354 Freising, Germany; veronika.walser@tum.de (V.W.); corinna.dawid@tum.de (C.D.); thomas.hofmann@tum.de (T.F.H.); 2 Institute of Microbiology, Department of Pathobiology, University of Veterinary Medicine Vienna, Veterinärplatz 1, 1210 Vienna, Austria; markus.kranzler@vetmeduni.ac.at (M.K.); Monika.Ehling-Schulz@vetmeduni.ac.at (M.E.-S.)

**Keywords:** *Bacillus cereus*, cereulide, isocereulides, structure elucidation, UPLC-MS, NMR spectroscopy, MS^n^

## Abstract

The emetic *Bacillus cereus* toxin cereulide (**1**) poses a significant safety risk in the food industry, causing emesis and nausea after consumption of contaminated foods. Analogously to cereulide, the structures of various isocereulides, namely, isocereulides A–G, have been recently reported and could also be identified in *B. cereus*-contaminated food samples. The HPLC fractionation of *B. cereus* extracts allows us to isolate additional isocereulides. By applying MS^n^ sequencing, post-hydrolytic dipeptide, amino acid and α-hydroxy acid analyses using UPLC-ESI-TOF-MS to purify the analytes, seven new isocereulides H–N (**2**–**8**) could be elucidated in their chemical structures. The structure elucidation was supported by one-dimensional and two-dimensional NMR spectra of the isocereulides H (**2**), K (**5**), L and N (**6** + **8**) and M (**7**). The toxicity of **2**–**8** was investigated in a HEp-2 cell assay to determine their respective 50% effective concentration (EC_50_). Thus, **2**–**8** exhibited EC_50_ values ranging from a 0.4- to 1.4-fold value compared to cereulide (**1**). Missing structure-activity correlations indicate the necessity to determine the toxic potential of all naturally present isocereulides as single compounds to be able to perform a thorough toxicity evaluation of *B. cereus*-contaminated foods in the future.

## 1. Introduction

**Figure 1:**
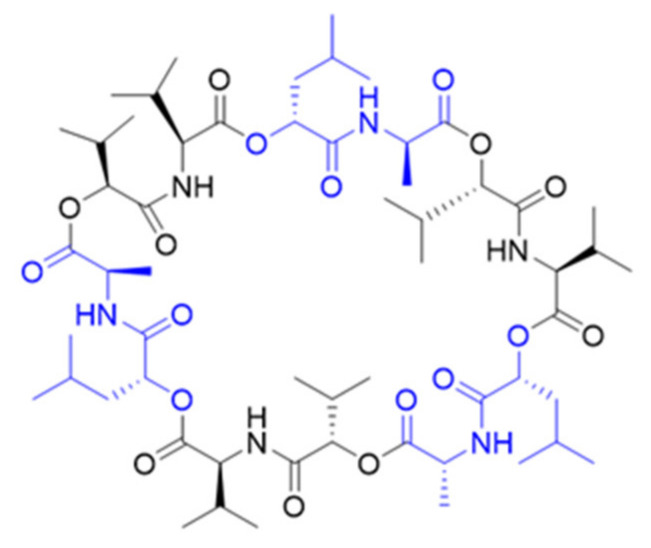
Chemical structure of cereulide with d-*O*-Leu-d-Ala in blue and l-*O*-Val-l-Val in black.

The ubiquitous, endospore-forming, facultative anaerobe bacterium *Bacillus cereus* is commonly categorized as a food-borne pathogen, causing gastrointestinal diseases and emesis arising from the production of various enterotoxins and the emetic toxin cereulide (**1**) [1]. Cereulide (**1**) is composed of 12 alternatingly placed α-amino and α-hydroxy acids thought to form the three-time circularly repeating tetradepsipeptide unit d-*O*-Leu-d-Ala-l-*O*-Val-l-Val (Figure 1), leading to its characteristic dodecadepsipeptide structure in a rectangular cylindrical shape [2,3,4,5]. Because of its unique chemical structure, **1** was found to be inert to a wide range of temperatures, pH values and enzymes, making it a considerable safety risk to the food industry [6,7,8,9].

In recent years, screenings of *B. cereus* extracts have revealed a wide range of cereulide structure homologs, of which the seven isocereulides A–G could be identified and elucidated in their structure [10,11,12]. In addition, the chemical structure of isocereulide A has been recently revised [13]. Moreover, a cereulide isoform differing by +14 Da from the mass-to-charge ratio of **1**, referred to as homocereulide, was synthesized as cyclo (l-*O*-*allo*-Ile-d-Val-d-*O*-Leu-d-Ala [l-*O*-Val-l-Val-d-*O*-Leu-d-Ala]_2_), even though no reference to the already known isocereulides was made [14].

The complex structure of naturally occurring **1** is biosynthetically assembled through nonribosomal peptide synthetases (NRPS), with its CesNRPS genes, cesA and cesB, located on a pXO1-related virulence megaplasmid and organized as an operon [15,16,17,18]. Other than the aforementioned cereulide synthetase genes cesA and cesB, the ces gene locus involves a phosphopanthetein transferase for activating NRPS machinery (cesP), a type II thioesterase (cesT) which has a proofreading function, and an ABC transporter (cesC/D), which is involved not only in cereulide export but also directly in cereulide biosynthesis [19,20,21]. By coupling in situ reduced d-Leu moieties with d-Ala, cesA generates a peptidyl carrier protein (PCP)-coupled d-*O*-Leu-d-Ala didepsipeptide intermediate, analogous to cesB assembling the corresponding l-*O*-Val-l-Val intermediate [22,23]. Subsequently, cereulide (**1**) biosynthesis was described to occur via a generated l-*O*-Val-l-Val-d-*O*-Leu-d-Ala-PCP-coupled intermediate, whereas the assembly of isocereulides F and G was proposed as occurring via an unexpected addition of a TE-bound didepsipeptide [24]. Recent model studies on the TE-dependent trimerization and macrocyclization of the tetradepsipeptide unit l-*O*-Val-l-Val-d-*O*-Leu-d-Ala by cesT found cereulide (**1**) synthesis in line with the biosynthesis pathway of the structurally similar toxin valinomycin [5,25].

Similar to the antibiotic valinomycin, cereulide unfolds its toxicity potential due to a high affinity for complex formation with alkali metal ions (Li^+^, Na^+^ and K^+^) and ammonium ions, resulting in its biological function as a strong K^+^ ionophore and as a K^+^ transporter, resulting in cell membrane depolarization [26,27,28]. Using a porcine model, it was recently shown that **1** can cross the blood–brain barrier, possibly causing a disturbance of the potassium content of the cerebrospinal fluid, which might partially explain the cerebral effects reported from human intoxication cases [29]. In lower concentrations, **1** was found to provoke vomiting in an animal model via stimulation of the vagus afferent by binding to 5–HT_3_ receptors located in the duodenum [30]. Additional effects observed for **1** are cellular damage and inhibition of natural killer cells of the immune system [31,32]. Cytotoxicity screening of pre-fractionated *B. cereus* cell extracts was performed using HEp-2 cells in parallel to structure determination of isocereulides A–G, showing increasing toxicity with growing analyte hydrophobicity [12]. Furthermore, synthetically produced homocereulide showed an approx. 2.8-fold increased effect in vacuolation assays [14].

Cereulide (**1**) and isocereulides formed in foods by food-borne pathogen *B. cereus* show a significant safety risk to the food industry [33], resulting in food wastage, thus economic and environmental damage [8]. The present study provides a more comprehensive insight into cereulide chemodiversity by unraveling the complex composition of the broad variation in isocereulide heterogeneity. Conducting UPLC-TOF-MS experiments, ion-trap MS^n^ sequencing, post-hydrolytic dipeptide, enantioselective amino acid and α-hydroxy acid analyses, and 1D- and 2D-NMR experiments, the structures of seven additional isocereulides could be assigned after isolation from *B. cereus* cell extracts. Through a cytotoxicity screening on HEp-2 cells, the bioactivity of **1** and the newly identified isocereulides H–N (**2**–**8**) was investigated to estimate their contribution to the overall toxicity of *B. cereus*-contaminated food samples.

## 2. Results

**Mass spectrometric characterization and isolation of cereulide (1) and isocereulides H–N (2–8).** Next to cereulide, 18 additional isocereulides were reported to be mass-spectrometrically detected in the range between 1147.6366 and 1205.6785 Da, exhibiting a constant ratio of ~100:40:25 for the pseudomolecular ions [M + Na]^+^, [M + K]^+^ and [M + NH_4_]^+^ [12]. These findings relative to the isocereulides correlate with the ability to form high-affinity complexes with alkali metals and ammonium ions reported for cereulide [4,26]. Guided by literature protocol [12], further cereulide-like structures, other than the recently reported isocereulides A–G, could be located. Next to cereulide (**1**; *m*/*z* 1175.6679), seven new isocereulides, namely, isocereulide H (**2**; *m*/*z* 1161.6522), isocereulide I (**3**; *m*/*z* 1161.6522), isocereulide J (**4**; *m*/*z* 1147.6366), isocereulide K (**5**; *m*/*z* 1189.6835), isocereulide L (**6**; *m*/*z* 1191.6628), isocereulide M (**7**; *m*/*z* 1161.6522) and isocereulide N (**8**; *m*/*z* 1191.6628) could be isolated, allowing structure elucidation (Table 1). Further information about the purification of **1**–**8** is provided in the Supplementary Materials (Figure S2).

**Figure 2:**
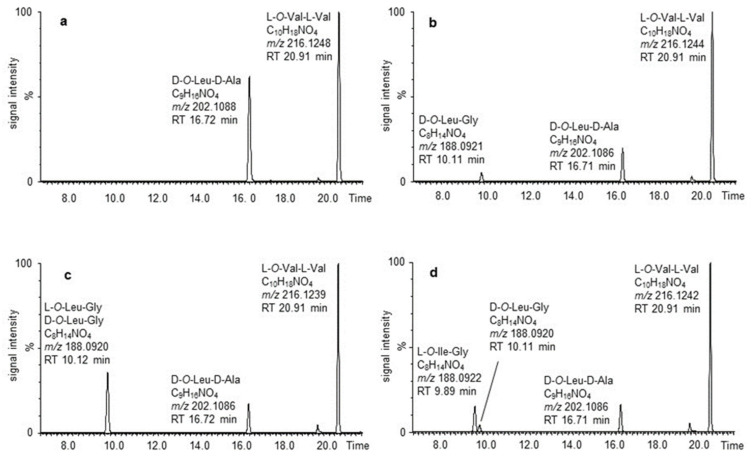
UPLC-ESI^–^-TOF-MS chromatograms of alkaline hydrolysis of (**a**) cereulide (**1**), (**b**) isocereulide H (**2**), (**c**) isocereulide H (**2**) spiked with l-*O*-Leu-Gly and (**d**) isocereulide H (**2**) spiked with l-*O*-Ile-Gly.

**Structure elucidation of isocereulides H–N (2–8).** Following the recently reported structures for isocereulides A–G [12], the masses of [M + Na]^+^ pseudomolecular ions of the newly detected isocereulides H–N (**2**–**8**) were found to vary by 14–28 Da from the [M + Na]^+^ adduct of cereulide (**1**; *m*/*z* 1175.6679) (Table 1).Exemplary, **2** was detected as having an *m*/*z* of 1161.6522 ([M + Na]^+^), resulting in the elemental composition of C_56_H_94_N_6_O_18_Na. The observed mass shift of −14 Da indicated an overall loss of one methylene group compared to **1′**s structure (3 × Ala, 3 × Val, 3 × *O*-Leu and 3 × *O*-Val) and led toward the possible exemplary exchange of one Ala by one Gly, one Val by one 2-Abu moiety, one *O*-Leu by one *O*-Val, or one *O*-Val by one 2-hydroxybutanoic acid unit. Via UPLC-ESI^–^-TOF-MS analysis, after alkaline hydrolysis, the released dipeptides of dodecadepsipeptides **1**–**8** were investigated to gain a first insight into their amino acid and α-hydroxy acid composition. Analyzing the alkaline hydrolysate of **2**, d-*O*-Leu-d-Ala (*m*/*z* 202.1086; [M − H]^−^), L-*O*-Val-L-Val (*m*/*z* 216.1244; [M − H]^−^) and one other dipeptide (*m*/*z* 188.0921; [M − H]^−^; C_8_H_14_NO_4_) were observed (Figure 2b) in a ratio of 2:3:1, implying the substitution of one d-*O*–Leu-d-Ala moiety compared with cereulide (**1**; 3 × d-*O*-Leu-d-Ala and 3 × l-*O*-Val-l-Val; Figure 2a).

UPLC-ESI^–^-TOF-MS^e^ analysis of the candidate dipeptide released from **2** (*m*/*z* 188.0921; [M − H]^–^; C_8_H_14_NO_4_) revealed an MS^e^ fragmentation pattern with fragment ions at an *m/z* of 144.1020 ([M − H]^–^; C_7_H_14_NO_2_) and an *m*/*z* of 74.0243 ([M − H]^–^; C_2_H_4_NO_2_), respectively. These observed *m*/*z* exhibited the expected pattern for *O*-Leu-Gly, providing the mother mass and the fragments formed after decarboxylation and cleavage of the peptide bond (Supplementary Materials, Figure S4.1c). This observation indicated the exchange of one Ala for one Gly unit but also gave room to the possibility of one *O*-Leu being exchanged by the isobaric *O*-Ile.

The eligible reference dipeptides for all isocereulide samples were synthesized through solid-phase peptide synthesis (Supplementary Materials, Section B) and compared to the dissenting dipeptide present in the respective isocereulide by applying co-chromatography. Therefore, the alkaline hydrolysate of **2** was spiked with l-*O*-Leu-Gly (Figure 2c) and l-*O*-Ile-Gly (Figure 2d), respectively. Only l-*O*-Leu-Gly matched the retention time and increased the signal intensity of the candidate dipeptide, whereas l-*O*-Ile-Gly revealed a new analyte signal. These findings confirm the inclusion of the leucine-containing dipeptide in isocereulide H (**2**). The collected data on the released dipeptides of isocereulides H–N (**2**–**8**) are summarized in Table 2.

A possible co-elution of enantiomeric l-*O*-Leu-Gly and d-*O*-Leu-Gly under given conditions demanded a subsequent stereospecific free amino acid and *α*-hydroxy acid UPLC-TOF-ESI^–^-MS analysis after acidic hydrolysis of alkaline hydrolysates. All hydrolyzed samples, including cereulide (**1**) and all respective enantiopure amino acids and *α*-hydroxy acids as references, were derivatized for specifying their stereochemistry. The obtained data from the analysis of isoindole-amino acid derivatives and MTPA-*α*-hydroxy acid esters from **2** (Supplementary Materials, Table S1) indicated the presence of the amino acids d-Ala, l-Val and glycine and the α-hydroxy acids l-*O*-Val and d-*O*-Leu, whereas l-Ala, d-Val, d-*O*-Val and l-*O*-Leu were lacking.

Considering all the data for **2**, the dipeptides l-*O*-Val-l-Val, d-*O*-Leu-d-Ala and d-*O*-Leu-Gly were identified in a ratio of 3:2:1. Accordingly, the structural composition of **3**–**8** was determined. The exact dipeptide sequences of **2**–**8** were investigated through sequential MS^n^ fragmentation. The favored ester cleavage of the depsipeptide structure elicited the formation of a maximum of six theoretically possible isobaric open-chain pseudomolecular ions. In consequence, one was selected as the precursor ion for further fragmentation along its predicted dipeptide pattern (Supplementary Materials, Section F).

**Figure 3:**
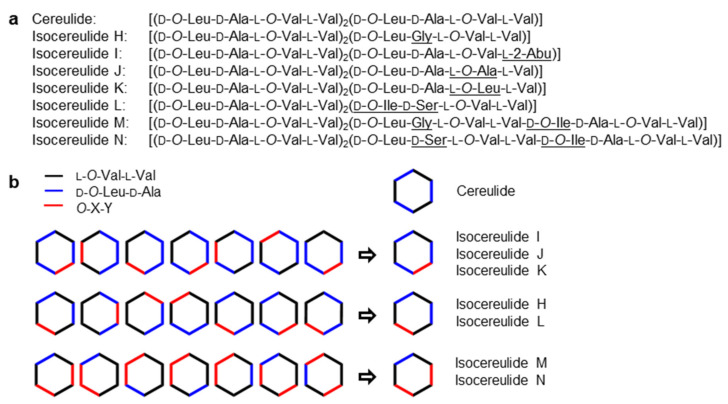
(**a**) Amino and α-hydroxy acid sequences of cereulide (**1**) and isocereulides H–N (**2**–**8**) with compositional changes underlined; (**b**) theoretically possible constitutional isomers (left) and constitution at hand determined by MS^n^ sequencing (right).

A comparison of the obtained [M + K]^+^ pseudomolecular ions of **1** and **2** showed high conformity in their respective MS, MS^2^ and MS^3^ scans, where a successive loss of d-*O*-Leu-d-Ala and l-*O*-Val-l-Val could be observed (**1**: *m*/*z* 1191.7 → 1006.6 → 807.5. **2**: *m*/*z* 1177.7 → 992.5 → 793.5). Fragmentation patterns differed only in the following MS^4^ scan, exhibiting key fragments (**1**: *m*/*z* 807.5 → 736.4/622.3. **2**: *m*/*z* 793.5 → 734.4/622.3), thereby revealing the elimination of one Gly followed by one *O*-Leu moiety for **2**. The accordance in the fragment ions of the respective MS^5^ spectrum (**1**, **2**: *m*/*z* 622.3 → 523.1/423.1) confirmed the preservation of the basic amino acid and α-hydroxy acid sequence of **1**. Consequently, the structure of the cyclic isocereulide H (**2**) was identified as [(d-*O*-Leu-d-Ala-l-*O*-Val-l-Val)_2_(d-*O*-Leu-Gly-l-*O*-Val-l-Val)] (Figure 3a) with its theoretically possible and determined dipeptide sequences shown in Figure 3b.

This way, the structures of isocereulides I–N (**3**–**8**) were elucidated (Figure 3a,b). According to the literature [12], a change in the stereochemistry of the replaced amino acid or *α*-hydroxy acid was not observed for any isocereulide. The chemodiversity within the structures of **2** and **4** was well in accordance with that in the literature [12], where, for unidentified isocereulides, an exchange of one Ala for one Gly and one *O*-Val/Val for one *O*-Ala/Ala, respectively, was predicted. Therefore, isocereulide J (**4**) was identified as [(d-*O*-Leu-d-Ala-l-*O*-Val-l-Val)_2_(d-*O*-Leu-d-Ala-l-*O*-Ala-l-Val)]. For isocereulide I (**3**), the differing dipeptide was identified as l-*O*-Val-l-2-Abu, resulting in the chemical structure [(d-*O*-Leu-d-Ala-l-*O*-Val-l-Val)_2_(d-*O*-Leu-d-Ala-l-*O*-Val-l-2-Abu)]. Interestingly, the exchange of one amino acid by one non-proteinogenic amino acid has not been reported in a naturally occurring isocereulide so far. The structure of isocereulide K (**5**) was identified as [(d-*O*-Leu-d-Ala-l-*O*-Val-l-Val)_2_(d-*O*-Leu-d-Ala-l-*O*-Leu-l-Val)], matching the originally reported chemical structure of isocereulide A [12], which has been recently updated [13]. Isocereulides L and N (**6** + **8**) were isolated as a mixture (approx. 42/58) and their structures were elucidated in parallel and compared with the isobaric isocereulide C [12]. While isocereulide C comprised a d-*O*-Leu-d-Ser moiety, for the mixture of the isocereulides L and N (**6** + **8**), one d-*O*-Ile-d-Ser, one d-*O*-Leu-d-Ser and one d-*O*-Ile-d-Ala unit could be identified. For all three isocereulides to differ in their chemical structure, **6** could only include the d-*O*-Ile-d-Ser dipeptide, whereas the other two dipeptides had to be located within the structure of **8**. Thus, after this combinatory composition, their structures were proposed as [(d-*O*-Leu-d-Ala-l-*O*-Val-l-Val)_2_(d-*O*-Ile-d-Ser-l-*O*-Val-l-Val)] for **6** and d-*O*-Leu-d-Ala-l-*O*-Val-l-Val-d-*O*-Leu-d-Ser-l-*O*-Val-l-Val-d-*O*-Ile-d-Ala-l-*O*-Val-l-Val for **8**. Due to the isobaric properties of d-*O*-Leu and d-*O*-Ile, the position of the d-*O*-Ile-d-Ala moiety in **8** could not be set unequivocally; therefore, it is interchangeable with the present d-*O*-Leu-d-Ala moiety. The same applies to isocereulide M (**7**), where the structure was proposed as d-*O*-Leu-d-Ala-l-*O*-Val-l-Val-d-*O*-Leu-Gly-l-*O*-Val-l-Val-d-*O*-Ile-d-Ala-l-*O*-Val-l-Val. The data on alkaline hydrolysis, acidic hydrolysis and MS^n^ sequencing of all analyzed isocereulides are summarized in the Supplementary Materials.

Using traveling wave IMS-qTOF MS, rotationally averaged collision cross section (CCS) values for the K^+^-adducts of **1** and isocereulides A–G and H–N (**2**–**8**) were determined from pre-fractionated mixtures or their purified reference substances, with all values ranging from 347.4 to 361.1 Å^2^ (Table 1). Due to the cyclic arrangement of the described isocereulides and their close molecular weight (Δ~1%), a high similarity in their three-dimensional structure is assumed, leading to nearly identical CCS values. Keeping in mind a general deviation in the CCS of approx. 2% [34], a differentiation of **1** and **2**–**8**, especially in their complex natural constitution, could not be observed on the basis of only their CCS.

**Determination of EC_50_ of cereulide (1) and isocereulides H–N (2–8) via HEp-2 cell assay**. The testing principle of the HEp-2 cell assay is based on the uncoupling of ATP synthesis, effectuated by supplemented ionophores, resulting in a facilitated ion influx into the mitochondria, thus disrupting the membrane potential. As a result, the 50% effective concentration (EC_50_) gives the amount of toxin required to inactivate half of the viable cells. The obtained EC_50_ for cereulide (**1**) and the structurally similar reference toxin valinomycin exhibited a discrepancy in respect to each other, displaying that **1** spawned a 13.7-times higher toxic effect than valinomycin at an equally applied concentration (data not shown). This result agrees well with literature findings, where **1** is described to hold an approx. 15-fold increased toxicity compared to valinomycin [35]. The results of an HEp-2 cell screening of pre-fractionated *B. cereus* cell extracts showed an increase in the effect on the cells with the growth of analyte hydrophobicity, resulting in a coherence of the toxin’s effect on the cells with the grade of its ionospheric properties [12].

**Figure 4:**
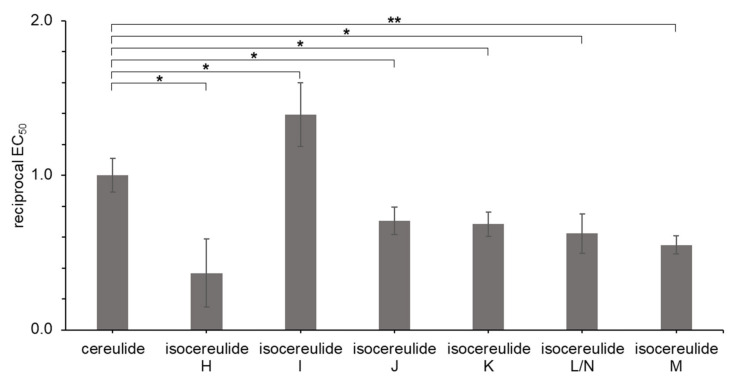
Cytotoxic activity of purified cereulide (**1**) and isocereulides H–N (**2**–**8**), given in their reciprocal EC_50_ with cereulide set to a value of **1**. The ethanolic solutions (1%) of the toxins were tested on 1 × 10^5^ HEp-2 cells. Statistical difference between samples calculated based on a two-sided t-test with * being significant at *α* = 0.05 and ** being highly significant at *α* = 0.01.

While the naturally most abundant cereulide (**1**) showed an absolute EC_50_ of 2.44 ng/mL, the isocereulides H–N (**2**–**8**) exhibited significantly deviating concentrations between 1.75 (I, **3**) and 6.62 ng/mL (H, **2**; exact sample composition and EC_50_ values in Supplementary Materials, Table S2), indicating a toxicity range from 0.4- to 1.4-fold of **1′**s toxicity (Figure 4). Especially, **2** stood out with a 63% decreased toxicity effect on HEp-2 cells. Compound **3**, despite its slightly more hdrophilic properties, exhibited a 1.4-fold higher toxic effect than **1** and constituted an exception to that hypothesis. Similarly, the EC_50_ for **5**, which is more hydrophobic than **1**, deviated from the hypothesis—regarding the analyte hydrophobicity—with a cytotoxicity of 69%, compared to **1**.

## 3. Discussion

The production of variants of non-ribosomally produced peptide toxins, known from a variety of cyanobacteria, is generally based on the molecular diversity of the underlying structural genes [36]. However, isocereulides A–G are reported to be produced simultaneously by one single non-ribosomal synthetase, Ces-NRPS (encoded on a mega plasmid), predicted by the full sequencing of the genome of the *B. cereus* reference strain F4810/72 and excluding the presence of paralogous *ces* genes [12,16]. UPLC-TOF-MS studies on the bio-synthetic production of **1** via structure elucidation of depsipeptide intermediates proposed that the general cereulide formation occurs in the form of tetradepsipeptides. d-*O*-Leu-d-Ala and l-*O*-Val-l-Val are separately pre-formed by *cesA* and *cesB,* which are aligned to form a tetradepsipeptide intermediate, followed by the subsequent addition of further didepsipeptide units, with the possibility of “false” chain elongation, thus shedding light on the structure formation of isocereulides E–G [24]. Literature findings show a selectivity of the specialized A domains in CesA1 and CesB1 toward *α*-keto groups over *α*-amino or *α*-hydroxy groups [22]. Furthermore, CesA1 exhibits a high side-chain selectivity and CesB1 shows a loosened side-chain selectivity, which is possibly advantageous to the formation of cereulide homologs [23]. Recent model studies on the thioesterase-dependent macrocyclization of tetradepsipeptide subunits [5] explain the synthesis pathway of isocereulides A–D generated by a single misincorporation of an *α*-hydroxy acid or an *α*-amino acid by the subunits A1 and A2 of CesA or CesB. Probably, these tetradepsipeptide-based observations also build the backbone for the biosynthesis pathway of **2**–**8**, differing only slightly from the cereulide structure with d-Ala substituted by Gly (iCer H), l-Val by l-2-Abu (iCer I), l-*O*-Val by l-*O*-Ala (iCer J), l-*O*-Val by l-*O*-Leu (iCer K), d-*O*-Leu-d-Ala by d-*O*-Ile-d-Ser (iCer L), d-Ala by Gly and d-*O*-Leu by d-*O*-Ile (iCer M), d-*O*-Leu by d-*O*-Ile and d-Ala by d-Ser (iCer N). Overall, balanced structural changes could be observed for the microheterogeneity of cereulide in the amino acid or *α*-hydroxy acid composition in the currently reported isocereulides A–N, with four isocereulides (C, D, H and I) exhibiting a change due to the modification in the amino acid module, five isocereulides (A, B, E, J and K) displaying the modification in one of the *α*-hydroxy acid units and five isocereulides (F, G and L–N) comprising a modification in both subunits.

The occurrence of isoforms of the emetic toxin cereulide (**1**) has been reported in various *B. cereus* strains over the last years. A wide range of structurally similar cereulide variants has been mass-spectrometrically detected [10,11,12,13]. The isocereulides A–G are predicted to be generated simultaneously by Ces-NRPS [12,16], while, in general, the subunit CesB1 is reported to exhibit a loosened side-chain selectivity [23]. Together with the knowledge that, for most isocereulides, only a single misincorporation of an *α*-hydroxy acid or an *α*-amino acid leads to isocereulide formation [5], these circumstances might not only foster the formation of the isocereulides A–G, but of all cereulide homologs detected so far. The very close structural relation between cereulide and the isocereulides and their biosynthesis by the same synthetase gene cluster might build the base for a wide-spread presence of isocereulides in *B. cereus* contaminated samples. Through their varying *m*/*z*, their similar polarity and—compared to cereulide—lower concentrations, the isocereulides might well be not detected during routine analyses due to lacking chromatographic separation or mass spectrometric detection.

The comparison of the cytotoxicity of the purified isocereulides H–N (**2**–**8**) found deviating EC_50_ values ranging from 0.4- to 1.4-fold of the concentration observed for **1**. These findings demonstrate that the cytotoxicity of individual isocereulides cannot be deduced from a sole structure nor from other physicochemical characteristics, such as hydrophobicity. At this point, we can only speculate about the magnitude of the impact that isocereulides have on the overall toxicity of (iso)cereulide-containing *B. cereus* cell cultures. However, keeping in mind that isocereulide A was shown to exhibit an approximately 8-fold and homocereulide a 2.8-fold higher toxicity than cereulide [12,14], it is essential to individually determine the toxic potential of every single isocereulide.

In summary, our study highlights the importance of the isolation and characterization of the structure and bioactivity of cereulide homologs. Furthermore, it shows the importance of including not only cereulide, but also all naturally occurring isocereulides in routine diagnostics to achieve a realistic toxicity evaluation of emetic *B. cereus* in contaminated foods.

## 4. Experimental Section

### General Experimental Procedures

**Chemicals:** The following compounds were commercially obtained: chloroform (anhydrous, ≥99%), d-(+)-glucose monohydrate, methanol-*d_4_*, *N*-isobutyryl-l-cysteine (IBLC), *N*,*N*-diisopropylethylamine (DIPEA), *O*-(Benzotriazol-1-yl)-*N*,*N*,*N*′,*N*′-tetraethyluronium hexafluoro phosphate (HBTU), ortho-phthaldialdehyde (OPA), penicillin-streptomycin, piperidine, potassium hydroxide (KOH), potassium tetraborate tetrahydrate (B_4_K_2_O_7_ × 4H_2_O), pydridine (anhydrous, 99.8%), pyridine-d_5_, sodium hydroxide (NaOH, 1M), (*S*)-(−)-2-hydroxyisocaproic acid (l-*O*-Leu), trifuoroacetic acid (reagent plus, 99%), Trypsin-EDTA solution 10× and MEM-Earle’s with 2.2 g/L NaHCO_3_ with l-alanyl-l-glutamine from Sigma-Aldrich (Steinheim, Germany), fetal calf serum (FCS), Fmoc-d-Ala-Wang resin, Fmoc-Gly-Wang resin, Fmoc-d-Ser-Wang resin, Fmoc-l-Val-Wang resin (100–200 mesh, each), d- and l-alanine, d- and l-serine, d- and l-valine, glycine, HCl (37%), *N*,*N*-dimethylformamide (DMF) and formic acid (HCOOH) from Merck (Darmstadt, Germany), dichloromethane (CH_2_Cl_2_) from Carl Roth (Karlsruhe, Germany), d-lactic-acid (d-*O*-Ala), l-α-hydroxyisovaleric acid (l-*O*-Val), d-α-hydroxyisovaleric acid (d-*O*-Val), d-α-hydroxyisocaproic acid (d-*O*-Leu) from Bachem (Bubendorf, Switzerland), (2*S*,3*S*)-2-hydroxy-3-methylpentanoic acid (l-*O*-Ile) from Interchim (Montluçon Cedex, France), Fmoc-l-2-Abu-Wang resin (100–200 mesh) from Advanced ChemTech (Louisville, KY, USA), (2*R*,3*R*)-2-hydroxy-3-methylpentanoic acid (d-*O*-Ile) from SIA Enamine (Riga, Latvia), (*S*)-(+)-α-methoxy-α-trifluoromethylphenylacetic acid chloride (MTPA) from TCI Deutschland GmbH (Eschborn, Germany), ^13^C_6_-cereulide (>95%) from Chiralix (Nijmegen, Netherlands), phosphate buffered saline (PBS) and sodium pyruvate from Pan Biotech (Aidenbach, Germany), Trypan blue solution 0.4% from Amresco (Darmstadt, Germany) and cell counting kit-8 from Bimake (Munich, Germany).

H_2_O for chromatography was purified with a Milli-Q Reference A+ System (Merck) and solvents were of HPLC or LC-MS grade (J.T. Baker, Deventer, Netherlands).

**Bacterial cultures and growth conditions:** *B. cereus* strains F4810/72 and F4810/72/SCV/AN were used for isolating **1**–**8**. The bacterial cultures were prepared as previously described [37,38]. In brief, pre-cultures were prepared from 3 mL of lysogeny broth, kinetically inoculated with 10^3^ cfu/mL, incubated (24 h, 30 °C, 120 rpm) and harvested by centrifugation (2 min, 8000 rpm). The supernatant was discarded and the remaining cell pellets were autoclaved (15 min, 121 °C). The pellets were stored at −20 °C until further use.

**Solvent extraction of *B. cereus* culture pellets.** Solvent extracts of the cell cultures were prepared as reported recently [13]. The pellets of strains F4810/72 and F4810/72/SCV/AN were thawed, extracted with EtOH by shaking (3 × 30 mL, 1 h, RT, 400 rpm) and centrifuged (10 min, 4000 rpm). The supernatants were membrane-filtrated (0.2 µm; PTFE; Phenomenex, Aschaffenburg, Germany) and all liquids combined; then, the solvent was reduced using a rotary evaporator and stored at −20 °C until further use.

**Purification of cereulide (1) and isocereulides H–N (2–8):** The purification of **1**–**8** from the reduced ethanol extract was performed according to the literature [12,13]. In brief, the extract was diluted with H_2_O (1:10) for better retention of the target analytes on the column material and pre-fractionated via C18 SPE cartridges (60 mL, 10 g; Chromabond, Macherey-Nagel, Düren, Germany). All obtained methanolic fractions were combined and the solvent was reduced to approx. 150 mL using a rotary evaporator and stored at −20 °C until further use for compound isolation. The sample material was separated via semi-preparative HPLC into 10 fractions (Supplementary Materials, Figure S1), for which the respective eluates were combined and their solvents removed through a rotary evaporator. The obtained HPLC fractions were screened for cereulide (**1**) and its isocereulides using a UPLC-TOF-MS system Synapt G2-S (Waters, Manchester, UK) in the positive electrospray mode equipped with a 2.1 × 150 mm, 1.7 µm BEH-C18 column (Waters, Manchester, UK) at a flow rate of 0.4 mL/min at 45 °C using aqueous HCOOH (0.1%) as solvent A and MeCN with HCOOH (0.1%) as solvent B. Elution was performed by starting at 93% B, increasing to 100% B in 4 min, holding at 100% B for 2 min, followed by decreasing to 93% in 0.1 min and holding at 93% B for 0.9 min.

Analytical purification was performed on an HPLC system (Jasco, Groß-Umstadt, Germany) consisting of an HPLC pump (PU 2080 Plus), a degasser (DG-2080-53 3-Line-Degasser), a DAD/UV detector (MD-2010 Plus), coupled with an autosampler (AS-2055 Plus) and equipped with a 250 × 4.6 mm, S-5 µm, YMC-Triart C18 column (YMC Europe, Dinslaken, Germany). The pre-fractionated sample was dissolved in ethanol and separated at a flow rate of 1 mL/min while monitoring the effluent at 220 nm. The eluting substances were manually collected (Supplementary Materials, Figure S2).

**Analysis of dipeptides, resulting from alkaline hydrolysis of cereulide (1) and isocereulides H–N (2–8):** Purified **1**–**8** (~500 µg), respectively, were dissolved in methanolic KOH (1.2 M, 80% MeOH), heated to 50 °C for 2 h; then, their hydrolysates adjusted to pH 5.0 and applied for UPLC-TOF-MS measurement on a Synapt G2-S system (Waters) using the recently reported parameters [12,13]. Chromatography was performed on a 2.1 × 150 mm, 1.7 µm BEH-C18 column (Waters) using aqueous HCOOH (0.1%; Solvent A) and a mixture of MeCN/HCOOH (99.9/0.1; *v*/*v*; Solvent B) at a flow rate of 0.4 mL/min at 45 °C. Chromatography was started at 1% B for 1 min, increased to 10% B in 14 min, increased to 25% B in 10 min and increased to 100% B in 0.5 min, then held at 100% B for 1 min, decreased to 1% B within 0.5 min and kept at 1% B for 1 min for equilibration for the hydrolysates of **1**–**4** and **6**–**8** (*m*/*z* 1175.6679 (**1**); *m*/*z* 1161.6522 (**2**, **3**, **7**); *m*/*z* 1147.6366 (**4**); *m*/*z* 1191.6628 (**6**, **8**)). For the dipeptides obtained from **1** and **5** (*m*/*z* 1189.6835), the gradient was performed according to the literature [13].

**Acidic hydrolysis and analysis of amino acid and α-hydroxy acid entities:** Aliquots (~200 µL) of the alkaline hydrolysates of **1**–**8**, respectively, were further used for acidic hydrolysis (3 mL of 6 M HCl, 24 h, 110 °C), adjusted to pH 7.0 with NaOH (1 M) and freeze-dried [12]. The lyophilisates were separated into the amino acid-containing and *α*-hydroxy acid-containing phases, as has been reported recently [13]. Both aliquots of the acidic hydrolysate of **1**–**8**, respectively, were derivatized enantioselectively to determine the stereochemistry of the single amino acid and α-hydroxy acid units contained in the chemical structures. The derivatization of the amino acids in the sample hydrolysates (**1**–**8**) and the corresponding reference amino acids Gly, d/l-Ser, d/l-Ala, d/l-2-Abu and d/l-Val (50 µmol, each) was performed using OPA and IBLC, followed by SPE purification [12,13,39]. The methanolic eluate from the SPE purification was analyzed through UPLC-TOF-MS using the mass spectrometric parameters described for alkaline hydrolysis. For **2**–**5**, chromatography was performed according to the literature [13] at 45 °C and a flow rate of 0.4 mL/min on a 2.1 × 150 mm, 1.7 µm BEH-C18 column (Waters) with aqueous HCOOH (0.1%; solvent A) and a mixture of MeCN/HCOOH (99.9/0.1; *v*/*v*; solvent B) while starting the gradient at 30% B for 3 min, increasing to 33% B in 3 min, 50% B in 6 min and 100% B in 1 min, followed by holding 100% B for 1 min, decreasing to 30% B within 0.5 min and ending with an equilibration at 30% B for 1.5 min. For **6**–**8**, the analysis was operated by starting the gradient at 10% B for 3 min, increasing to 25% B in 3 min, 40% B in 6 min and 100% B in 2 min, followed by holding 100% B for 1 min, decreasing to 10% B within 0.1 min and ending with an equilibration step at 10% B for 0.9 min. The mass spectrometric data obtained during the analysis of the amino acid isoindole derivatives are stated for Gly, d/l-Ser and d/l-2-Abu and were recently reported for d/l-Ala and d/l-Val [13]:Gly derivative: accurate mass *m*/*z* 363.1012; Δ (ppm) −0.8; calcd *m*/*z* 363.1015 (C_17_H_19_N_2_O_5_S). d-Ser derivative: accurate mass *m*/*z* 393.1120; Δ (ppm) −3.3; calcd *m*/*z* 393.1107 (C_18_H_21_N_2_O_6_S). l-Ser derivative: accurate mass *m*/*z* 393.1120; Δ (ppm) −2.5; calcd *m*/*z* 393.1110 (C_18_H_21_N_2_O_6_S). d-2-Abu derivative: accurate mass *m*/*z* 391.1330; Δ (ppm) +0.5; calcd *m*/*z* 391.1328 (C_19_H_23_N_2_O_5_S). l-2-Abu derivative: accurate mass *m*/*z* 391.1329; Δ (ppm) +0.3; calcd *m*/*z* 391.1328 (C_19_H_23_N_2_O_5_S).

The derivatization of α-hydroxy acids gained from the acidic hydrolysates of **1–8** was performed by applying *S*-(+)-MTPA chloride [13,40]. Anhydrous pyridine (1 µL) was added to enantiomeric pure reference acids d/l-*O*-Ala, d/l-*O*-Val, d/l-*O*-Leu and d/l-*O*-Ile (~0.5 µmol, each) and the sample material of **1**–**8**, respectively, followed by dissolution in anhydrous chloroform (CHCl_3_, 100 µL), supplementing *S*-(+)-MTPA (1 µL) and stirring (1 h, RT). After dilution (1:100; MeCN), the solution was analyzed via UPLC-TOF-MS using MS parameters identical to those described for analyzing dipeptides with the following solvent gradient that started at 30% B, held the initial conditions for 1 min, then increased to 50% B in 10 min, 53% B within 5 min and 100% B in 1 min, held at 100% B for 1 min, decreased in 0.5 min to 30% B and held isocratically for 1.5 min. The analytical data obtained for the individual α-hydroxy acid MTPA esters for d/l-*O*-Val, d/l-*O*-Leu and d/l-*O*-Ile have been reported recently [13]. For d/l-*O*-Ala, the data are the following: d-*O*-Ala derivative: accurate mass *m*/*z* 305.0634; Δ (ppm) −1.0; calcd *m*/*z* 305.0637 (C_13_H_12_F_3_O_5_). l-*O*-Ala derivative: accurate mass *m*/*z* 305.0636; Δ (ppm) −0.3; calcd *m*/*z* 305.0637 (C_13_H_12_F_3_O_5_).

**HEp-2 cell assay:** The cytotoxic activity of **1**–**8** was determined by quantifying their EC_50_ using HEp-2 cells, as described previously [41,42]. In a 96-well microtiter plate assay, a 2% ethanolic minimum essential medium (MEM-Earle’s), containing 2% FCS, 1% sodium pyruvate and 0.4% penicillin-streptomycin (*v*/*v* each), was provided to perform stepwise dilution of each toxin solution. As an overall toxicity reference and cell control, the structurally related toxin valinomycin was used analogously, in a range of approx. 2–500 ng/mL. The composition of each toxin sample is listed in the Supplementary Materials, Table S2. The HEp-2 cells were freed from their medium and washed with 5 mL of PBS twice and 5 mL of 0.05% Trypsin/EDTA solution was added. After incubation (8 min, 37 °C, 5% CO_2_), 10 mL of MEM was added and the cells were resuspended, transferred to a falcon tube and centrifuged (RT, 7 min, 700 rpm). The supernatant was discarded, the cell pellet was suspended in 5 mL of MEM and the cell count was determined via Trypan blue. Finally, the cell count was set to 6.7 × 10^5^ cells/mL MEM (1 × 10^5^ cells/well); a total of 150 µL of this solution was spiked to each well and the plate was incubated (48 h, 37 °C, 5% CO_2_). After removing 100 µL of the medium, 10 µL of the cell proliferation reagent WST (cell counting kit) was added and shaken for 1 min (400 rpm) and the plate was incubated (20 min, 37 °C, 5% CO_2_). To determine cell viability, the extinction at 450 nm/620 nm (TECAN infinite F200) was measured. The respective EC_50_ values for each sample were then calculated from the obtained dose-response curves and the corresponding applied sample dilution and concentration.

**Mass spectrometry:** High-resolution mass spectrometry (UPLC-ESI-TOF-MS) was performed on a Waters Synapt G2-S HDMS spectrometer combined with an Acquity UPLC core system (Waters, Milford, MA, USA) and MS^n^ measurements were conducted on a Bruker Daltonics HCTultra PTM Discovery System (Bruker Daltonics Billerica, MA, USA) according to literature protocol [12].

An aliquot of 2 µL of the respective samples with ^13^C_6_-cereulide as an internal standard (100 ng/mL) was applied for the quantitation of **1–8** in the samples, later applied in the HEp-2 cell assay according to the literature [33], performed on a Waters Xevo TQ-S mass spectrometer (Waters) coupled to an Acquity UPLC i-class core system (Waters) with a binary solvent manager, sample manager and column oven. The system was operated with MassLynx 4.1 SCN 813 Software (Waters) and data processing and analysis were performed with TargetLynx (Waters). The recently reported quantitation method for cereulide and isocereulides A-G [33] was extended by implementing the ammonium adducts of **2**–**8** using the multiple reaction monitoring mode with the following parameters. Compounds **2**, **3** and **7** *m*/*z* 1156.6 → qualifier *m*/*z* 172.2, 314.2; quantifier *m*/*z* 357.2. Compound **4** *m*/*z* 1142.6 → qualifier *m*/*z* 172.2, 314.2; quantifier *m*/*z* 357.2. Compound **5** *m*/*z* 1184.6 → qualifier *m*/*z* 172.2, 314.2; quantifier *m*/*z* 357.2. Compounds **6** and **8** *m*/*z* 1186.6 → qualifier *m*/*z* 172.2, 314.2; quantifier *m*/*z* 357.2. The calibration for the quantitation of the analytes consisted of mixtures of cereulide (0.1–1000 ng/mL in EtOH) and ^13^C_6_-cereulide (100 ng/mL) as an internal standard. Mixtures were prepared from stock solutions and analyzed in triplicate by UPLC-MS/MS. The calibration curve was obtained by plotting the peak area ratios of analytes to internal standard against the concentration ratios of analyte to internal standard for each solution and applying linear regression (origin excluded) to give the equation y=2.5174x+0.0061 with $R^{2}=0.9998$. For **1** and ^13^C_6_-cereulide, the quantifier mass transitions were selected as *m*/*z* 1170.7 → *m*/*z* 357.2 and *m*/*z* 1176.7 → *m*/*z* 358.2, respectively. All mass spectrometric and chromatographic parameters were used as reported [33].

The CCS values were determined using UPLC-ESI-TWIMS-TOF MS on a Waters Vion HDMS mass spectrometer (Waters) coupled to an Acquity i-class UPLC system (Waters) according to reference [43], whereas we used chromatographic parameters equal to the quantitation of **1**–**8**. The scan time for the HDMS^e^ method was set to 0.2 s and the analyses were performed in positive ESI sensitivity mode by applying the following ion source parameters: capillary voltage of 1.5 kV, source temperature of 150 °C, desolvation temperature of 550 °C, cone gas flow of 50 L/h, desolvation gas of 1100 L/h and the collision energy ramp for HDMS^e^ was set from 20 to 60 eV. The calibration of the mass spectrometer was performed in the *m/z* range from 20 to 2000 using MajorMix™ (Waters). All data were lock-mass corrected on the pentapeptide leucine enkephalin (Tyr-Gly-Gly-Phe-Leu, *m*/*z* 556.2771; [M + H]^+^) supplied as a solution (50 pg/µL) of MeCN/0.1% HCOOH (1/1, *v*/*v*). The scan time for the lock mass was set to 2.0 s with an interval of 0.5 min. The operation of the UPLC and Vion systems, as well as data processing, was performed with UNIFI™ Software version 3.1.2 (Waters).

**Nuclear magnetic resonance spectroscopy:** NMR spectra were recorded on a 400 MHz Avance III spectrometer with a Broadband Observe BBFO plus, a 500 MHz Avance NEO spectrometer with a cryo probe CTCI (^1^H/^13^C/^15^N) and a 600 MHz Avance NEO spectrometer with a cryo probe TCI 600S3 H-C/N-D-05 Z XT (Bruker, Rheinstetten, Germany). The chemical shift was referenced to the solvent signal, MeOH-*d_4_* and pyridine-*d_5_*, respectively. Data processing and evaluation were performed using Topspin Software Version 4.0.7 (Bruker, Rheinstetten, Germany).

**Figure 5:**
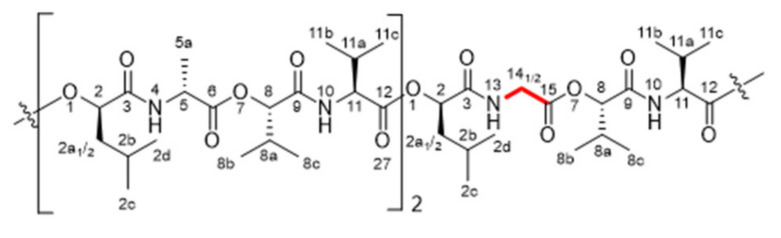
Chemical structure of isocereulide H (**2**) with numbering used for assigned NMR signals.

**Figure 6:**
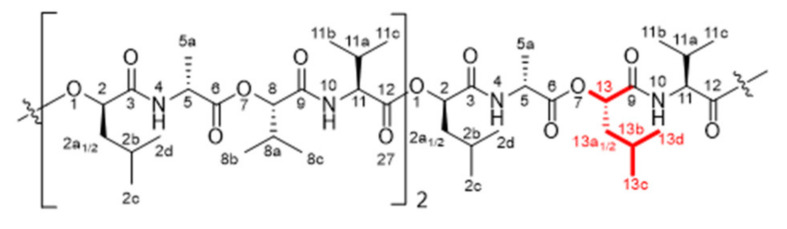
Chemical structure of isocereulide K (**5**) with numbering used for assigned NMR signals.

**Figure 7:**
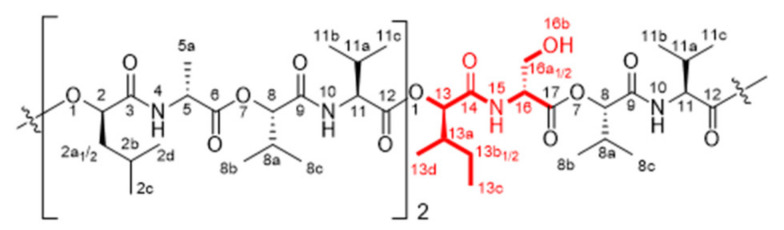
Chemical structure of isocereulide L (6) with numbering used for assigned NMR signals.

**Figure 8:**
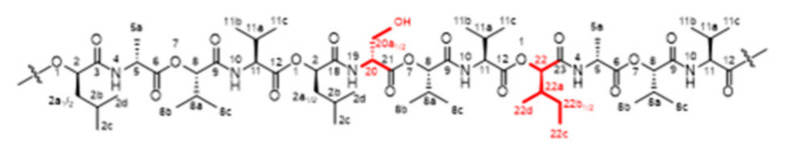
Chemical structure of isocereulide N (**8**) with numbering used for assigned NMR signals.

**Figure 9:**
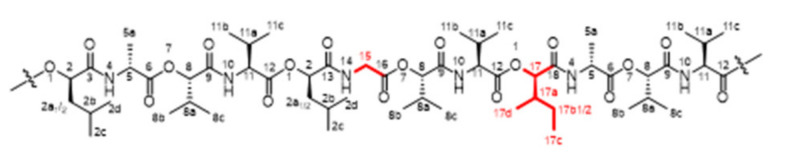
Chemical structure of isocereulide M (**7**) with numbering used for assigned NMR signals.

Chemical structures and NMR data of isocereulide H (**2**) (Figure 5), K (**5**) (Figure 6), L and N (**6** + **8**) (Figure 7 and Figure 8) and M (**7**) (Figure 9) are highlighted in the following:

**Isocereulide H (2)**

^1^H-NMR [600 MHz, pyridine-*d_5_*, 298K]: δ (ppm) 0.83–0.94 [m, 9H, H_3_-C(2c)], 0.84–1.00 [m, 9H, H_3_-C(2d)], 1.00–1.19 [m, 36H, H_3_-C(8b, 8c, 11b, 11c)],1.65 [d, 3H, H_3_-C(5a)], 1.70 [d, 3H, H_3_-C(5a)], 1.81–2.02 [m, 6H, H-C(2a_1_, 2b)], 2.02–2.19 [m, 3H, H-C(2a_2_)], 2.36–2.61 [m, 6H, H-C(8a, 11a)], 4.13–4.24 [m, 1H, H-C(14_1_)], 4.44–4.54 [m, 1H, H-C(14_2_)], 4.60–4.68 [s, 1H, H-C(11)], 4.73–4.84 [m, 4H, H-C(5, 11)], 5.29–5.34 [m, 1H, H-C(8)], 5.38–5.46 [m, 2H, H-C(8)], 5.55–5.62 [m, 1H, H-C(2)], 5.62–5.70 [m, 2H, H-C(2)], 8.60–8.68 [m, 1H, H-N(10)], 8.86–8.91 [m, 1H, H-N(13)], 8.97–9.06 [m, 2H, H-N(10)], 9.08–9.14 [m, 1H, H-N(4)], 9.25–9.30 [m, 1H, H-N(4)]. ^13^C-NMR [150 MHz, pyridin-*d_5_*, 298K]: δ (ppm) 17.2 [C(5a)], 17.5 [C(5a)], 17.7 [C(8b/8c/11b/11c)], 18.0 [2C, C(8b/8c/11b/11c)], 19.18 [2C, C(8b/8c/11b/11c)], 19.23 [C(8b/8c/11b/11c)], 19.3 [2C, C(8b/8c/11b/11c)], 19.4 [C(8b/8c/11b/11c)], 19.7 [C(8b/8c/11b/11c)], 19.8 [2C, C(8b/8c/11b/11c)], 21.7 [3C, C(2c)], 23.6 [2C, C(2d)], 23.7 [C(2d)], 25.1 [3C, C(2b)], 30.5 [2C, C(11a)], 30.7 [C(8a)], 31.3 [C(8a)], 31.4 [C(11a)], 31.6 [C(8a)], 41.6 [2C, C(2a)], 41.8 [C(2a)], 42.1 [C(14)], 49.9 [C(5)], 50.0 [C(5)], 59.4 [2C, C(11)], 59.8 [C(11)], 73.56 [C(2)], 73.63 [C(2)], 74.1 [C(2)], 79.1 [C(8)], 79.4 [C(8)], 79.5 [C(8)], 169.9 [C(15)], 171.0 [3C, C(9)], 171.6 [C(12)], 171.8 [C(3)], 172.08 [C(12)], 172.13 [C(12)], 172.17 [2C, C(3)], 172.9 [C(6)], 173.1 [C(6)].

**Isocereulide K (5)**

^1^H-NMR [500 MHz, pyridine-*d_5_*, 298K]: δ (ppm) 0.82–1.06 [m, 24H, H_3_-C(2c, 2d, 13c, 13d)], 1.06–1.24 [m, 30H, H_3_-C(8b, 8c, 11b, 11c)], 1.65–1.77 [m, 9H, H_3_-C(5a)], 1.89–2.05 [m, 8H, H-C(2a_1_, 2b, 13a_1_, 13b)], 2.05–2.19 [m, 4H, H-C(2a_2_, 13a_2_)], 2.42–2.54 [m, 3H, H-C(11a)], 2.55–2.65 [m, 2H, H-C(8a)], 4.69–4.77 [m, 2H, H-C(11)], 4.80–4.86 [m, 4H, H-C(5, 11)], 5.41–5.46 [m, 1H, H-C(8)], 5.48–5.52 [m, 1H, H-C(8)], 5.56–5.60 [m, 1H, H-C(13)], 5.62–5.70 [m, 3H, H-C(9)], 8.87–8.93 [dd, 1H, *J* = 5.8, 6.1 Hz, H-N(10)], 8.93–8.98 [d, 1H, *J* = 6.9 Hz, H-N(10)], 9.05–9.20 [d, 1H, *J* = 7.2 Hz, H-N(10)], 9.15–9.20 [d, 1H, J = 5.5 Hz, H-N(4)], 9.21–9.29 [m, 2H, H-N(4)].

**Isocereulide L (6) and Isocereulide N (8)**

^1^H-NMR [600 MHz, pyridine-*d_5_*, 298K]: δ (ppm) 0.76–0.91 [m, 18H, H_3_-C(2c/2d, 13c, 22c)], 0.91–1.01 [m, 12H, H_3_-C(2c/2d)], 1.01–1.22 [m, 72H, H_3_-C(8b, 8c, 11b, 11c)], 1.22–1.40 [m, 8H, H_3_-C(13b_1_, 13d, 22b_1_, 22d,)], 1.43–1.58 [m, 2H, H-C(13b_2_, 22b_2_)], 1.58–1.73 [m, 12H, H-C(5a)], 1.83–2.02 [d, 8H, H_3_-C(2a_1_, 2b)], 2.02–2.18 [m, 4H, H-C(2a_2_)], 2.27–2.68 [m, 14H, H-C(8a, 11a, 13a, 22a)], 4.43–4.63 [m, 4H, H-C(16a, 20a)], 4.63–4.92 [m, 12H, H-C(5, 11, 16, 22)], 5.33–5.44 [m, 4H, H-C(8)], 5.51–5.59 [m, 2H, H-C(8)], 5.59–5.68 [m, 4H, H-C(2, 13/22)], 5.75 [s, 2H, H-C(2, 13/22)], 8.79–9.44 [m, 12H, H-N(4, 10, 15, 19)].

**Isocereulide M (7)**

^1^H-NMR [600 MHz, pyridine-*d_5_*, 298K]: δ (ppm) 0.83–0.93 [m, 9H, H_3_-C(2c/2d, 17c)], 0.93–1.00 [m, 6H, H_3_-C(2c/2d)], 1.00–1.21 [m, 36H, H_3_-C(8b, 8c, 11b, 11c)], 1.21–1.31 [m, 3H, H_3_-C(17d)], 1.31–1.40 [m, 1H, H-C(17b_1_)], 1.47–1.56 [m, 1H, H-C(17b_2_)], 1.67 [d, 3H, *J* = 6.8 Hz, H_3_-C(5a)], 1.74 [d, 3H, *J* = 6.4 Hz, H_3_-C(5a)], 1.83–2.03 [m, 4H, H-C(2a_1_, 2b)], 2.03–2.20 [m, 2H, H-C(2a_2_)], 2.32–2.64 [m, 7H, H-C(8a, 11a, 17a)], 4.02 [dd, 1H, *J* = 4.2, 16.8 Hz, H-C(15_1_)], 4.51 [dd, 1H, J = 4.4, 16.9 Hz, H-C(15_2_)], 4.61 [s, 1H, H-C(11)], 4.73–4.87 [m, 4H, H-C(5, 11)], 5.30 [d, 1H, *J* = 3.8 Hz, H-C(8)], 5.41–5.46 [m, 2H, H-C(8)], 5.62 [s, 1H, H-C(13)], 5.66 [d, 1H, *J* = 2.8 Hz, H-C(2)], 5.67 [d, 1H, *J* = 2.0 Hz, H-C(2)], 8.88 [s, 1H, H-N(14)], 9.02 [m, 2H, H-N(10)], 9.08 [m, 1H, H-N(10)], 9.12 [m, 1H, H-N(4)], 9.16 [m, 1H, H-N(4)].

## Figures and Tables

**Table 1:** UPLC-ESI^–^-TOF-MS data of cereulide (**1**) and its structurally known isocereulides A–G and H–N (**2**–**8**).

No. ^a^	Variant ^b^	F No. ^c^	UPLC-ESI-TOF-MS Data				Structural Modification ^i^	
			RT ^d^ (min)	EM/AM ^e^ (var., ppm)	EC ^f^ mass ^g^ (Da)	Observed CCS ^h^ (Å^2^)	Cereulide	Variant
-	Isocereulide C	I-4, II-4	2.52	1191.66128/1191.6617 (−0.9)	C_57_H_96_N_6_O_19_Na (+16)	355.9	d-Ala	d-Ser
6	Isocereulide L	II-4	2.76	1191.66128/1991.6624 (−0.3)	C_57_H_96_N_6_O_19_Na (+16)	354.8	d-*O*-Leu-d-Ala	d-*O*-Ile-d-Ser
8	Isocereulide N	II-4	2.76	1191.66128/1991.6624 (−0.3)	C_57_H_96_N_6_O_19_Na (+16)	354.8	d-*O*-Leu d-Ala	d-*O*-Ile d-Ser
-	Isocereulide D	I-6	2.88	1147.6366/1147.6381 (+1.3)	C_55_H_92_N_6_O_18_Na (–28)	350.3	l-Val	l-Ala
2	Isocereulide H	II-6, II-7	2.88	1161.6522/1661.6527 (+0.4)	C_56_H_94_N_6_O_18_Na (–14)	353.5	d-Ala	Gly
7	Isocereulide M	II-6, II-7	3.07	1161.6522/1661.6537 (+1.3)	C_56_H_94_N_6_O_18_Na (–14)	347.8	d-*O*-Leu d-Ala	d-*O*-Ile Gly
3	Isocereulide I	I-6	3.40	1161.6522/1161.6538 (+1.4)	C_56_H_94_N_6_O_18_Na (–14)	354.3	l-Val	l-2-Abu
4	Isocereulide J	I-6	3.57	1147.6366/1147.6364 (−0.2)	C_55_H_92_N_6_O_18_Na (–28)	347.4	l-*O*-Val	l-*O*-Ala
-	Isocereulide B	II-7	3.83	1161.6522/1161.6523 (+0.1)	C_56_H_94_N_6_O_18_Na (–14)	351.2	d-*O*-Leu	d-*O*-Val
-	Isocereulide E	II-7	3.83	1161.6522/1161.6523 (+0.1)	C_56_H_94_N_6_O_18_Na (–14)	350.1	d-*O*-Leu	*O*-Ile
1	Cereulide	I-8, II-8	4.25	1175.6679/1175.6677 (−0.2)	C_57_H_96_N_6_O_18_Na (–)	354.6	-	-
-	Isocereulide G	I-9, II-9	4.56	1175.6679/1175.6676 (−0.3)	C_57_H_96_N_6_O_18_Na (–)	352.4		CI
5	Isocereulide K	I-9, II-9	4.59	1189.6835/1189.6842 (+0.6)	C_58_H_98_N_6_O_18_Na (+14)	361.1	l-*O*-Val	l-*O*-Leu
-	Isocereulide A	I-9, II-9	4.83	1189.6835/1189.6841 (+0.5)	C_58_H_98_N_6_O_18_Na (+14)	359.6	l-*O*-Val	l-*O*-Ile
-	Isocereulide F	I-9	5.00	1189.6835/1189.6843 (+0.7)	C_58_H_98_N_6_O_18_Na (+14)	358.1	d-*O*-Leu-d-Ala	l-*O*-Val-l-Val

CI constitutional isomer. ^a^ Compound number of detected cereulide variants according to the order of reference in the text; isocereulide L and N (**6** + **8**) were isolated as a mixture (approx. 42/58). ^b^ The newly identified amino acid and *α*-hydroxy acid sequences of **2**–**8** are pictured in Figure 3a; the structures of isocereulides A–G are given in literature [12,13]. ^c^ HPLC fraction of *B. cereus* strain culture extract F4810/72 (I) and F4810/72/SCV/AN (II) with the detection of the respective target compounds. Compounds **1**–**8** were isolated from given fractions. Numbers of fractions are given according to the semi-preparative HPLC-fractionation shown in Supplementary Materials Figures S1 and S2. ^d^ Retention time on RP-BEH-C18 UPLC column (Waters). ^e^ Exact mass (EM), calculated from the elemental composition, and accurate mass (AM) of pseudomolecular ions [M + Na]^+^ of the analyte, determined via UPLC-ESI^+^-TOF-MS. ^f^ Elemental composition of the analyte. ^g^ Mass difference between cereulide and the target variant. ^h^ Observed CCS value detected for [M + K]^+^ adduct, due to the preferred K^+^ complexation in a natural environment. ^i^ The listed amino or α-hydroxy acids in cereulide are replaced by the ones enlisted for each variant, with CI being a constitutional isomer.

**Table 2:** UPLC-ESI^–^-TOF-MS data of dipeptides released after alkaline hydrolysis from cyclic cereulide (**1**) and isocereulides H–N (**2**–**8**).

Variant ^a^	Number of ^b^		Peptide Ratio ^c^	Additional Dipeptide			
	d-*O*-Leu-d-Ala	l-*O*-Val-l-Val		AM (*m*/*z*) ^d^ (var., ppm)	EM (*m*/*z*) ^e^	EC ^f^	Sequence
Cereulide (**1**)	3	3	0.78				
Isocereulide H (**2**)	2	3	0.24	188.0921 (−1.1)	188.0923	C_8_H_14_NO_4_	d-*O*-Leu-Gly
Isocereulide I (**3**)	3	2	0.66	202.1080 (+0.5)	202.1079	C_9_H_16_NO_4_	l-*O*-Val-l-2-Abu
Isocereulide J (**4**)	3	2	0.89	188.0925 (+1.1)	188.0923	C_8_H_14_NO_4_	l-*O*-Ala-l-Val
Isocereulide K (**5**)	3	2	1.23	230.1397 (+2.2)	230.1392	C_11_H_20_NO_4_	l-*O*-Leu-l-Val
Isocereulide L ^g^ (**6**)	2	3	0.30	218.1023 (−2.3)	218.1028	C_9_H_16_NO_5_	d-*O*-Ile-d-Ser
Isocereulide M (**7**)	1	3	0.24	188.0920 (−1.6) 202.1078 (−0.5)	188.0923 202.1079	C_8_H_14_NO_4_ C_9_H_16_NO_4_	d-*O*-Leu-Gly d-*O*-Ile-d-Ala
Isocereulide N ^g^ (**8**)	1	3	0.30	218.1023 (−2.3) 202.1076 (−1.5)	218.1028 202.1079	C_9_H_16_NO_5_ C_9_H_16_NO_4_	d-*O*-Leu-d-Ser d-*O*-Ile-d-Ala

^a^ Determined amino acid and α-hydroxy acid sequences of **1** and **2**–**8** are presented in Figure 3a. ^b^ Number of dipeptides d-*O*-Leu-d-Ala (AM *m*/*z* 202.1082 (+1.5 ppm); EM *m*/*z* 202.1079; C_9_H_16_NO_4_) and l-*O*-Val-l-Val (AM *m*/*z* 216.1239 (+1.4 ppm); EM *m*/*z* 216.1236; C_10_H_18_NO_4_) in the dodecadepsipeptide. ^c^ The peptide ratio is given as analyzed molar ratio, which was calculated by the quotient of peak area ratios of the peptides d-*O*-Leu-d-Ala and l-*O*-Val-l-Val from UPLC-TOF-MS analysis after alkaline hydrolysis. ^d^ AM giving the accurate mass of the [M − H]^–^ pseudomolecular ion obtained via UPLC-ESI-TOF-MS. ^e^ EM giving the exact mass computed for the [M − H]^–^ pseudomolecular ion of the analyte. ^f^ Elemental composition of the respective target dipeptide. ^g^ Compounds **6** and **8** were isolated as a mixture of approx. 42% **6** and 58% **8**. Percentage based on the quotient of the peak area ratio of their peptides d-*O*-Leu-d-Ser and d-*O*-Ile-d-Ser.

## Data Availability

The data presented in this study are available in the Supplementary Materials.

## Supplementary Materials

Additional information about the isolation of cereulide (**1**) and isocereulides H–N (**2**–**8**); synthesis and characterization of reference dipeptides; data on alkaline and acidic hydrolysis and MS^n^ sequencing of **1**–**8**; 1D- and 2D-NMR data on isocereulide H (**2**), K (**5**), L and N (**6** + **8**) and M (**7**); and further information about the samples used in the HEp-2 cell assay are available as Supplementary Materials. Figure S1: Semi-preparative RP-C18-HPLC separation of the ethanolic cell extract from strain F4810/72 (I) and UPLC-ESI^+^-TOF-MS detection of isocereulide H (**2**), I (**3**), J (**4**), and B and E from HPLC fraction I-6. Figure S2: Chromatograms of the analytical HPLC separation of (**a**) fraction 4 of strain F4810/72/SCV/AN for isolation of isocereulides L and N (**6, 8**), (**b**) fraction 6 of strain F4810/72/SCV/AN for isolation of isocereulides H and M (**2, 7**), (**c**) fraction 6 of strain F4810/72 for isolation of isocereulides I and J (**3, 4**), (**d**) fraction 7 of strain F4810/72/SCV/AN for isolation of cereulide (**1**), isocereulides H and M (**2, 7**), and (**e**) fraction 9 of strain F4810/72 for isolation of cereulide (**1**) and isocereulide K (**5**). Figure S3: Structures of synthesized reference dipeptide units, with numbered atoms for assignment of NMR data. Figure S4.1: Mass spectrometric fragmentation pattern (UPLC-ESI-TOF-MS^e^) of dipeptides (**a**) d-*O*-Leu-d-Ala, and (**b**) l-*O*-Val-l-Val present in cereulide (**1**), (**c**) d-*O*-Leu-Gly present in isocereulide H (**2**) and M (**7**), (**d**) l-*O*-Val-l-2-Abu present in isocereulide I (**3**), and (**e**) l-*O*-Ala-l-Val present in isocereulide J (**4**). Figure S4.2: Mass spectrometric fragmentation pattern (UPLC-ESI-TOF-MS^e^) of dipeptides (**f**) l-*O*-Leu-l-Val present in isocereulide K (**5**), (**g**) d-*O*-Ile-d-Ser present in isocereulide L (**6**), (**h**) d-*O*-Ile-d-Ala present in isocereulide M (**7**) and N (**8**), and (**i**) d-*O*-Leu-d-Ser present in isocereulide N (**8**). Figure S5: UPLC-ESI^–^-TOF-MS-chromatograms of alkaline hydrolysis of (**a**) cereulide (**1**), (**b**) isocereulide H (**2**), (**c**) **2** spiked with l-*O*-Leu-Gly, and (**d**) **2** spiked with l-*O*-Ile-Gly. Figure S6: UPLC-ESI^–^-TOF-MS-chromatograms of alkaline hydrolysis of (**a**) cereulide (**1**), (**b**) isocereulide I (**3**), (**c**) **3** spiked with l-*O*-Val-l-2-Abu, and (**d**) **3** spiked with d-*O*-Val-l-2-Abu. Figure S7: UPLC-ESI^–^-TOF-MS-chromatograms of alkaline hydrolysis of (**a**) cereulide (**1**), (**b**) isocereulide J (**4**), (**c**) **4** spiked with l-*O*-Ala-l-Val, and (**d**) **4** spiked with d-*O*-Ala-l-Val. Figure S8: UPLC-ESI^–^-TOF-MS-chromatograms of alkaline hydrolysis of (**a**) cereulide (**1**), (**b**) isocereulide K (**5**), (**c**) **5** spiked with l-*O*-Leu-l-Val, (**d**) **5** spiked with l-*O*-Ile-l-Val, (**e**) **5** spiked with d-*O*-Leu-l-Val, and (**f**) **5** spiked with d-*O*-Ile-l-Val. Figure S9: UPLC-ESI^–^-TOF-MS-chromatograms of alkaline hydrolysis of (**a**) cereulide (**1**), (**b**) isocereulide L/N (**6 + 8**), (**c**) **6 + 8** spiked with l-*O*-Leu-d-Ala, (**d**) **6 + 8** spiked with d-*O*-Leu-d-Ala, (**e**) **6 + 8** spiked with l-*O*-Ile-d-Ala, (**f**) **6 + 8** spiked with d-*O*-Ile-d-Ala, (**g**) **6 + 8** spiked with d-*O*-Leu-l-Ser, (**h**) **6 + 8** spiked with d-*O*-Leu-d-Ser, (**i**) **6 + 8** spiked with l-*O*-Ile-d-Ser, (**j**) **6 + 8** spiked with d-*O*-Ile-d-Ser. Figure S10: UPLC-ESI^–^-TOF-MS-chromatograms of alkaline hydrolysis of (**a**) cereulide (**1**), (**b**) isocereulide M (**7**), (**c**) **7** spiked with l-*O*-Ile-Gly, (**d**) **7** spiked with l-*O*-Leu-Gly, (**e**) **7** spiked with l-*O*-Leu-d-Ala, (**f**) **7** spiked with d-*O*-Leu-d-Ala, (**g**) **7** spiked with l-*O*-Ile-d-Ala, and (**h**) **7** spiked with d-*O*-Ile-d-Ala. Figure S11: UPLC-ESI^–^-TOF-MS-chromatograms of acidic hydrolysis after chiral amino acid derivatization of (**a**) amino acid references glycine, l- and d-alanine, l- and d-2-aminobutyric acid, and l- and d-valine, (**b**) cereulide (**1**), (**c**) isocereulide H (**2**), (**d**) isocereulide I (**3**), (**e**) isocereulide J (**4**), and (**f**) isocereulide K (**5**). Figure S12: UPLC-ESI^–^-TOF-MS-chromatograms of acidic hydrolysis after chiral amino acid derivatization of (**a**) amino acid references l- and d-serine, glycine, l- and d-alanine, and l- and d-valine, (**b**) cereulide (**1**), (**c**) isocereulide L and N (**6 + 8**), (**d**) isocereulide M (**7**). Figure S13: UPLC-ESI^–^-TOF-MS-chromatograms of acidic hydrolysis after chiral *α*-hydroxy acid derivatization of (**a**) *α*-hydroxy acid references l- and d-*O*-alanine, l- and d-*O*-valine, and l- and d-*O*-leucine with l-*O*-isoleucine, (**b**) cereulide (**1**), (**c**) isocereulide H (**2**), (**d**) isocereulide I (**3**), (**e**) isocereulide J (**4**), and (**f**) isocereulide K (**5**). Figure S14: UPLC-ESI^–^-TOF-MS-chromatograms of acidic hydrolysis after chiral *α*-hydroxy acid derivatization of (**a**) *α*-hydroxy acid references l- and d-*O*-valine, and l- and d-*O*-leucine with l- and d-*O*-isoleucine, (**b**) cereulide (**1**), (**c**) isocereulide L and N (**6 + 8**), (**d**) isocereulide M (**7**). Figure S15: ^1^H-NMR spectrum of **2** (600 MHz, 298 K, pyridine-*d_5_*). Figure S16: ^1^H, ^1^H-COSY-NMR spectrum of **2** (600 MHz, 298 K, pyridine-*d_5_*). Figure S17: ^1^H,^13^C-HSQC-NMR spectrum of **2** (600 MHz, 150 MHz, 298 K, pyridine-*d_5_*). Figure S18: ^1^H,^13^C-HMBC-NMR spectrum of **2** (600 MHz, 150 MHz, 298 K, pyridine-*d_5_*). Figure S19: ^13^C-NMR spectrum of **2** (150 MHz, 298 K, pyridine-*d_5_*). Figure S20: ^1^H-NMR spectrum of **5** (500 MHz, 298 K, pyridine-*d_5_*). Figure S21: ^1^H, ^1^H-COSY-NMR spectrum of **5** (500 MHz, 298 K, pyridine-*d_5_*). Figure S22: ^1^H-NMR spectrum of a mixture of **6** and **8** (600 MHz, 298 K, pyridine-*d_5_*). Figure S23: ^1^H, ^1^H-COSY-NMR spectrum of a mixture of **6** and **8** (600 MHz, 298 K, pyridine-*d_5_*). Figure S24: ^1^H,^13^C-HSQC-NMR spectrum of a mixture of **6** and **8** (600 MHz, 150 MHz, 298 K, pyridine-*d_5_*). Figure S25: ^1^H,^13^C-HMBC-NMR spectrum of a mixture of **6** and **8** (600 MHz, 150 MHz, 298 K, pyridine-*d_5_*). Figure S26: ^1^H-NMR spectrum of **7** (600 MHz, 298 K, pyridine-*d_5_*). Figure S27: ^1^H, ^1^H-COSY-NMR spectrum of **7** (600 MHz, 298 K, pyridine-*d_5_*). Figure S28: ^1^H,^13^C-HSQC-NMR spectrum of **7** (600 MHz, 150 MHz, 298 K, pyridine-*d_5_*). Figure S29: ^1^H,^13^C-HMBC-NMR spectrum of **7** (600 MHz, 150 MHz, 298 K, pyridine-*d_5_*). Figure S30: Representative raw data from HEp-2 cell culture assays of (**a**) cereulide in dilutions 1:10,000 and 1:100,000, (**b**) isocereulide H (**2**) in dilutions 1:1000 and 1:10,000, and (**c**) isocereulide I (**3**) in dilutions 1:1000 and 1:10,000, (**d**) isocereulide J (**4**) in dilutions 1:1000 and 1:10,000 (**e**) isocereulide K (**5**) in dilutions 1:1000 and 1:10,000, (**f**) isocereulide L/N (**6 + 8**) in dilutions 1:100 and 1:1000, and (**g**) isocereulide M (**7**) in dilutions 1:100 and 1:1000. Samples were diluted in EtOH, and each substance was tested in four biological repilca of the two different dilutions. Table S1: Ratios of d/l-amino and d/l-*α*-hydroxy acids in cereulide (**1**) and isocereulides H–N (**2–8**) from UPLC-ESI^–^-TOF-MS data gained after acidic hydrolysis of alkaline hydrolysates. Table S2: Sample composition of purified cereulide (**1**) and isocereulides H–N (**2–8**) for the HEp-2 cell assay.

## Sample Availability

Sample material of cereulide and isocereulides H–N is not available from the authors.
