# Supplementary material for: Bacillus cereus Toxin Repertoire: Diversity of (Iso)cereulide(s)
Source: Molecules. 2022 Jan 27;27(3):872. doi: 10.3390/molecules27030872 (PMC8840689; doi:10.3390/molecules27030872)
Supplement: Supplementary file 1 [file molecules-27-00872-s001.zip › molecules-1535264-supplementary.pdf]

# *Supplementary Materials*

# *Bacillus cereus* Toxin Repertoire: Diversity of (Iso)cereulide(s)


*Veronika Walser[†], Markus Kranzler[‡], Corinna Dawid[†], Monika Ehling-Schulz[‡], Timo D. Stark[†]* and Thomas F. Hofmann[†]*

*[†]Food Chemistry and Molecular Sensory Science, Department of Molecular Life Sciences, School of Life Sciences, Technical University of Munich, Lise-Meitner-Str. 34, 85354 Freising, Germany,*

*[‡]Institute of Microbiology, Department of Pathobiology, University of Veterinary Medicine Vienna, Veterinärplatz 1, 1210 Vienna, Austria.*

Content

A. Isolation of cereulide (**1**) and isocereulides H–N (**2–8**) *via* RP-HPLC

B. Dipeptide synthesis, isolation and characterization *via* 1D- and 2D-NMR data and $MS^e$ fragmentation

C. Identification of the dipeptide units in the alkaline hydrolysates of isocereulides H–N (**2–8**) by co-chromatography

D. Enantioselective amino acid and $\alpha$-hydroxy acid analysis in acidic hydrolysates of cereulide (**1**) and isocereulides H–N (**2–8**), after chiral derivatization

E. 1D- and 2D-NMR spectra of isocereulides H (**2**), K (**5**), L and N (**6+8**), and M (**7**)

F. $MS^n$-data of cereulide (**1**) and isocereulides H–N (**2–8**)

G. Toxin composition of samples used in HEp-2 cell assay and their $EC_{50}$ values

H. References

A. Isolation of cereulide (**1**) and isocereulides H–N (**2–8**) *via* RP-HPLC

Semi-preparative HPLC fractionation was performed according to literature on a PrepStar system (Varian, Darmstadt, Germany) consisting of two HPLC-pumps (model SD-1), a two-wavelength UV detector (Prostar 325), a fraction collector (model 701), and equipped with a 250x10mm, 4 $\mu$m, 90 Å Jupiter Proteo column (Phenomenex, Aschaffenburg, Germany).[1,2] Chromatography was performed at a flow rate of 4.2 mL using $H_2O$ (Solvent A) and MeOH (Solvent B), starting with 85% B for 1 min, increasing to 92% B in 15 min, holding 92% B for 9 min, increasing to 100% B within 1 min, holding 100% B for 9 min, and decreasing to 85% B in 1 min followed by equilibration for 2 min. The effluent was monitored at a wavelength of 220 nm. The sample material was separated into 10 fractions (**Figure S1**), for which the respective eluates were combined and their solvents removed with a rotary evaporator. Water (15 mL) was added, the fractions were freeze-dried twice and were stored until further fractionation at -20 °C.

All gathered *B. cereus* cell material from strains F4810/72 (I) and F4810/72/SCV/AN (II), respectively, were combined and the isocereulides located in the following semi-preparative HPLC-fractions: cereulide (**1**; fraction I-8, II-8), isocereulide A (fraction I-9, II-9), isocereulide B (fraction II-7), isocereulide C (fraction I-4, II-4), isocereulide D (fraction I-6), isocereulide E (fraction II-7), isocereulide F (fraction I-9), isocereulide G (fraction I-9, II-9), isocereulide H (**2**; fraction II-6, II-7), isocereulide I (**3**; fraction I-6), isocereulide J (**4**; fraction I-6), isocereulide K (**5**; fraction I-9, II-9), isocereulide L and N (**6+8**; fraction II-4), and isocereulide M (**7**; fraction II-6, II-7). Exemplary, a chromatogram of the semi-preparative HPLC fractionation, combined with UPLC-ESI$^+$-TOF-MS analysis of fraction I-6, is pictured (**Figure S1**). **1**–**8**, respectively, were then purified from the given fractions *via* analytical HPLC (**Figure S2**). All analytes exhibited their maximum absorbance in UV-vis detection ($UV_{max}$) at $\lambda_{max}$ = 204 nm, which is in alignment with the recently published data on cereulide.[3]

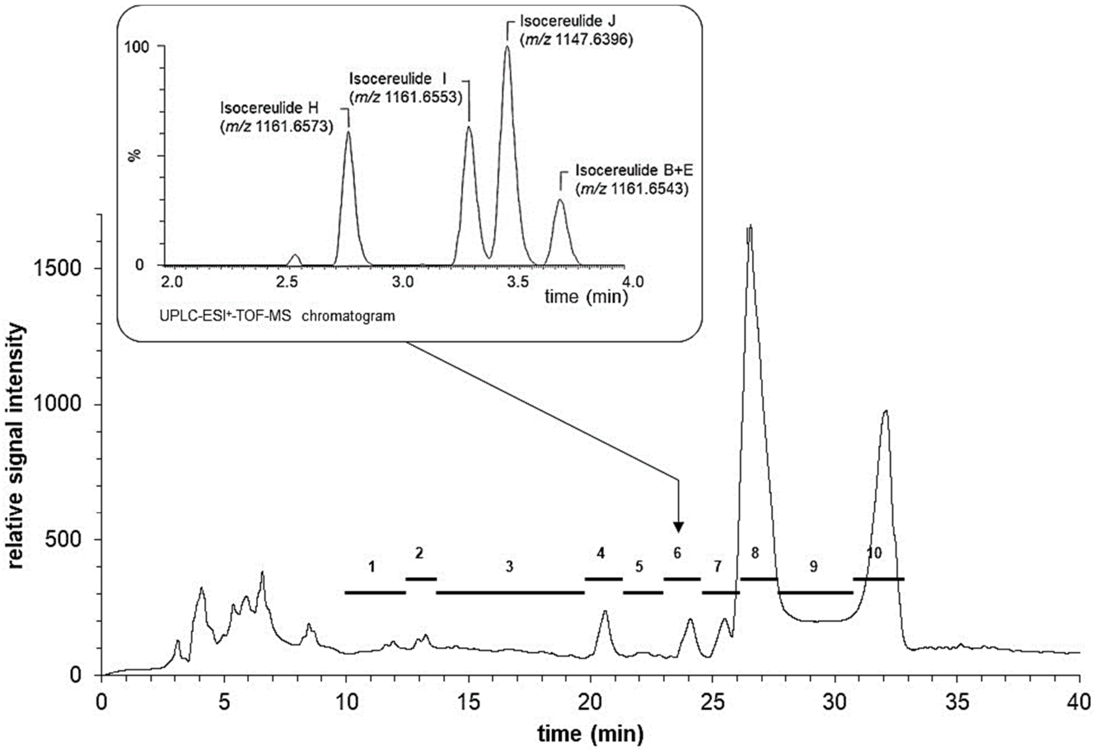


**Figure S1.** Semi-preparative RP-C18-HPLC separation of the ethanolic cell extract from strain F4810/72 (I) and UPLC-ESI$^+$-TOF-MS detection of isocereulide H (**2**), I (**3**), J (**4**), and B and E from HPLC fraction I-6.

Yields of the respective fractions after freeze drying were determined by weighing and are exemplary listed for the work up of one batch of *B. cereus* strain F4810/72/SCV/AN (II): fractions 1 –3: no weight could be determined; fraction 4: 1,1 mg; fraction 5: 0,5 mg; fraction 6; 1,8 mg; fraction 7: 4,3 mg; fraction 8: 34,7 mg; fraction 9: 7,9 mg; fraction 10: 4,7 mg.

The purified isocereulides of all batches combined were not weighed due to their low amounts and the high expected water contents. The isocereulides were solved in EtOH and were quantified by means of UPLC-MS/MS. For the isolated isocereulides the following yields were obtained:

isocereulide H (**2**): 1365.77 (±27.27) $\mu$g; isocereulide I (**3**): 106.15 (±1.76) $\mu$g; isocereulide J (**4**): 139.32 (±1.10) $\mu$g; isocereulide K (**5**): 405.84 (±3.40) $\mu$g; isocereulide L+N (**6+8**): 433.15 (±4.20) $\mu$g; isocereulide M (**7**): 454.18 (±7.68) $\mu$g.

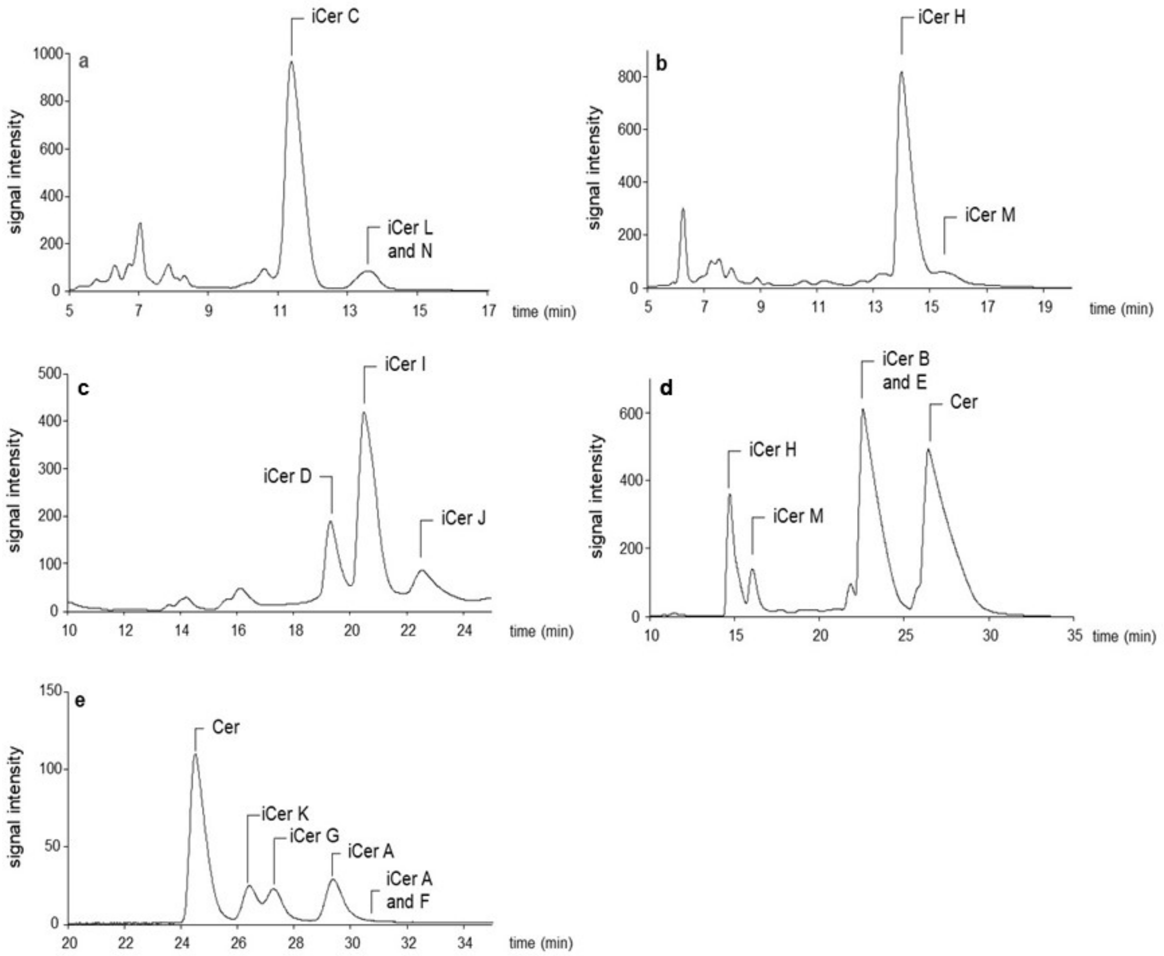


**Figure S2.** Chromatograms of the analytical HPLC separation of (**a**) fraction 4 of strain F4810/72/SCV/AN for isolation of isocereulides L and N (**6, 8**), (**b**) fraction 6 of strain F4810/72/SCV/AN for isolation of isocereulides H and M (**2, 7**), (**c**) fraction 6 of strain F4810/72 for isolation of isocereulides I and J (**3**, **4**), (**d**) fraction 7 of strain F4810/72/SCV/AN for isolation of cereulide (**1**), isocereulides H and M (**2**, **7**), and (**e**) fraction 9 of strain F4810/72 for isolation of cereulide (**1**) and isocereulide K (**5**).

B. Dipeptide synthesis, isolation and characterization *via* 1D- and 2D-NMR data and $MS^e$ fragmentation

Adequate dipeptide references were synthesized as reported recently and considering literature protocol.[1,2,4,5] In brief, the respective enantiomeric pure wang resin bound amino acid (Fmoc-L-2-Abu, Fmoc-D-Ala, Fmoc-Gly and Fmoc-L-Val; 0.1 mmol each), was steeped in DMF for 30 minutes, washed (3×5 mL DMF), incubated with piperidine (3×3 mL, 20 % in DMF, 2 min), and washed again (3×5 mL DMF). Separately, *N,N*-Diisopropylethylamine (1.0 mmol) was spiked to *O*-(benzotriazol-1-yl)-*N,N,N',N'*-tetramethyluronium hexafluorophosphate (0.49 mmol) and the respective *α*-hydroxy acid D-*α*-hydroxypropanoic acid (D-*O*-Ala), L-2-hydroxypropanoic acid (L-*O*-Ala), L-*α*-hydroxyisovaleric acid (L-*O*-Val), D-*α*-hydroxyisocaproic acid (D-*O*-Leu), L-*α*-hydroxyisocaproic acid (L-*O*-Leu), (2*R*,3*R*)-2-hydroxy-3-methylpentanoic acid (D-*O*-Ile), and (2*S*,3*S*)-2-hydroxy-3-methylpentanoic acid (L-*O*-Ile; 0.5 mmol; in 1 mL DMF), respectively. Under nitrogen atmosphere, the *α*-hydroxy acid solution was incubated with the prepared wang resin (RT, 1 h. The resin was washed with dichloromethane and methanol (5×3 mL each) and the resin bound dipeptide released under nitrogen atmosphere by applying a mixture of trifluoroacetic acid and water (95/5, *v/v*, 5 mL) and stirring overnight. The mixture was filtrated and the resin residue washed with trifluoroacetic acid and water (95/5, *v/v*, 2×2 mL) and water (2×2 mL). The liquids were unified, excess water and acid were evaporated and the samples were freeze dried twice.

Purification of the dipeptides was obtained with semi-preparative HPLC, consisting of two PU-2087 pumps (Jasco, Groß-Umstadt, Germany), DG-2080-53 degaser (Jasco, Groß-Umstadt, Germany), and UV 2075 detector (Jasco, Groß-Umstadt, Germany). The sample was manually injected using a Rh 7725i loop injection valve (Rheodyne, Bensheim, Germany), data evaluation was performed with Chrompass 1.8.6.1 (Jasco, Groß-Umstadt, Germany). The column was kept at room temperature with 0.1% aqueous HCOOH (Solvent A) and MeCN

(0.1% HCOOH; Solvent B) at a flow of 4 mL/min. The effluent was monitored at 220 nm. The specific parameters for the dipeptides D-*O*-Ala-L-Val, L-*O*-Ala-L-Val, L-*O*-Ile-Gly, L-*O*-Leu-Gly, D-*O*-Ile-L-Val and L-*O*-Ile-L-Val are as follows:

Stationary phase: 250×10 mm, Synergi 4u Polar-RP 80Å (Phenomenex, Aschaffenburg, Germany)

Gradient: D-*O*-Ala-L-Val, L-*O*-Ala-L-Val: 10% B isocratically for 3 min, increase in 2 min to 20% B, hold for 8 min, increase in 2 min to 100% B, hold for 2 min, decrease within 2 min to 10% B, followed by 3 min equilibration time.

L-*O*-Ile-Gly, L-*O*-Leu-Gly: 10% B isocratically for 3 min, increase in 7 min to 30% B, in 5 min to 100% B, hold for 2 min, and decrease within 1 min to 10% B, followed by 3 min equilibration time.

D-*O*-Ile-L-Val, L-*O*-Ile-L-Val: 30% B isocratically for 3 min, increase in 7 min to 70% B, in 2 min to 100% B, hold for 2 min, decrease within 1 min to 30% B, followed by 3 min equilibration time.

The specific parameters for the dipeptides D-*O*-Ile-D-Ala, L-*O*-Ile-D-Ala, D-*O*-Ile-D-Ser, L-*O*-Ile-D-Ser, D-*O*-Val-L-2-Abu and L-*O*-Val-L-2-Abu are enlisted below:

Stationary phase: VP 250/10 Nucleodur 100-5 C18ec (Macherey-Nagel GmbH & Co. KG, Düren, Germany)

Gradient: D-*O*-Ile-D-Ala, L-*O*-Ile-D-Ala, D-*O*-Ile-D-Ser, L-*O*-Ile-D-Ser, D-*O*-Val- L-2-Abu, L-*O*-Val-L-2-Abu: 10% B for 3 min isocratically, increase in 2 min to 20% B, hold for 8 min, increase in 2 min to 100% B, hold for 2 min, decrease within 2 min to 10% B, followed by 3 min equilibration time.

The solvent of the purified dipeptides (D-*O*-Ala-L-Val, L-*O*-Ala-L-Val, L-*O*-Ile-Gly, L-*O*-Leu-Gly, D-*O*-Ile-D-Ala, L-*O*-Ile-D-Ala, D-*O*-Ile-D-Ser, L-*O*-Ile-D-Ser, D-*O*-Ile-L-Val, L-*O*-Ile-L-Val, D-*O*-Val-L-2-Abu, L-*O*-Val-L-2-Abu) was evaporated under nitrogen current and the substance suspended in water (5 mL). After freeze drying, molecular characterization and structure determination were performed by applying 1D/2D-NMR-spectroscopy and UPLC-TOF-$MS^e$ experiments. The dipeptides D-*O*-Leu-D-Ala, L-*O*-Leu-D-Ala, D-*O*-Leu-D-Ser, D-*O*-Leu-L-Ser, D-*O*-Leu-L-Val, L-*O*-Leu-L-Val and L-*O*-Val-L-Val were obtained by Marxen *et al.* 2015.[1] The chemical structures and NMR-data of all dipeptides are enlisted below, the numeration of single atoms is corresponding to **Figure S3**. NMR-data for D-*O*-Leu-D-Ala, L-*O*-Val-L-Val, L-*O*-Leu-L-Val, D-*O*-Leu-L-Val, L-*O*-Ile-L-Val, D-*O*-Ile-L-Val are according to literature.[1,2] $MS^e$ fragmentation patterns of dipeptides present in cereulide (**1**) and isocereulides H–N (**2–8**) are pictured in **Figure S4.1** and **S4.2**.

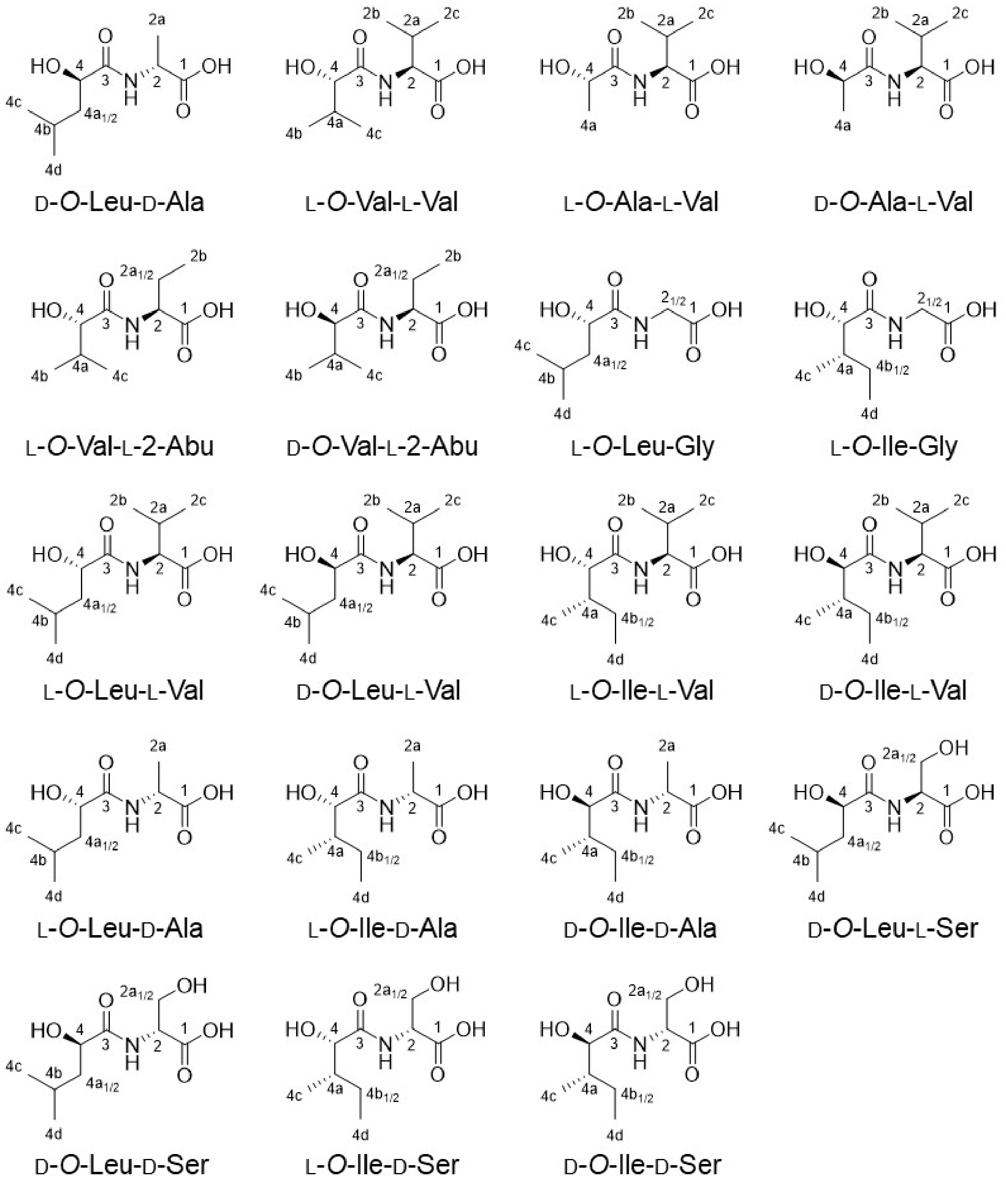


**Figure S3.** Structures of synthesized reference dipeptide units, with numbered atoms for assignment of NMR data.

**D-*O*-Leu-D-Ala:** $^1$H-NMR [500 MHz, $d_3$-MeOD, COSY, 298 K]: δ 0.95 [d, 6H, $J$ = 6.7 Hz, $H_3$-C(4c, d)], 1.41 [d, 3H, $J$ = 7.3 Hz, $H_3$-C(2a)], 1.44–1.59 [m, 2H, $H_2$-C(4a)], 1.81–1.91 [m, 1H, H-C(4b)], 4.05 [dd, 1H, $J$ = 3.7, 9.6 Hz, H-C(4)], 4.38 [q, 1H, $J$ = 7.2 Hz, H-C(2)]. $^{13}$C-NMR [125 MHz, $d_3$-MeOD, HSQC, HMBC, 298 K]: δ 18.5 [C(2a)], 21.9 [C(4c)], 24.1 [C(4d)], 25.7 [C(4b)], 44.9 [C(4a)], 49.3 [C(2)], 71.4 [C(4)], 176.6 [C(1)], 177.6 [C(3)].

**L-*O*-Val-L-Val:** $^1$H-NMR [400 MHz, $d_3$-MeOD, COSY, 298 K]: δ 0.87 [d, 3H, $J$ = 6.8 Hz, $H_3$-C(4b)], 0.96 [d, 3H, $J$ = 7.1 Hz, $H_3$-C(2b)], 0.98 [d, 3H, $J$ = 7.1 Hz, $H_3$-C(2c)], 1.02 [d, 3H, $J$ = 7.0 Hz, $H_3$-C(4c)], 2.05–2.16 [m, 1H, H-C(4a)], 2.16–2.28 [m, 1H, H-C(2a)], 3.89 [d, 1H, $J$ = 3.4 Hz, H-C(4)], 4.36 [d, 1H, $J$ = 4.7 Hz, H-C(2)]. $^{13}$C-NMR [100 MHz, $d_3$-MeOD, HSQC, HMBC, 298 K]: δ 16.4 [C(4b)], 18.2 [C(2b)], 19.8 [2C, C(2c, 4c)], 32.3 [C(2a)], 33.0 [C(4a)], 58.6 [C(2)], 77.2 [C(4)], 175.1 [C(1)], 176.6 [C(3)].

**L-*O*-Ala-L-Val:** $^1$H-NMR [500 MHz, $d_3$-MeOD, COSY, 298 K]: δ 0.95 [d, 3H, $J$ = 6.8 Hz, $H_3$-C(2b)], 0.97 [d, 3H, $J$ = 6.8 Hz, $H_3$-C(2c)], 1.36 [d, 3H, $J$ = 6.7 Hz, $H_3$-C(4a)], 2.17–2.25 [m, 1H, H-C(2a)], 4.15 [q, 1H, $J$ = 6.8 Hz, H-C(4)], 4.35 [d, 1H, $J$ = 5.0 Hz, H-C(2)]. $^{13}$C-NMR [125 MHz, $d_3$-MeOD, HSQC, HMBC, 298 K]: δ 18.1 [C(2b)], 19.7 [C(2c)], 21.6 [C(4a)], 32.4 [C(2a)], 58.5 [C(2)], 69.3 [C(4)], 175.0 [C(1)], 177.8 [C(3)].

**D-*O*-Ala-L-Val:** $^1$H-NMR [500 MHz, $d_3$-MeOD, COSY, 298 K]: δ 0.95 [d, 3H, $J$ = 6.9 Hz, $H_3$-C(2b)], 0.97 [d, 3H, $J$ = 6.9 Hz, $H_3$-C(2c)], 1.35 [d, 3H, $J$ = 6.8 Hz, $H_3$-C(4a)], 2.11–2.27 [m, 1H, H-C(2a)], 4.17 [q, 1H, $J$ = 6.9 Hz, H-C(4)], 4.33 [d, 1H, $J$ = 5.0 Hz, H-C(2)]. $^{13}$C-NMR [125 MHz, $d_3$-MeOD, HSQC, HMBC, 298 K]: δ 18.2 [C(2b)], 19.7 [C(2c)], 21.2 [C(4a)], 32.3 [C(2a)], 58.7 [C(2)], 69.1 [C(4)], 175.0 [C(1)], 177.7 [C(3)].

**L-*O*-Val-L-2-Abu:** $^1$H-NMR [500 MHz, $d_3$-MeOD, COSY, 298 K]: δ 0.84 [d, 3H, $J$ = 6.9 Hz, $H_3$-C(4c)], 0.92 [t, 3H, $J$ = 7.5 Hz, $H_3$-C(2b)], 0.99 [d, 3H, $J$ = 6.9 Hz, $H_3$-C(4b)], 1.70–1.84 [m, 1H, H-C($2a_1$)], 1.86–1.98 [m, 1H, H-C($2a_2$)], 2.03–2.12 [m, 1H, H-C(4a)], 3.86

[d, 1H, $J$ = 3.5 Hz, H-C(4)], 4.36 [dd, 1H, $J$ = 7.3, 5.1 Hz, H-C(2)]. $^{13}$C-NMR [125 MHz, $d_3$-MeOD, HMBC, HSQC, 298 K]: δ 10.2 [C(2b)], 16.2 [C(4c)], 19.6 [C(4b)], 26.2 [C(2a)], 32.9 [C(4a)], 54.5 [C(2)], 77.0 [C(4)], 175.3 [C(1)], 176.4 [C(3)].

**D-*O*-Val-L-2-Abu:** $^{1}$H-NMR [500 MHz, $d_3$-MeOD, COSY, 298 K]: δ 0.87 [d, 3H, $J$ = 6.8 Hz, $H_3$-C(4c)], 0.96 [t, 3H, $J$ = 7.5 Hz, $H_3$-C(2b)], 1.01 [d, 3H, $J$ = 6.9 Hz, $H_3$-C(4b)], 1.72–1.83 [m, 1H, H-C(2$a_1$)], 1.88–1.98 [m, 1H, H-C(2$a_2$)], 2.03–2.13 [m, 1H, H-C(4a)], 3.89 [d, 1H, $J$ = 3.7 Hz, H-C(4)], 4.36 [dd, 1H, $J$ = 7.7, 5.2 Hz, H-C(2)]. $^{13}$C-NMR [125 MHz, $d_3$-MeOD, HMBC, HSQC, 298 K]: δ 10.4 [C(2b)], 16.3 [C(4c)], 19.8 [C(4b)], 26.2 [C(2a)], 33.2 [C(4a)], 54.6 [C(2)], 77.2 [C(4)], 175.2 [C(1)], 176.8 [C(3)].

**L-*O*-Leu-Gly:** $^{1}$H-NMR [400 MHz, $d_3$-MeOD, COSY, 298 K]: δ 0.95 [d, 6H, $J$ = 6.7 Hz, $H_3$-C(4c, 4d)], 1.48–1.59 [m, 2H, $H_2$-C(4$a_{1/2}$)], 1.83–1.92 [m, 1H, H-C(4b)], 3.93 [dd, 2H, $J$ = 26.9, 17.8 Hz, $H_2$-C(2$_{1/2}$)], 4.08 [dd, 1H, $J$ = 9.4, 4.0 Hz, H-C(4)]. $^{13}$C-NMR [100 MHz, $d_3$-MeOD, HSQC, 298 K]: δ 21.8 [C(4c/4d)], 24.1 [C(4c/4d)], 25.7 [C(4b)], 41.8 [C(2)], 44.8 [C(4a)], 71.5 [C(4)], 173.4 [C(1)], 178.6 [C(3)].

**L-*O*-Ile-Gly:** $^{1}$H-NMR [400 MHz, $d_3$-MeOD, COSY, 298 K]: δ 0.89 [t, 3H, $J$ = 7.2 Hz, $H_3$-C(4d)], 0.99 [d, 3H, $J$ = 6.9 Hz, $H_3$-C(4c)], 1.16–1.26 [m, 1H, H-C(4$a_1$)], 1.43–1.53 [m, 1H, H-C(4b)], 1.79–1.88 [m, 1H, H-C(4$a_2$)], 3.91 [dd, 2H, $J$ = 34.6, 17.9 Hz, $H_2$-C(2$_{1/2}$)], 3.93 [d, 1H, $J$ = 3.9 Hz, H-C(4)]. $^{13}$C-NMR [100 MHz, $d_3$-MeOD, HSQC, 298 K]: δ 12.4 [C(4d)], 16.2 [C(4c)], 24.5 [C(4b)], 40.0 [C(4a)], 42.2 [C(2)], 77.2 [C(4)], 173.8 [C(1)], 177.2 [C(3)].

**L-*O*-Leu-L-Val:** $^{1}$H-NMR [500 MHz, $d_3$-MeOD, COSY, 298 K]: δ 0.96 [m, 12H, $H_3$-C(2b, 2c, 4c, 4d)], 1.53 [m, 2H, $H_2$-C(4a)], 1.87 [m, 1H, H-C(4b)], 2.20 [m, 1H, H-C(2a)], 4.07 [dd, 1H, $J$ = 9.7, 3.5 Hz, H-C(4)], 4.34 [d, 1H, $J$ = 5.0 Hz, H-C(2)]. $^{13}$C-NMR [125 MHz, $d_3$-MeOD, HMBC, HSQC, 298 K]: δ 18.0 [C(2b/2c)], 19.6 [C(2b/2c)], 21.7 [C(4c/4d)], 24.0 [C(4c/4d)], 25.6 [C(4b)], 32.3 [C(2a)], 45.0 [C(4a)], 58.5 [C(2)], 71.5 [C(4)], 175.2 [C(1)], 177.6 [C(3)].

**D-*O*-Leu-L-Val:** $^{1}$H-NMR [500 MHz, *d$_3$*-MeOD, COSY, 298 K]: δ 0.96 [m, 12H, $H_3$-C(2b, 2c, 4c, 4d)], 1.53 [m, 2H, $H_2$-C(4a)], 1.86 [m, 1H, H-C(4b)], 2.21 [m, 1H, H-C(2a)], 4.10 [dd, 1H, *J* = 4.3, 8.9 Hz, H-C(4)], 4.34 [d, 1H, *J* = 5.1 Hz, H-C(2)]. $^{13}$C-NMR [125 MHz, *d$_3$*-MeOD, HMBC, HSQC, 298 K]: δ 18.1 [C(2b/2c)], 19.5 [C(2b/2c)], 21.8 [C(4c/4d)], 23.9 [C(4c/4d)], 25.5 [C(4b)], 32.1 [C(2a)], 44.6 [C(4a)], 58.4 [C(2)], 71.4 [C(4)], 174.6 [C(1)], 177.7 [C(3)].

**L-*O*-Ile-L-Val:** $^{1}$H-NMR [500 MHz, *d$_3$*-MeOD, COSY, 298 K]: δ 0.83 [t, 3H, *J* = 7.4 Hz, $H_3$-C(4d)], 0.87 [d, 3H, *J* = 6.8 Hz, $H_3$-C(2b)], 0.89 [d, 3H, *J* = 6.8 Hz, $H_3$-C(2c)], 0.93 [d, 3H, *J* = 6.9 Hz, $H_3$-C(4c)], 1.17–1.32 [m, 1H, H-C($4b_1$)], 1.39–1.52 [m, 1H, H-C($4b_2$)], 1.79–1.90 [m, 1H, H-C(4a)], 2.16–2.27 [m, 1H, H-C(2a)], 3.85 [d, 1H, *J* = 3.6 Hz, H-C(4)], 4.22 [d, 1H, *J* = 3.6 Hz, H-C(2)]. $^{13}$C-NMR [125 MHz, *d$_3$*-MeOD, HSQC, HMBC, 298 K]: δ 12.4 [C(4d)], 16.3 [C(4c)], 18.4 [C(2b)], 20.2 [C(2c)], 24.7 [C(4b)], 32.7 [C(2a)], 39.9 [C(4a)], 60.0 [C(2)], 77.4 [C(4)], 176.4 [C(3)], 177.1[C(1)].

**D-*O*-Ile-L-Val:** $^{1}$H-NMR [500 MHz, *d$_3$*-MeOD, COSY, 298 K]: δ 0.79 [d, 3H, *J* = 6.9 Hz, $H_3$-C(4b)], 0.88–0.99 [m, 9H, $H_3$-C(2b, 2c, 4d)], 1.25–1.54 [m, 2H, $H_2$-C(4c)], 1.81 [hd, 1H, *J* = 7.0, 2.8 Hz, H-C(4a)], 2.19 [hd, 1H, *J* = 6.9, 5.1 Hz, H-C(2a)], 4.04 [d, 1H, *J* = 2.8 Hz, H-C(4)], 4.32 [d, 1H, *J* = 5.1 Hz, H-C(2)]. $^{13}$C-NMR [125 MHz, *d$_3$*-MeOD, HMBC, HSQC, 298 K]: δ 12.2 [C(4d)], 13.4 [C(4b)], 18.1 [C(2b)], 19.6 [C(2c)], 27.4 [C(4c)], 32.2 [C(2a)], 39.6 [C(4a)], 58.5 [C(2)], 74.7 [C(4)], 174.9 [C(1)], 177.1 [C(3)].

**L-*O*-Leu-D-Ala:** $^{1}$H-NMR [400 MHz, *d$_3$*-MeOD, COSY, 298 K]: δ 0.95 [d, 6H, *J* = 6.6 Hz, $H_3$-C(4c, 4d)], 1.41 [d, 3H, *J* = 7.1 Hz, $H_3$-C(2a)], 1.49–1.55 [m, 2H, $H_2$-C(4a)], 1.79–1.92 [m, 1H, H-C(4b)], 4.05 [dd, 1H, *J* = 4.8, 8.8 Hz, H-C(4)], 4.37 [q, 1H, *J* = 7.2 Hz, H-C(2)]. $^{13}$C-NMR [100 MHz, *d$_3$*-MeOD, HSQC, HMBC, 298 K]: δ 18.4 [C(2a)], 21.9 [C(4c)], 24.1 [C(4d)], 25.6 [C(4b)], 44.7 [C(4a)], 48.9 [C(2)], 71.5 [C(4)], 176.4 [C(1)], 177.5 [C(3)].

**D-*O*-Ile-D-Ala:** $^{1}$H-NMR [500 MHz, *d$_3$*-MeOD, COSY, 298 K]: δ 0.85 [d, 3H, *J* = 6.8 Hz, $H_3$-C(4d)], 0.98 [d, 3H, *J* = 6.9 Hz, $H_3$-C(4c)], 1.27–1.41 [m, 1H, H-C($4b_1$)], 1.45 [d, 3H,

$J$ = 7.2 Hz, $H_3$-C(2a)], 1.47–1.59 [m, 1H, H-C($4b_2$)], 1.80–1.93 [m, 1H, H-C(4a)], 4.05 [d, 1H, $J$ = 1.8 Hz, H-C(4)], 4.48 [q, 1H, $J$ = 7.2 Hz, H-C(2)]. $^{13}$C-NMR [125 MHz, $d_3$-MeOD, HSQC, HMBC, 298 K]: δ 12.3 [C(4d)], 13.5 [C(4c)], 18.4 [C(2a)], 27.4 [C(4b)], 39.7 [C(4a)], 48.6 [C(2)], 74.7 [C(4)], 175.8 [C(1)], 176.9 [C(3)].

**L-*O*-Ile-D-Ala:** $^1$H-NMR [500 MHz, $d_3$-MeOD, COSY, 298 K]: δ 0.88 [d, 3H, $J$ = 7.0 Hz, $H_3$-C(4c)], 0.98 [d, 3H, $J$ = 7.0 Hz, $H_3$-C(4c)], 1.13–1.27 [m, 1H, H-C($4b_1$)], 1.42 [d, 3H, $J$ = 6.4 Hz, $H_3$-C(2a)], 1.45–1.56 [m, 1H, H-C($4b_2$)], 1.77–1.89 [m, 1H, H-C(4a)], 3.90 [d, 1H, $J$ = 1.9 Hz, H-C(4)], 4.41 [q, 1H, $J$ = 7.0 Hz, H-C(2)]. $^{13}$C-NMR [125 MHz, $d_3$-MeOD, HSQC, HMBC, 298 K]: δ 12.3 [C(4c)], 16.1 [C(4d)], 18.2 [C(2a)], 24.5 [C(4b)], 40.1 [C(4a)], 48.7 [C(2)], 77.1 [C(4)], 175.9 [C(1)], 176.5 [C(3)].

**D-*O*-Ile-D-Ser:** $^1$H-NMR [400 MHz, $d_3$-MeOD, COSY, 298 K]: δ 0.89 [t, 3H, $J$ = 7.5 Hz, $H_3$-C(4c)], 1.00 [d, 3H, $J$ = 7.1 Hz, $H_3$-C(4d)], 1.14–1.30 [m, 1H, H-C($4b_1$)], 1.44–1.58 [m, 1H, H-C($4b_2$)], 1.79–1.91 [m, 1H, H-C(4a)], 3.83 [dd, 1H, $J$ = 3.7, 11.4 Hz, H-C($2a_1$)], 3.95 [d, 1H, $J$ = 3.1 Hz, H-C(4)], 3.97 [dd, 1H, $J$ = 4.3, 11.2 Hz, H-C($2a_2$)], 4.48 [t, 1H, $J$ = 4.0 Hz, H-C(2)]. $^{13}$C-NMR [100 MHz, $d_3$-MeOD, HSQC, HMBC, 298 K]: δ 12.4 [C(4c)], 16.2 [C(4d)], 24.4 [C(4b)], 40.1 [C(4a)], 55.6 [C(2)], 63.1 [C(2a)], 77.2 [C(4)], 173.4 [C(1)], 176.8 [C(3)].

**L-*O*-Ile-D-Ser:** $^1$H-NMR [400 MHz, $d_3$-MeOD, COSY, 298 K]: δ 0.83 [d, 3H, $J$ = 6.6 Hz, $H_3$-C(4d)], 0.95 [t, 3H, $J$ = 7.1 Hz, $H_3$-C(4c)], 1.26–1.39 [m, 1H, H-C($4b_1$)], 1.44–1.58 [m, 1H, H-C($4b_2$)], 1.79–1.91 [m, 1H, H-C(4a)], 3.80 [dd, 1H, $J$ = 4.0, 11.2 Hz, H-C($2a_1$)], 3.97 [dd, 1H, $J$ = 3.7, 10.9 Hz, H-C($2a_2$)], 4.07 [d, 1H, $J$ = 2.8, 11.2 Hz, H-C(4)], 4.50 [t, 1H, $J$ = 3.8 Hz, H-C(2)]. $^{13}$C-NMR [100 MHz, $d_3$-MeOD, HSQC, HMBC, 298 K]: δ 12.3 [C(4c)], 13.6 [C(4d)], 27.4 [C(4b)], 39.6 [C(4a)], 55.6 [C(2)], 63.2 [C(2a)], 74.9 [C(4)], 173.5 [C(1)], 177.3 [C(3)].

**D-*O*-Leu-D-Ser:** $^1$H-NMR [400 MHz, $d_3$-MeOD, COSY, 298 K]: δ 0.95 [d, 6H, $J$ = 6.7 Hz, $H_3$-C(4c, 4d)], 1.46–1.61 [m, 2H, $H_2$-C(4a)], 1.80–1.94 [m, 1H, H-C(4b)], 3.81 [dd, 1H, $J$ = 3.7, 10.9 Hz, H-C($2a_1$)], 3.97 [dd, 1H, $J$ = 4.1, 11.4 Hz, H-C($2a_2$)], 4.09 [dd, 1H, $J$ = 3.9, 9.3 Hz, H-

C(4)], 4.46 [t, 1H, $J = 3.6$ Hz, H-C(2)]. $^{13}$C-NMR [100 MHz, $d_3$-MeOD, HSQC, HMBC, 298 K]: δ 21.9 [C(4c)], 24.1 [C(4d)], 25.7 [C(4b)], 44.8 [C(4a)], 55.8 [C(2)], 63.2 [C(2a)], 71.5 [C(4)], 173.7 [C(1)], 178.0 [C(3)].

**D-*O*-Leu-L-Ser:** $^{1}$H-NMR [400 MHz, $d_3$-MeOD, COSY, 298 K]: δ 0.95 [d, 6H, $J = 6.6$ Hz, $H_3$-C(4c, 4d)], 1.48–1.62 [m, 2H, $H_2$-C(4a)], 1.80–1.95 [m, 1H, H-C(4b)], 3.84 [dd, 1H, $J = 3.7$, 11.1 Hz, H-C($2a_1$)], 3.96 [dd, 1H, $J = 4.2$, 11.1 Hz, H-C($2a_2$)], 4.09 [dd, 1H, $J = 4.7$, 8.5 Hz, H-C(4)], 4.45 [t, 1H, $J = 3.8$ Hz, H-C(2)]. $^{13}$C-NMR [100 MHz, $d_3$-MeOD, HSQC, HMBC, 298 K]: δ 21.9 [C(4c)], 24.1 [C(4d)], 25.6 [C(4b)], 44.6 [C(4a)], 55.9 [C(2)], 63.1 [C(2a)], 71.6 [C(4)], 173.6 [C(1)], 177.9 [C(3)].

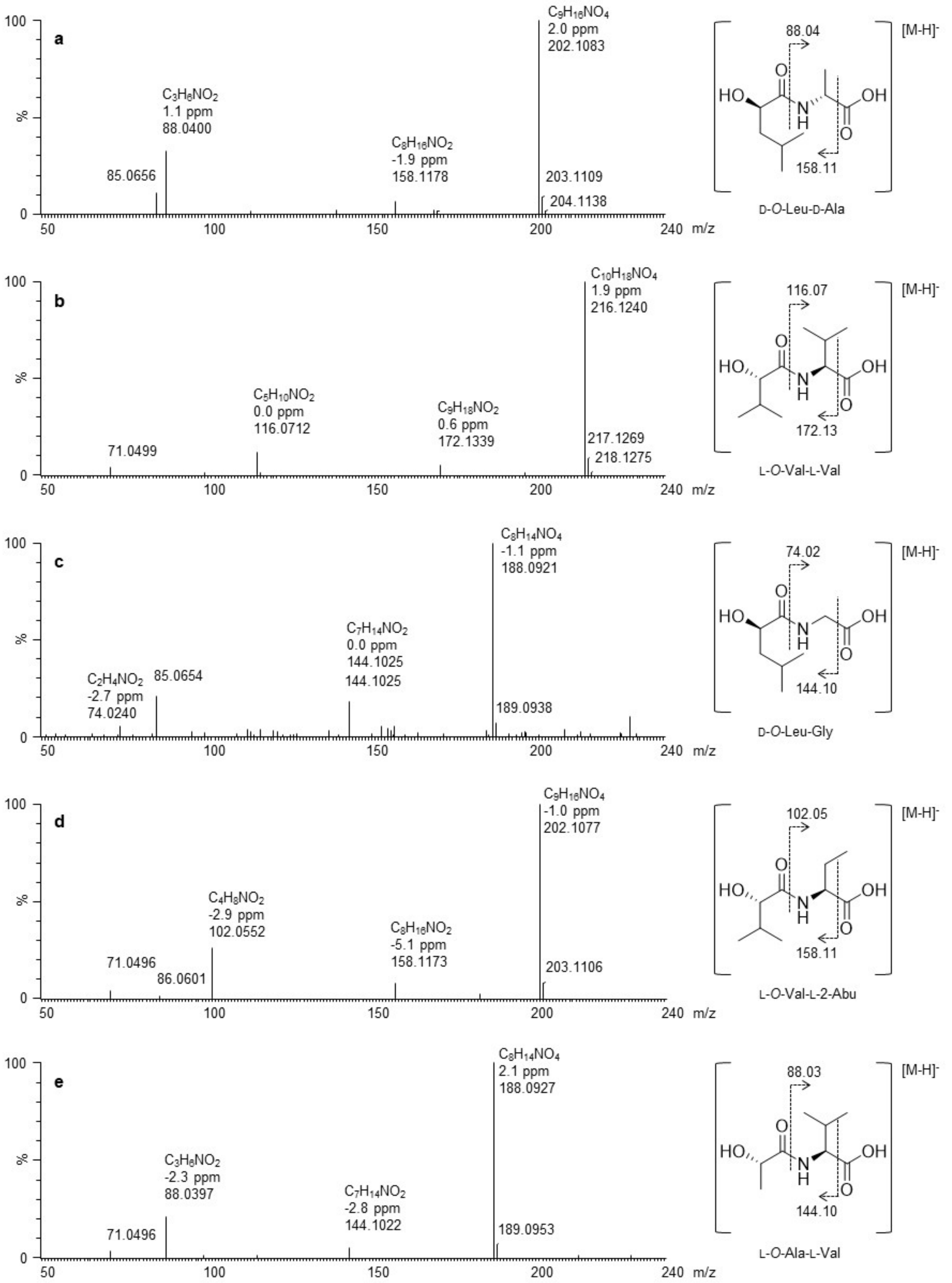


**Figure S4.1.** Mass spectrometric fragmentation pattern (UPLC-ESI-TOF-MS$^e$) of dipeptides (**a**) D-*O*-Leu-D-Ala, and (**b**) L-*O*-Val-L-Val present in cereulide (**1**), (**c**) D-*O*-Leu-Gly present in isocereulide H (**2**) and M (**7**), (**d**) L-*O*-Val-L-2-Abu present in isocereulide I (**3**), and (**e**) L-*O*-Ala-L-Val present in isocereulide J (**4**).

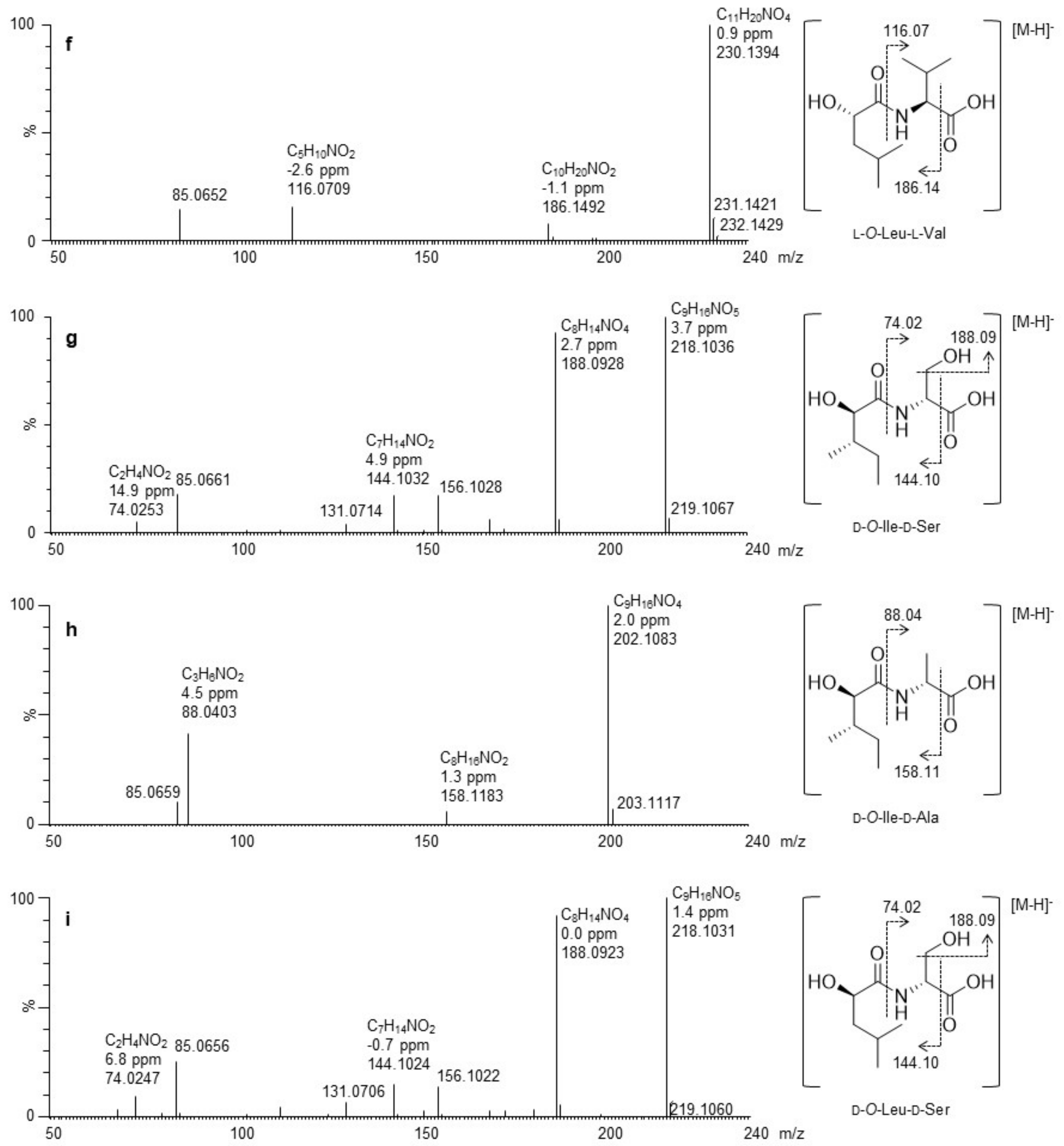


**Figure S4.2.** Mass spectrometric fragmentation pattern (UPLC-ESI-TOF-MS$^{e}$) of dipeptides (**f**) L-*O*-Leu-L-Val present in isocereulide K (**5**), (**g**) D-*O*-Ile-D-Ser present in isocereulide L (**6**), (**h**) D-*O*-Ile-D-Ala present in isocereulide M (**7**) and N (**8**), and (**i**) D-*O*-Leu-D-Ser present in isocereulide N (**8**).

C. Identification of the dipeptide units in the alkaline hydrolysates of isocereulides H–N (**2**–**8**) by co-chromatography

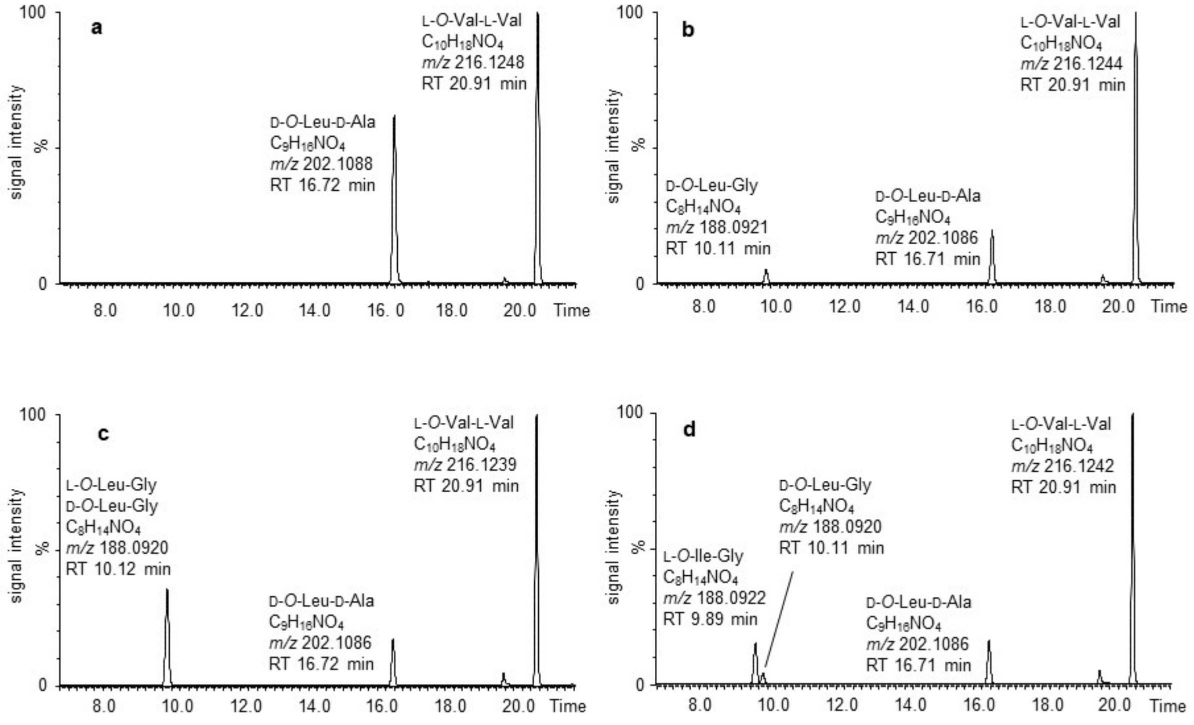


**Figure S5.** UPLC-ESI$^{-}$-TOF-MS-chromatograms of alkaline hydrolysis of (**a**) cereulide (**1**), (**b**) isocereulide H (**2**), (**c**) **2** spiked with L-*O*-Leu-Gly, and (**d**) **2** spiked with L-*O*-Ile-Gly.

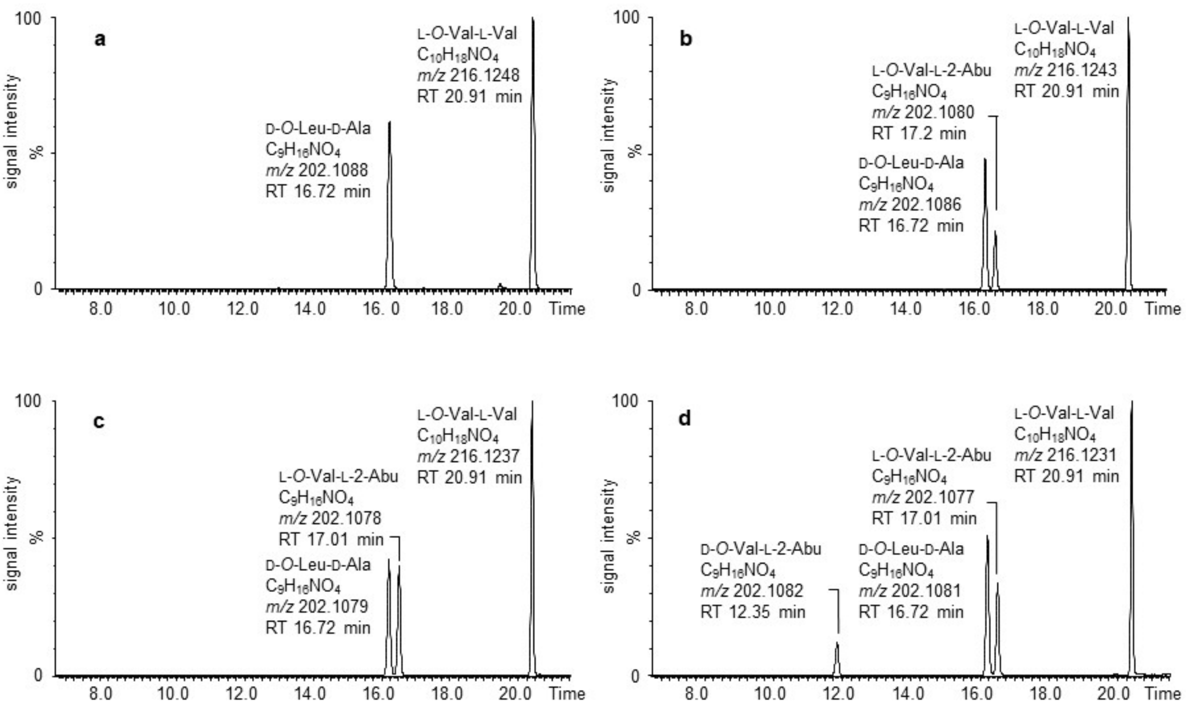


**Figure S6.** UPLC-ESI$^{-}$-TOF-MS-chromatograms of alkaline hydrolysis of (**a**) cereulide (**1**), (**b**) isocereulide I (**3**), (**c**) **3** spiked with L-*O*-Val-L-2-Abu, and (**d**) **3** spiked with D-*O*-Val-L-2-Abu.

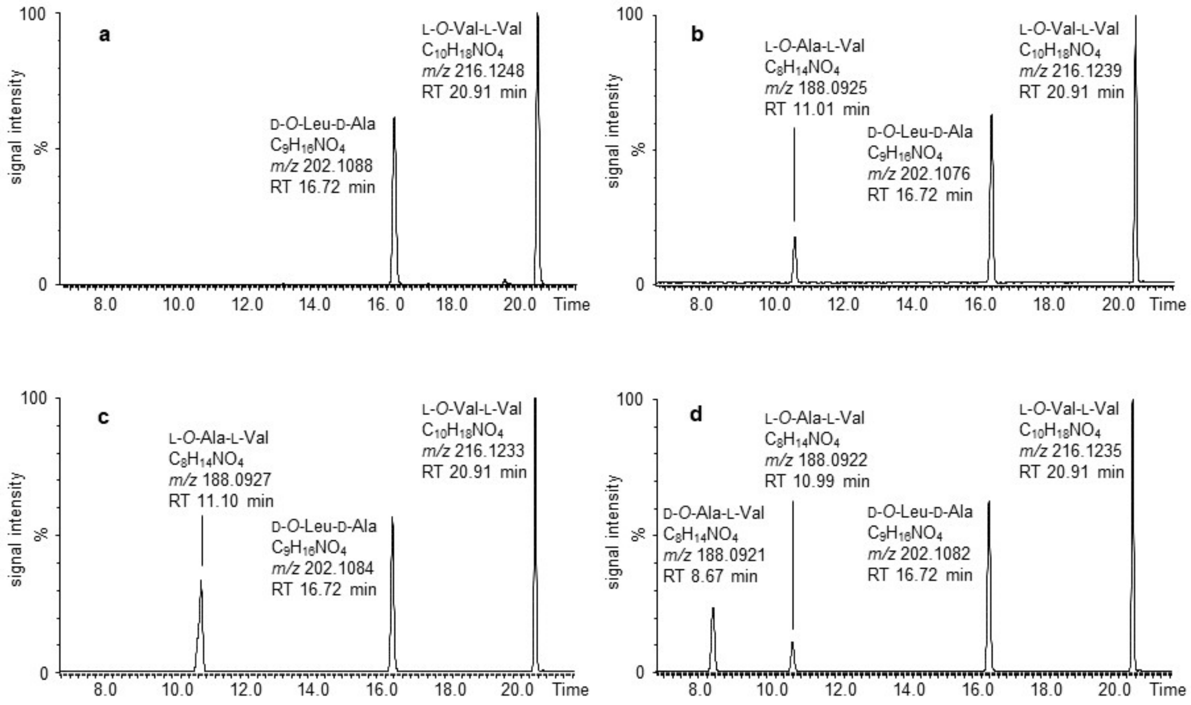


**Figure S7.** UPLC-ESI$^-$-TOF-MS-chromatograms of alkaline hydrolysis of (**a**) cereulide (**1**), (**b**) isocereulide J (**4**), (**c**) **4** spiked with L-*O*-Ala-L-Val, and (**d**) **4** spiked with D-*O*-Ala-L-Val.

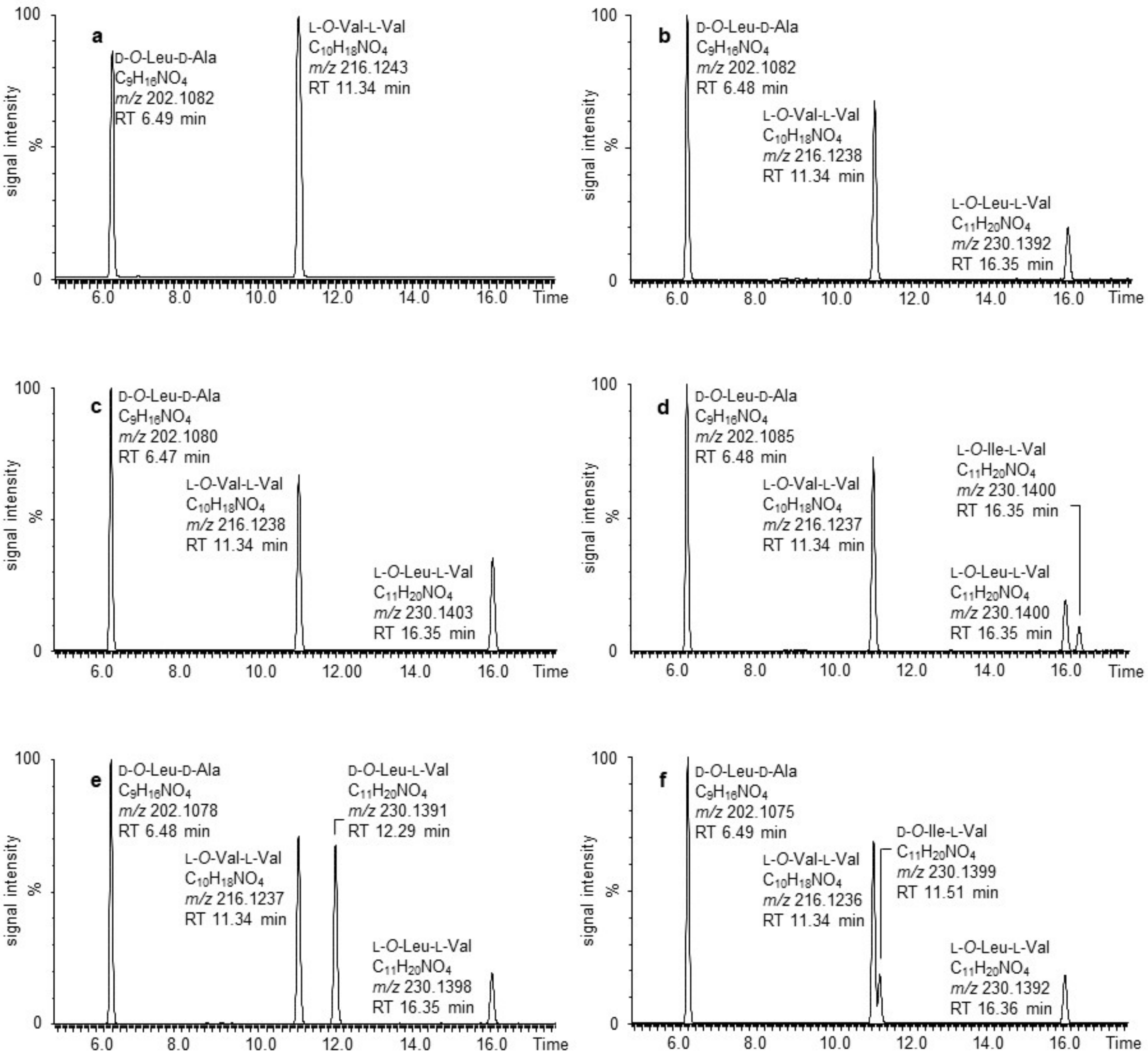


**Figure S8.** UPLC-ESI⁻-TOF-MS-chromatograms of alkaline hydrolysis of (**a**) cereulide (**1**), (**b**) isocereulide K (**5**), (**c**) **5** spiked with L-*O*-Leu-L-Val, (**d**) **5** spiked with L-*O*-Ile-L-Val, (**e**) **5** spiked with D-*O*-Leu-L-Val, and (**f**) **5** spiked with D-*O*-Ile-L-Val.

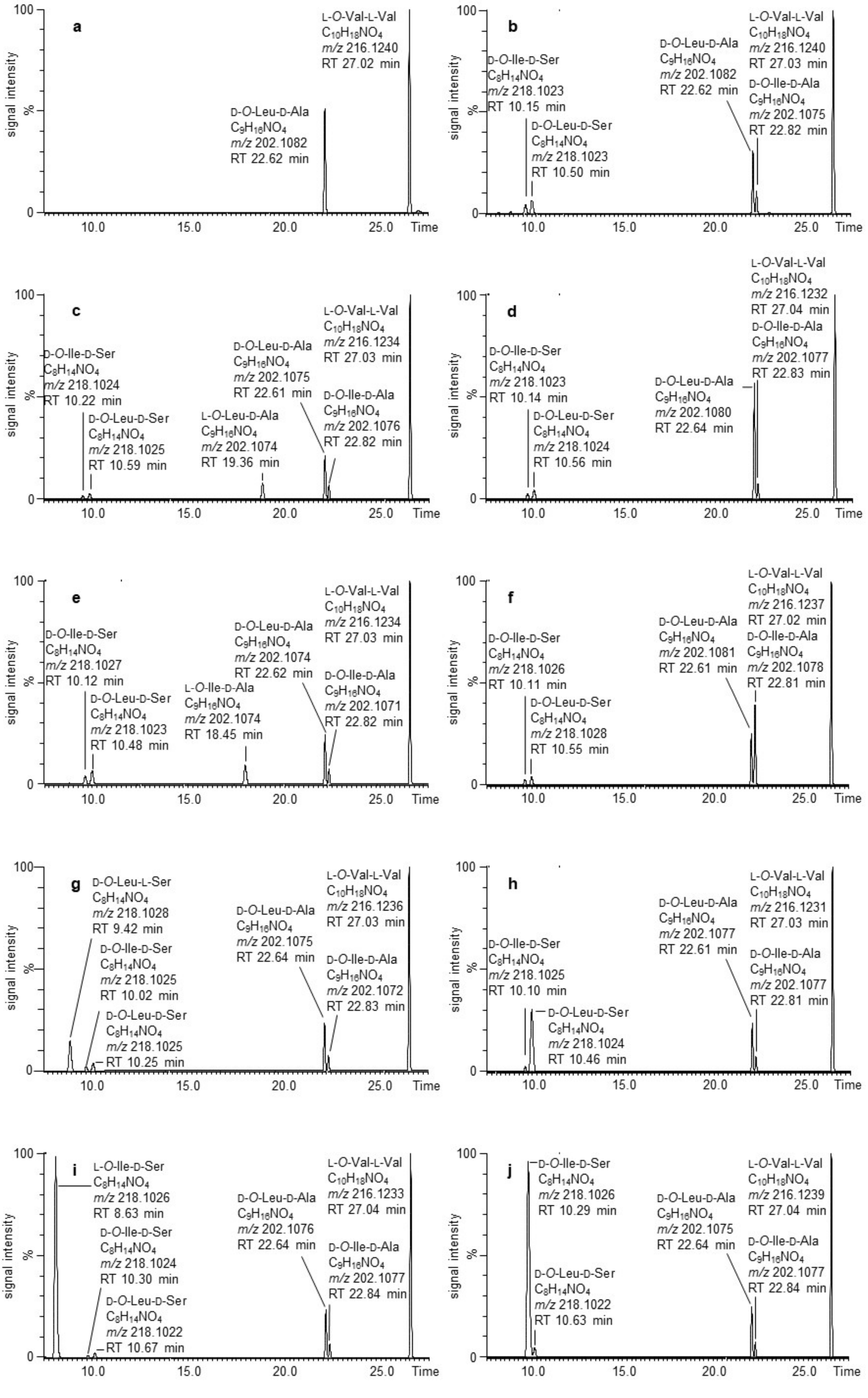

a
signal intensity
100
%
0
10.0
15.0
20.0
25.0
Time
D-O-Leu-D-Ala
$C_9H_{16}NO_4$
m/z 202.1082
RT 22.62 min
L-O-Val-L-Val
$C_{10}H_{18}NO_4$
m/z 216.1240
RT 27.02 min
b
D-O-Ile-D-Ser
$C_8H_{14}NO_4$
m/z 218.1023
RT 10.15 min
D-O-Leu-D-Ser
$C_8H_{14}NO_4$
m/z 218.1023
RT 10.50 min
D-O-Leu-D-Ala
$C_9H_{16}NO_4$
m/z 202.1082
RT 22.62 min
L-O-Val-L-Val
$C_{10}H_{18}NO_4$
m/z 216.1240
RT 27.03 min
D-O-Ile-D-Ala
$C_9H_{16}NO_4$
m/z 202.1075
RT 22.82 min
c
D-O-Ile-D-Ser
$C_8H_{14}NO_4$
m/z 218.1024
RT 10.22 min
D-O-Leu-D-Ser
$C_8H_{14}NO_4$
m/z 218.1025
RT 10.59 min
L-O-Leu-D-Ala
$C_9H_{16}NO_4$
m/z 202.1074
RT 19.36 min
D-O-Leu-D-Ala
$C_9H_{16}NO_4$
m/z 202.1075
RT 22.61 min
L-O-Val-L-Val
$C_{10}H_{18}NO_4$
m/z 216.1234
RT 27.03 min
D-O-Ile-D-Ala
$C_9H_{16}NO_4$
m/z 202.1076
RT 22.82 min
d
D-O-Ile-D-Ser
$C_8H_{14}NO_4$
m/z 218.1023
RT 10.14 min
D-O-Leu-D-Ser
$C_8H_{14}NO_4$
m/z 218.1024
RT 10.56 min
D-O-Leu-D-Ala
$C_9H_{16}NO_4$
m/z 202.1080
RT 22.64 min
L-O-Val-L-Val
$C_{10}H_{18}NO_4$
m/z 216.1232
RT 27.04 min
D-O-Ile-D-Ala
$C_9H_{16}NO_4$
m/z 202.1077
RT 22.83 min
e
D-O-Ile-D-Ser
$C_8H_{14}NO_4$
m/z 218.1027
RT 10.12 min
D-O-Leu-D-Ser
$C_8H_{14}NO_4$
m/z 218.1023
RT 10.48 min
L-O-Ile-D-Ala
$C_9H_{16}NO_4$
m/z 202.1074
RT 18.45 min
D-O-Leu-D-Ala
$C_9H_{16}NO_4$
m/z 202.1074
RT 22.62 min
L-O-Val-L-Val
$C_{10}H_{18}NO_4$
m/z 216.1234
RT 27.03 min
D-O-Ile-D-Ala
$C_9H_{16}NO_4$
m/z 202.1071
RT 22.82 min
f
D-O-Ile-D-Ser
$C_8H_{14}NO_4$
m/z 218.1026
RT 10.11 min
D-O-Leu-D-Ser
$C_8H_{14}NO_4$
m/z 218.1028
RT 10.55 min
D-O-Leu-D-Ala
$C_9H_{16}NO_4$
m/z 202.1081
RT 22.61 min
L-O-Val-L-Val
$C_{10}H_{18}NO_4$
m/z 216.1237
RT 27.02 min
D-O-Ile-D-Ala
$C_9H_{16}NO_4$
m/z 202.1078
RT 22.81 min
g
D-O-Leu-L-Ser
$C_8H_{14}NO_4$
m/z 218.1028
RT 9.42 min
D-O-Ile-D-Ser
$C_8H_{14}NO_4$
m/z 218.1025
RT 10.02 min
D-O-Leu-D-Ser
$C_8H_{14}NO_4$
m/z 218.1025
RT 10.25 min
D-O-Leu-D-Ala
$C_9H_{16}NO_4$
m/z 202.1075
RT 22.64 min
L-O-Val-L-Val
$C_{10}H_{18}NO_4$
m/z 216.1236
RT 27.03 min
D-O-Ile-D-Ala
$C_9H_{16}NO_4$
m/z 202.1072
RT 22.83 min
h
D-O-Ile-D-Ser
$C_8H_{14}NO_4$
m/z 218.1025
RT 10.10 min
D-O-Leu-D-Ser
$C_8H_{14}NO_4$
m/z 218.1024
RT 10.46 min
D-O-Leu-D-Ala
$C_9H_{16}NO_4$
m/z 202.1077
RT 22.61 min
L-O-Val-L-Val
$C_{10}H_{18}NO_4$
m/z 216.1231
RT 27.03 min
D-O-Ile-D-Ala
$C_9H_{16}NO_4$
m/z 202.1077
RT 22.81 min
i
L-O-Ile-D-Ser
$C_8H_{14}NO_4$
m/z 218.1026
RT 8.63 min
D-O-Ile-D-Ser
$C_8H_{14}NO_4$
m/z 218.1024
RT 10.30 min
D-O-Leu-D-Ser
$C_8H_{14}NO_4$
m/z 218.1022
RT 10.67 min
D-O-Leu-D-Ala
$C_9H_{16}NO_4$
m/z 202.1076
RT 22.64 min
L-O-Val-L-Val
$C_{10}H_{18}NO_4$
m/z 216.1233
RT 27.04 min
D-O-Ile-D-Ala
$C_9H_{16}NO_4$
m/z 202.1077
RT 22.84 min
j
D-O-Ile-D-Ser
$C_8H_{14}NO_4$
m/z 218.1026
RT 10.29 min
D-O-Leu-D-Ser
$C_8H_{14}NO_4$
m/z 218.1022
RT 10.63 min
D-O-Leu-D-Ala
$C_9H_{16}NO_4$
m/z 202.1075
RT 22.64 min
L-O-Val-L-Val
$C_{10}H_{18}NO_4$
m/z 216.1239
RT 27.04 min
D-O-Ile-D-Ala
$C_9H_{16}NO_4$
m/z 202.1077
RT 22.84 min

**Figure S9.** UPLC-ESI$^{-}$-TOF-MS-chromatograms of alkaline hydrolysis of (**a**) cereulide (**1**), (**b**) isocereulide L/N (**6+8**), (**c**) **6+8** spiked with L-*O*-Leu-D-Ala, (**d**) **6+8** spiked with D-*O*-Leu-D-Ala, (**e**) **6+8** spiked with L-*O*-Ile-D-Ala, (**f**) **6+8** spiked with D-*O*-Ile-D-Ala, (**g**) **6+8** spiked with D-*O*-Leu-L-Ser, (**h**) **6+8** spiked with D-*O*-Leu-D-Ser, (**i**) **6+8** spiked with L-*O*-Ile-D-Ser, (**j**) **6+8** spiked with D-*O*-Ile-D-Ser.

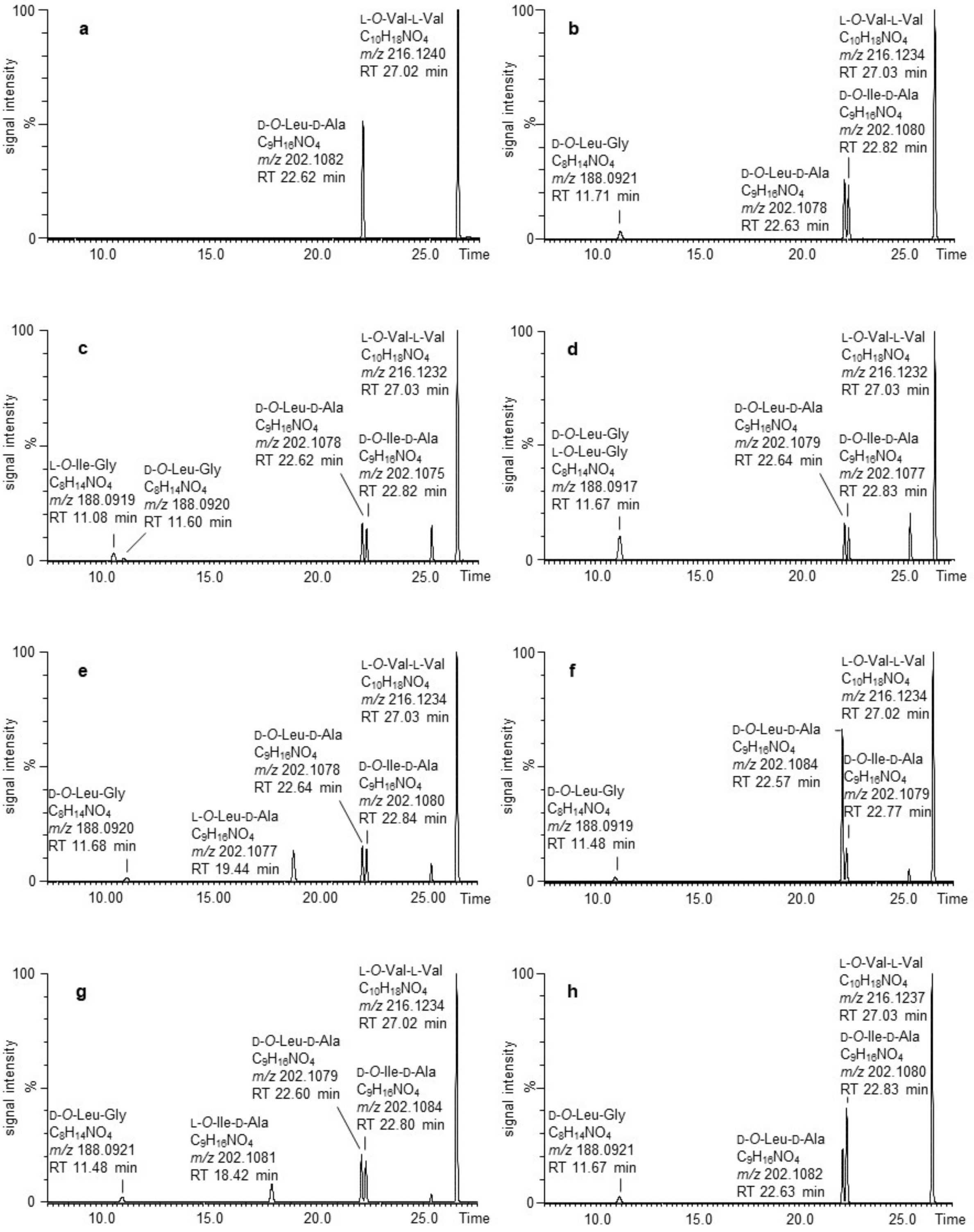


**Figure S10.** UPLC-ESI$^-$-TOF-MS-chromatograms of alkaline hydrolysis of (**a**) cereulide (**1**), (**b**) isocereulide M (**7**), (**c**) **7** spiked with L-*O*-Ile-Gly, (**d**) **7** spiked with L-*O*-Leu-Gly, (**e**) **7** spiked with L-*O*-Leu-D-Ala, (**f**) **7** spiked with D-*O*-Leu-D-Ala, (**g**) **7** spiked with L-*O*-Ile-D-Ala, and (**h**) **7** spiked with D-*O*-Ile-D-Ala.

**Table S1.** Ratios of D/L-amino and D/L- $\alpha$-hydroxy acids in cereulide (**1**) and isocereulides H–N (**2–8**) from UPLC-ESI$^{-}$-TOF-MS data gained after acidic hydrolysis of alkaline hydrolysates.

| Variant[a] | D/L-amino acid ratio[b] | | | | | D/L-$\alpha$-hydroxy acid ratio[c] | | | Additional dipeptide |
|---|---|---|---|---|---|---|---|---|---|
| | D/L-Ser | Gly | D/L-Ala | D/L-2-Abu | D/L-Val | D/L-*O*-Ala | D/L-*O*-Val | D-*O*-Leu + D-*O*-Ile / L-*O*-Leu / L-*O*-Ile[d] | Sequence |
| Cereulide (**1**) | | | 99.9/0.1 | | 0.1/99.9 | | 0.1/99.9 | 99.8/0.1/0.1 | |
| Isocereulide H (**2**) | | 100 | 99.8/0.2 | | 0.2/99.8 | | 0.1/99.9 | 99.8/0.1/0.1 | D-*O*-Leu-Gly |
| Isocereulide I (**3**) | | | 99.9/0.1 | 0.1/99.9 | 0.1/99.9 | | 0.1/99.9 | 99.8/0.1/0.1 | L-*O*-Val-L-2-Abu |
| Isocereulide J (**4**) | | | 99.8/0.2 | | 0.1/99.9 | 0.1/99.9 | 0.1/99.9 | 99.8/0.1/0.1 | L-*O*-Ala-L-Val |
| Isocereulide K (**5**) | | | 99.8/0.2 | | 0.1/99.9 | | 0.1/99.9 | 71.9/28.0/0.1 | L-*O*-Leu-L-Val |
| Isocereulide L[g] (**6**) | 99.9/0.1 | | 99.9/0.1 | | 0.1/99.9 | | | 99.8/0.1/0.1 | D-*O*-Ile-D-Ser |
| Isocereulide M (**7**) | | 100 | 99.9/0.1 | | 0.1/99.9 | | | 99.8/0.1/0.1 | D-*O*-Leu-Gly |
| | | | | | | | | | D-*O*-Ile-D-Ala |
| Isocereulide N[g] (**8**) | 99.9/0.1 | | 99.9/0.1 | | 0.1/99.9 | | | 99.8/0.1/0.1 | D-*O*-Leu-D-Ser |
| | | | | | | | | | D-*O*-Ile-D-Ala |

[a] Determined structures of cereulide (**1**) and isocereulides H–N (**2–8**) are given in **Figure 2a;** isocereulides L and N were isolated as a mixture (approx. 42/58).

[b] Ratio of D- and L-amino acids detected after alkaline hydrolysis followed by acidic hydrolysis of the original dodecadepsipeptide structures of isocereulides

[c] Ratio of D- and L-$\alpha$-hydroxy acids detected after alkaline hydrolysis followed by acidic hydrolysis of the original dodecadepsipeptide structures of isocereulides

[d] D-*O*-Leu and D-*O*-Ile detected together. Ratio of D-*O*-Leu + D-O-Ile / D -*O*-Leu / L-*O*-Ile given. For **2**, **6–8** D-*O*-Leu and D-*O*-Ile moieties were differentiated *via* synthesized dipeptide units and co-chromatography with the alkaline hydrolysates.

D. Enantioselective amino acid and $\alpha$-hydroxy acid analysis in acidic hydrolysates of cereulide (**1**) and isocereulides H–N (2–8), after chiral derivatization

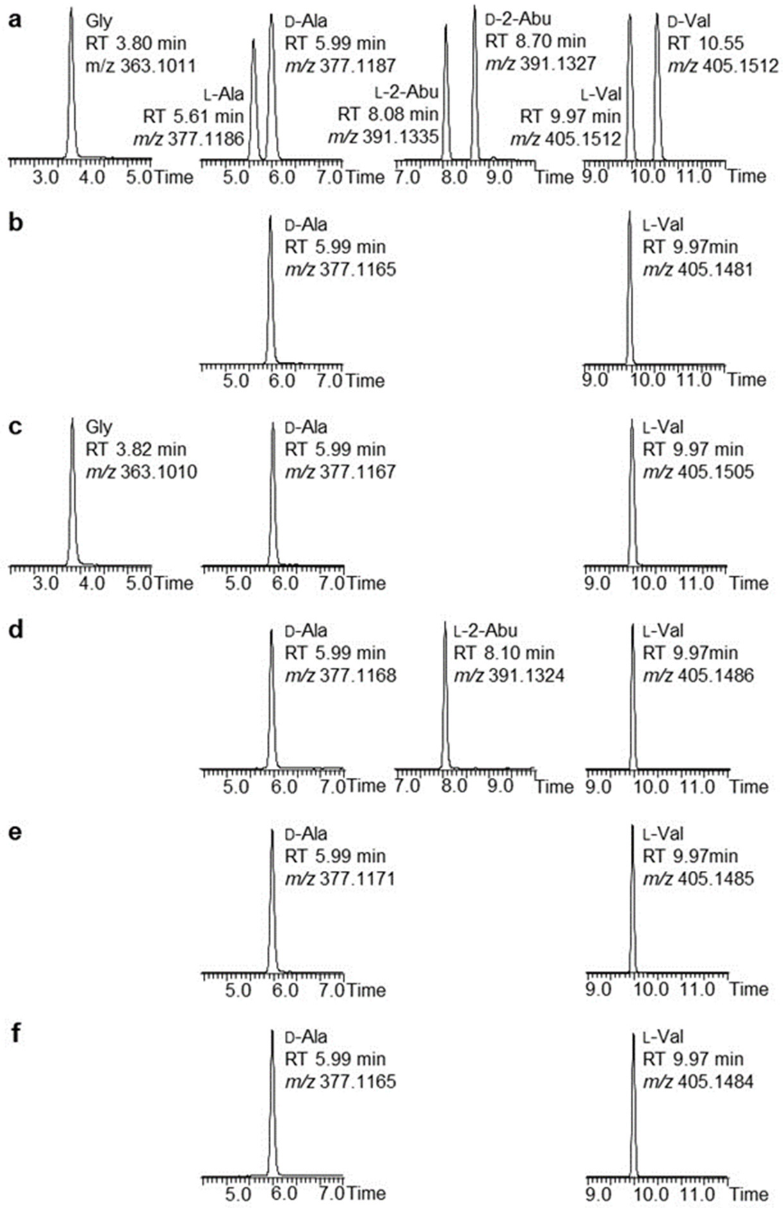


**Figure S11.** UPLC-ESI$^-$-TOF-MS-chromatograms of acidic hydrolysis after chiral amino acid derivatization of (**a**) amino acid references glycine, L- and D-alanine, L- and D-2-aminobutyric acid, and L- and D-valine, (**b**) cereulide (**1**), (**c**) isocereulide H (**2**), (**d**) isocereulide I (**3**), (**e**) isocereulide J (**4**), and (**f**) isocereulide K (**5**).

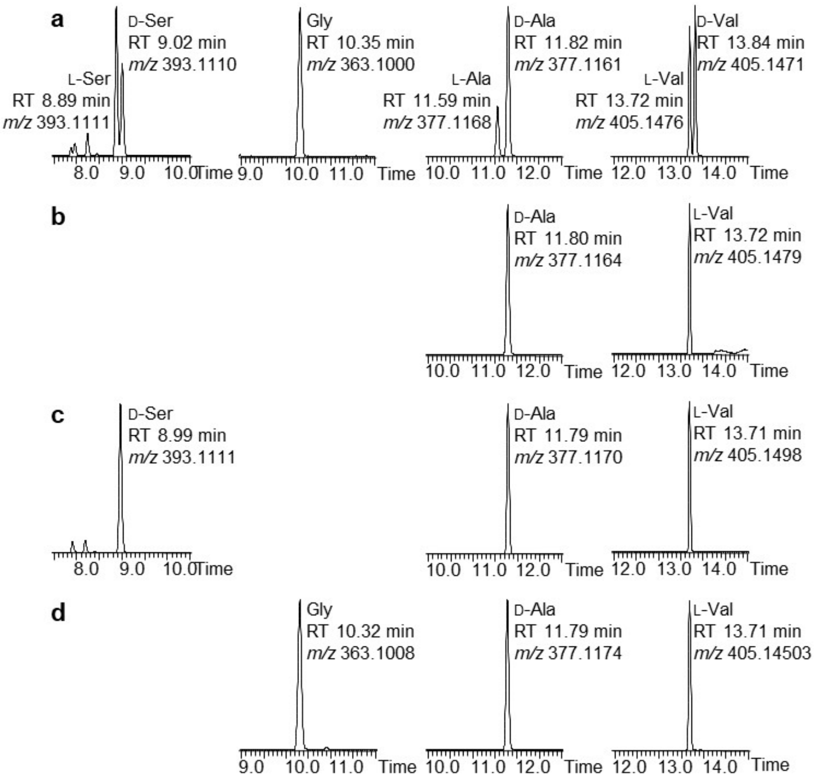


**Figure S12.** UPLC-ESI$^-$-TOF-MS-chromatograms of acidic hydrolysis after chiral amino acid derivatization of (**a**) amino acid references L- and D-serine, glycine, L- and D-alanine, and L- and D-valine, (**b**) cereulide (**1**), (**c**) isocereulide L and N (**6+8**), (**d**) isocereulide M (**7**).

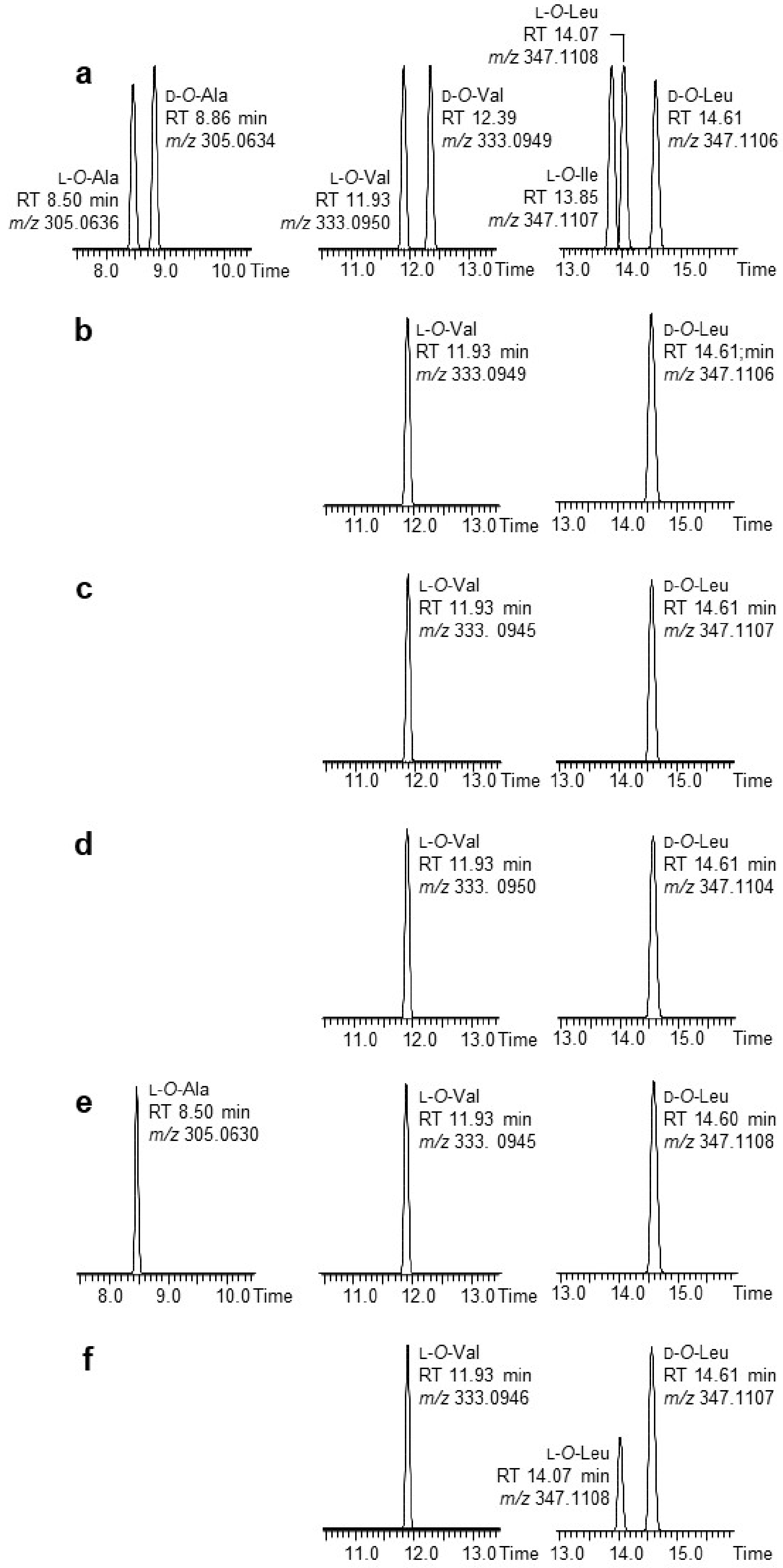


**Figure S13.** UPLC-ESI$^-$-TOF-MS-chromatograms of acidic hydrolysis after chiral $\alpha$-hydroxy acid derivatization of (**a**) $\alpha$-hydroxy acid references L- and D-*O*-alanine, L- and D-*O*-valine, and

L- and D-*O*-leucine with L-*O*-isoleucine, (**b**) cereulide (**1**), (**c**) isocereulide H (**2**), (**d**) isocereulide I (**3**), (**e**) isocereulide J (**4**), and (**f**) isocereulide K (**5**).

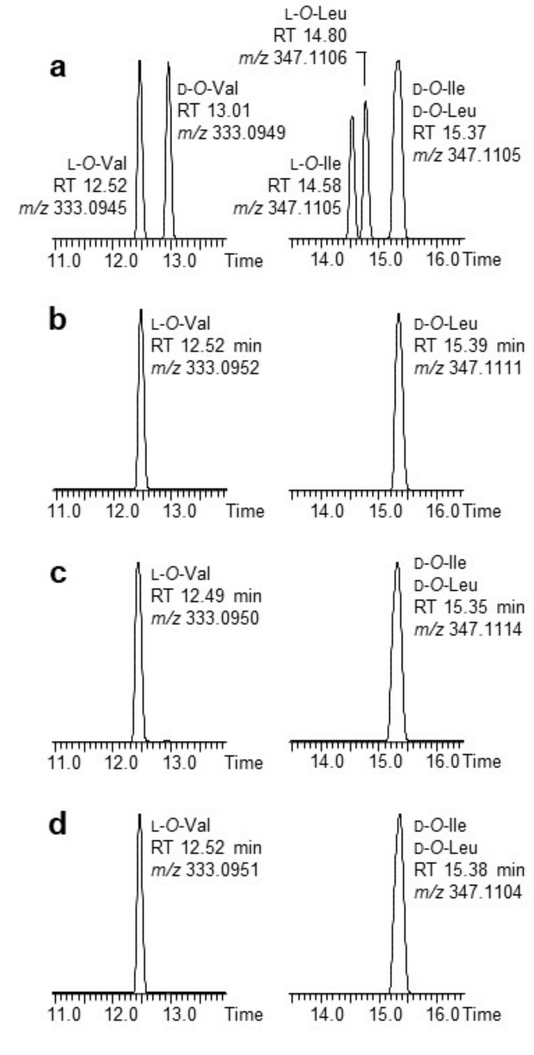


**Figure S14.** UPLC-ESI$^-$-TOF-MS-chromatograms of acidic hydrolysis after chiral $\alpha$-hydroxy acid derivatization of (**a**) $\alpha$-hydroxy acid references L- and D-*O*-valine, and L- and D-*O*-leucine with L- and D-*O*-isoleucine, (**b**) cereulide (**1**), (**c**) isocereulide L and N (**6+8**), (**d**) isocereulide M (**7**).

E. 1D- and 2D-NMR spectra of isocereulides H (**2**), K (**5**), L and N (**6+8**), and M (**7**)

NMR data and 1D- and 2D-NMR spectra of cereulide (**1**) were recently published.[2]

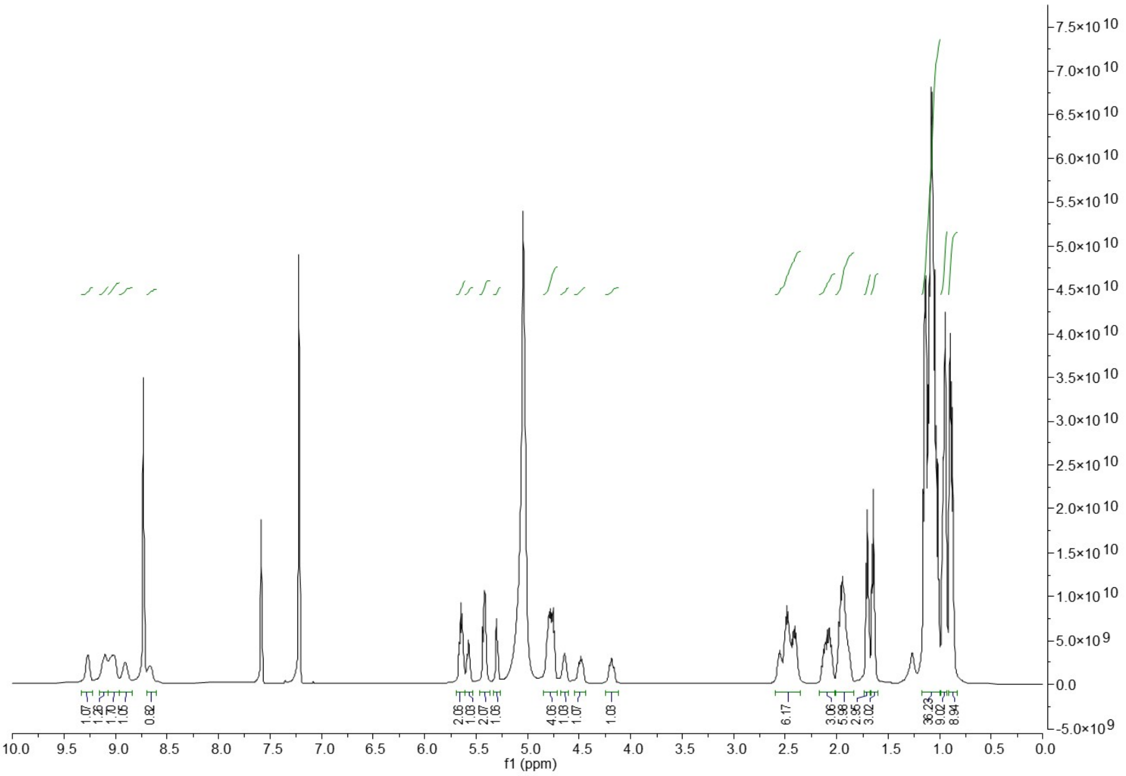

**Figure S15.** $^{1}$H-NMR spectrum of **2** (600 MHz, 298 K, pyridine-$d_5$).

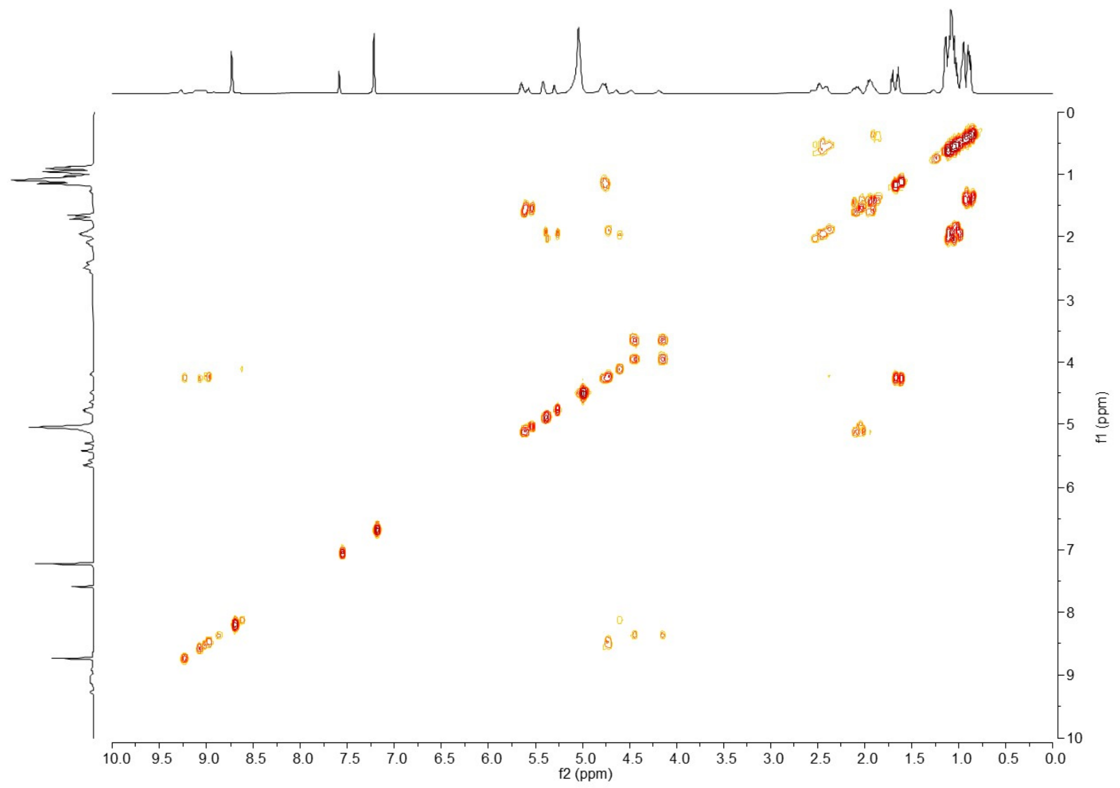

**Figure S16.** $^{1}$H,$^{1}$H-COSY-NMR spectrum of **2** (600 MHz, 298 K, pyridine-$d_5$).

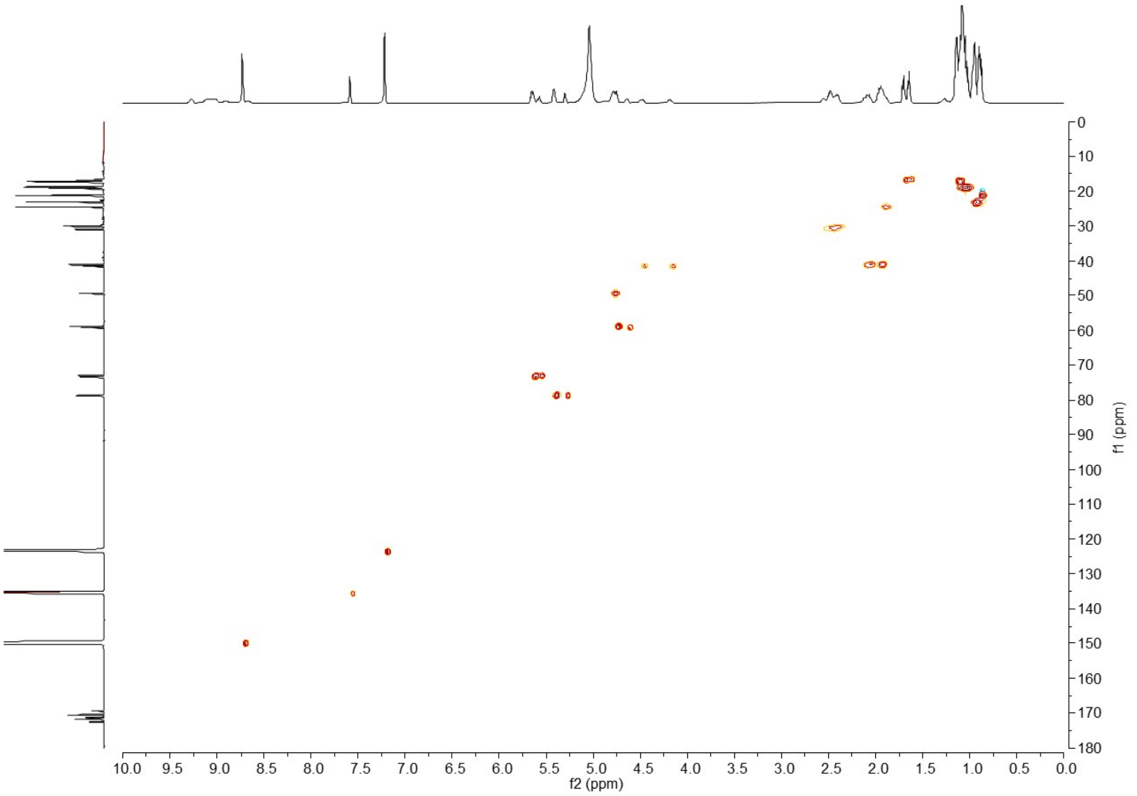


**Figure S17.** $^{1}H,^{13}C$-HSQC-NMR spectrum of **2** (600 MHz, 150 MHz, 298 K, pyridine-*d*5).

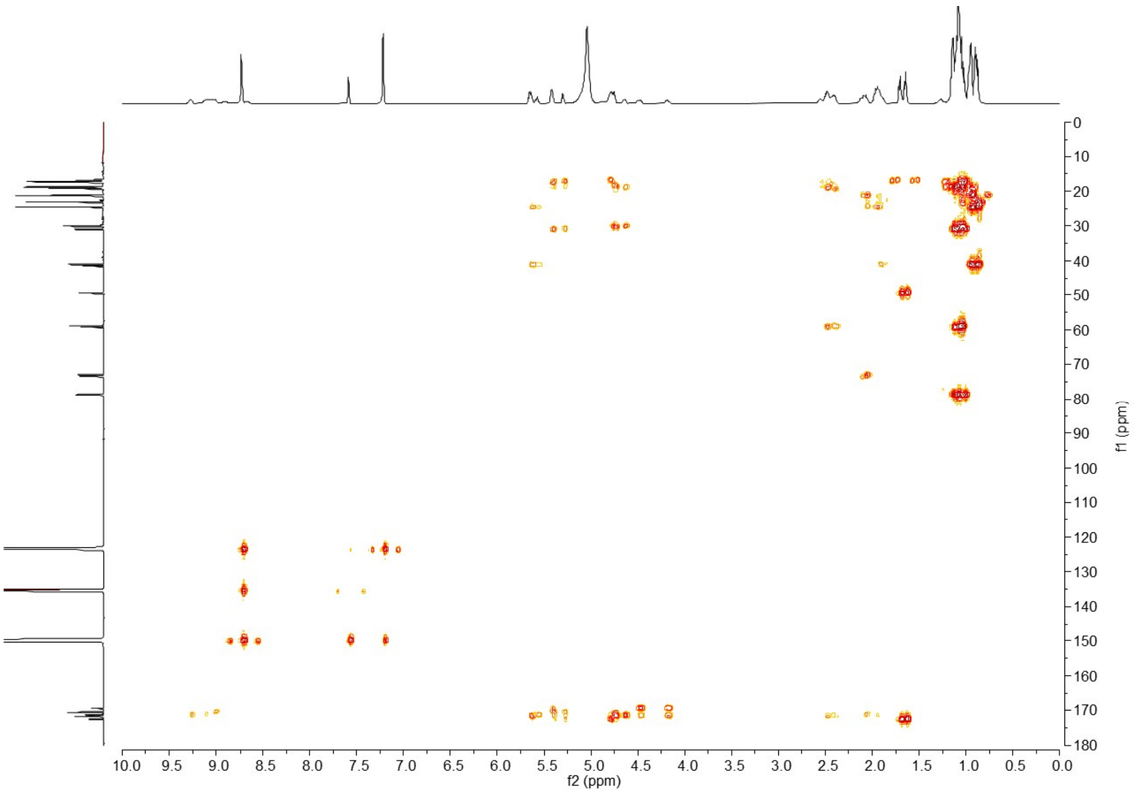


**Figure S18.** $^{1}H,^{13}C$-HMBC-NMR spectrum of **2** (600 MHz, 150 MHz, 298 K, pyridine-*d*5).

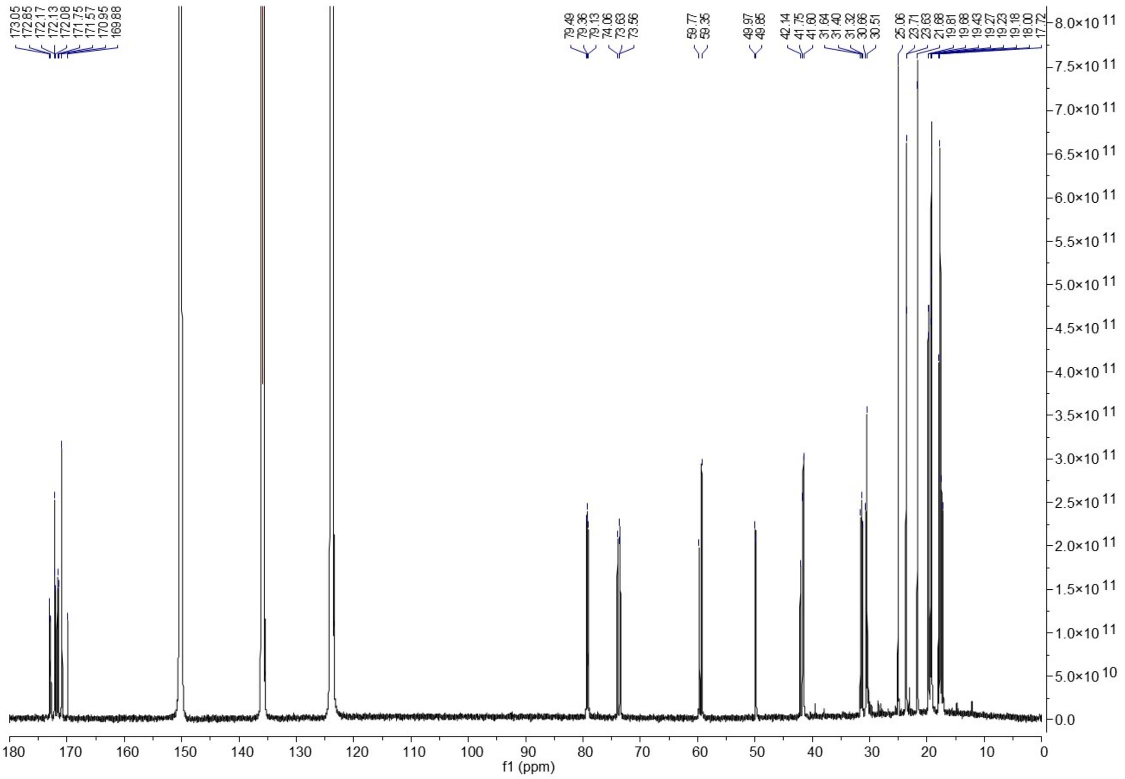

**Figure S19.** $^{13}$C-NMR spectrum of **2** (150 MHz, 298 K, pyridine-$d_5$).

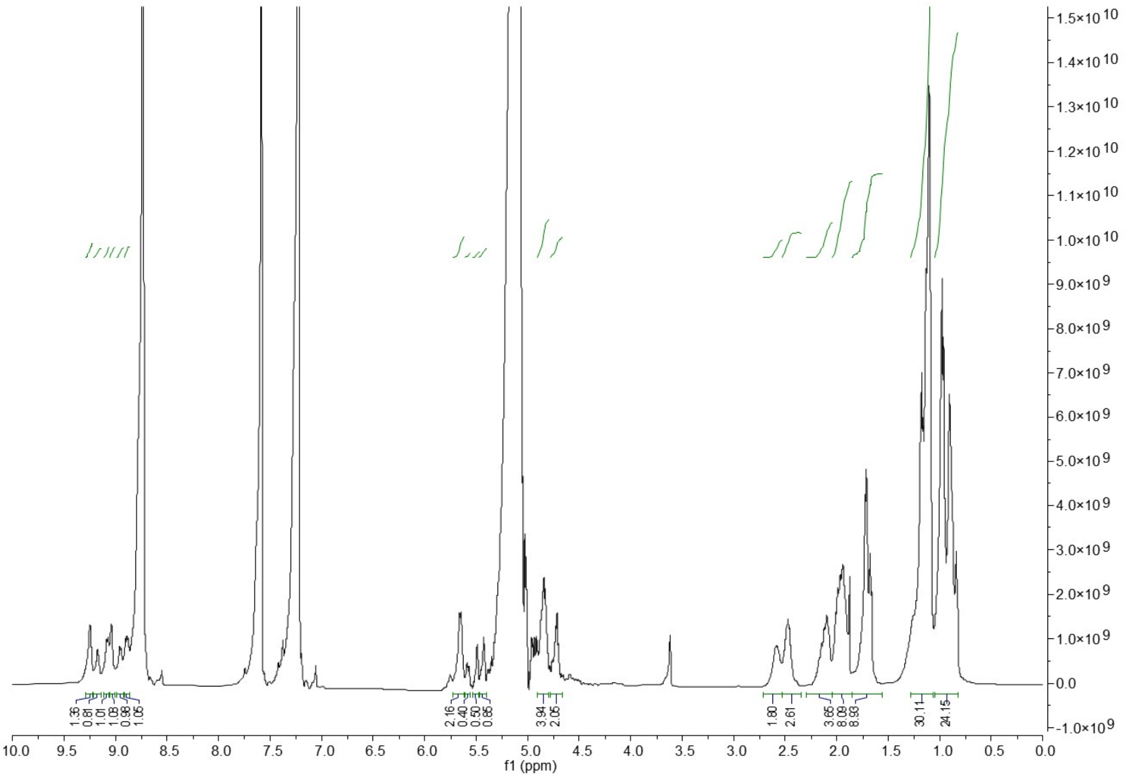

**Figure S20.** $^{1}$H-NMR spectrum of **5** (500 MHz, 298 K, pyridine-$d_5$).

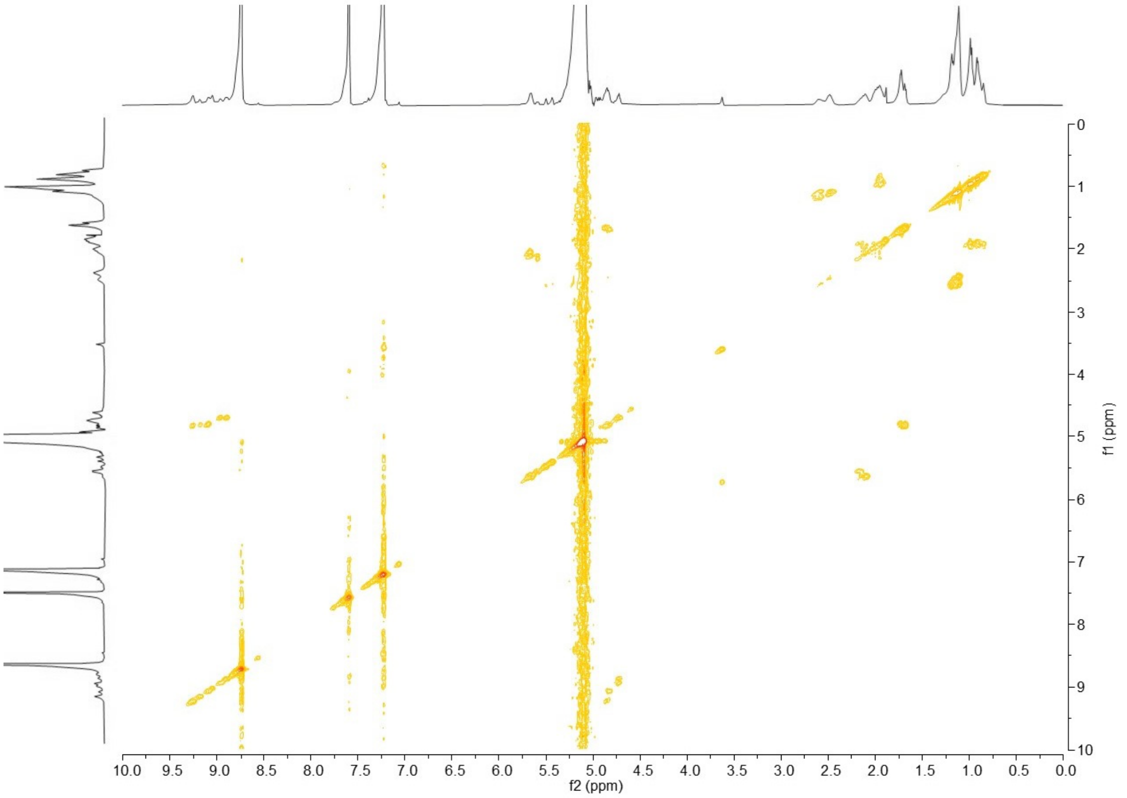


**Figure S21.** $^1H$, $^1H$-COSY-NMR spectrum of **5** (500 MHz, 298 K, pyridine-$d_5$).

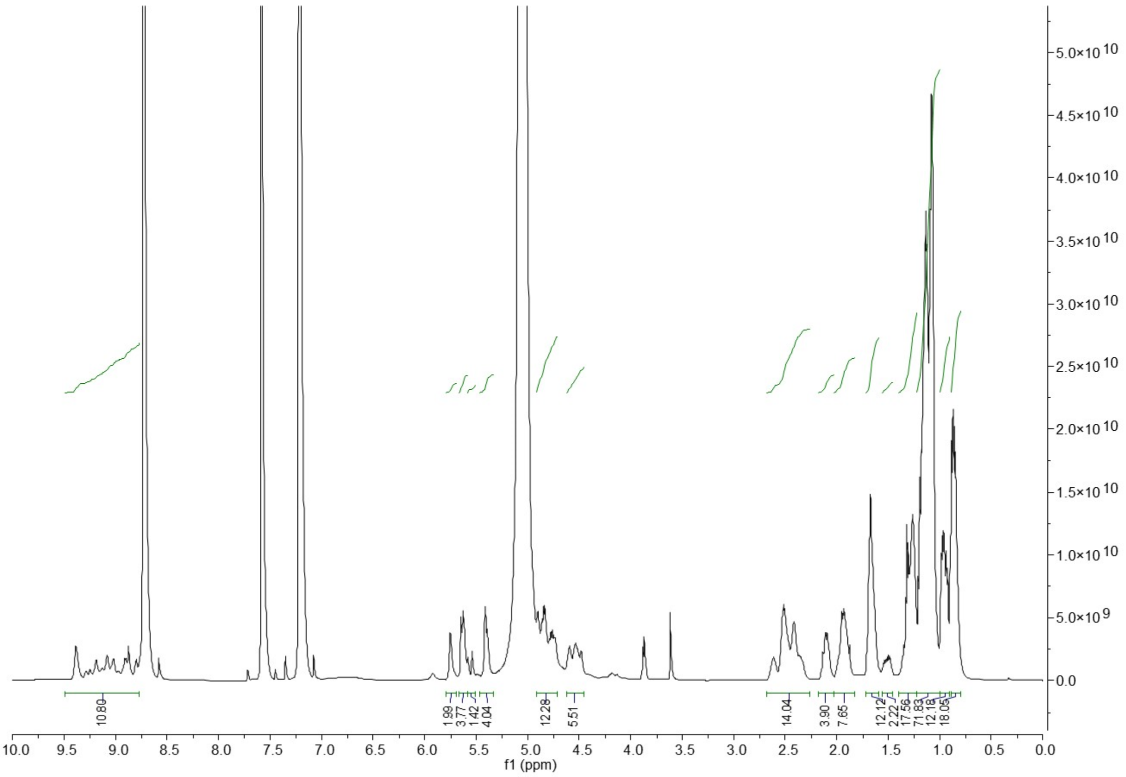


**Figure S22.** $^1H$-NMR spectrum of a mixture of **6** and **8** (600 MHz, 298 K, pyridine-$d_5$).

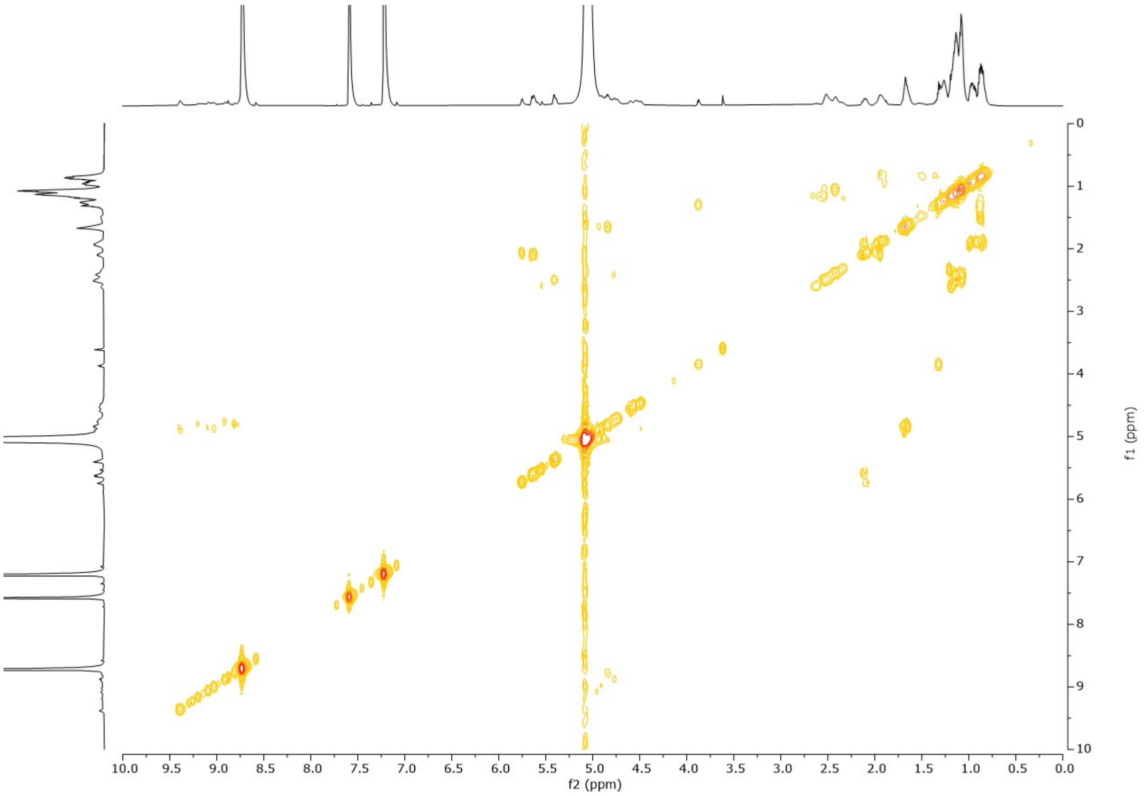


**Figure S23.** $^1$H,$^1$H-COSY-NMR spectrum of a mixture of **6** and **8** (600 MHz, 298 K, pyridine-*d*$_5$).

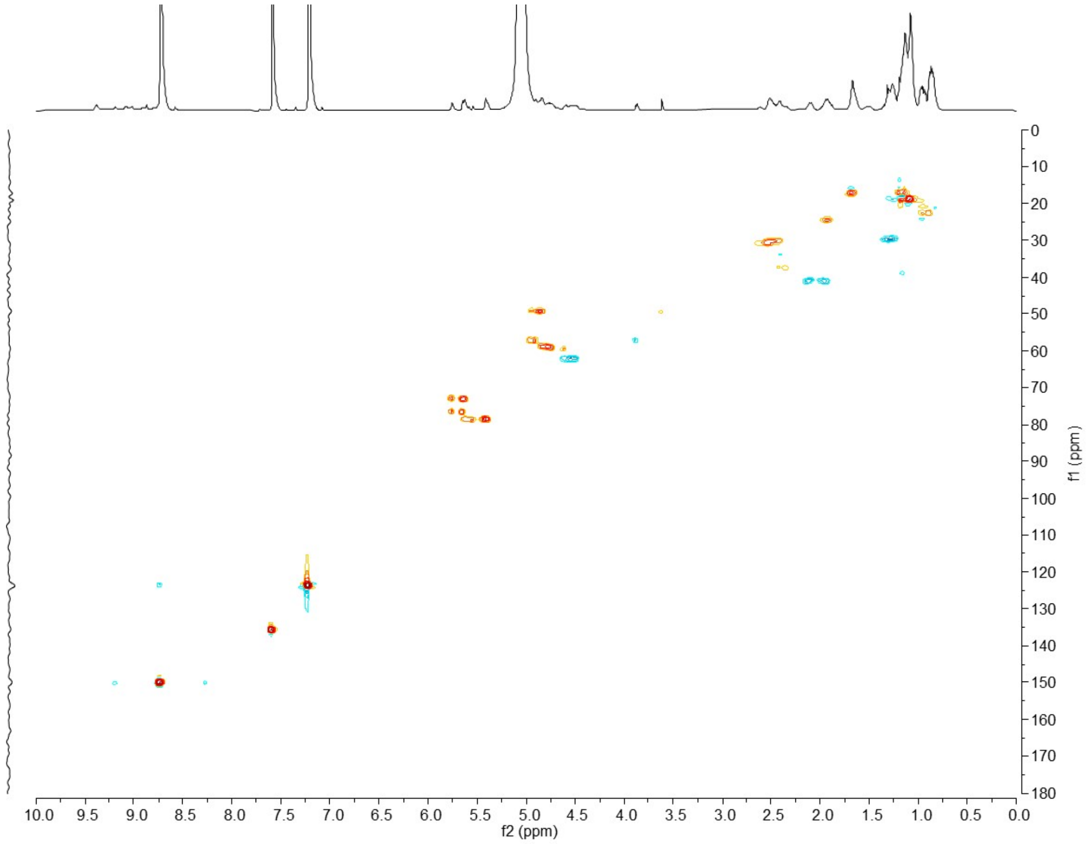


**Figure S24.** $^1$H,$^{13}$C-HSQC-NMR spectrum of a mixture of **6** and **8** (600 MHz, 150 MHz, 298 K, pyridine-*d*$_5$).

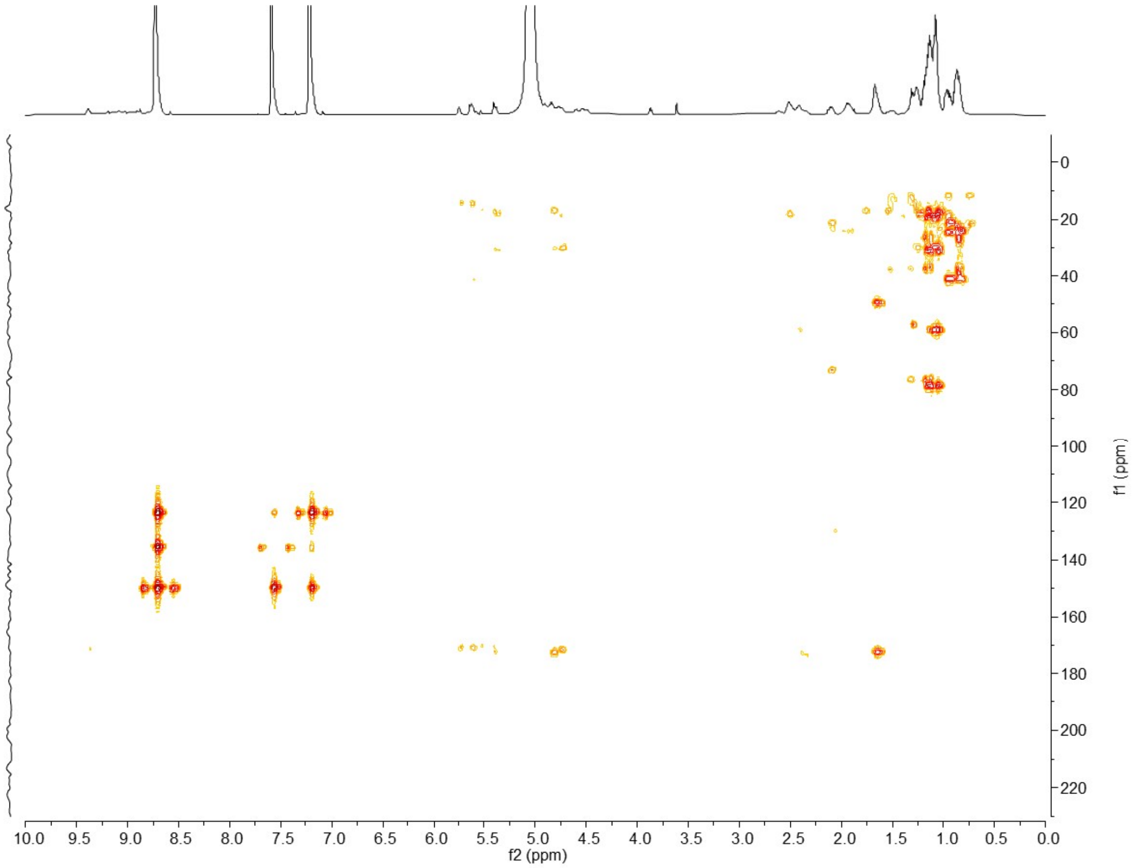


**Figure S25.** $^{1}$H,$^{13}$C-HMBC-NMR spectrum of a mixture of **6** and **8** (600 MHz, 150 MHz, 298 K, pyridine-$d_5$).

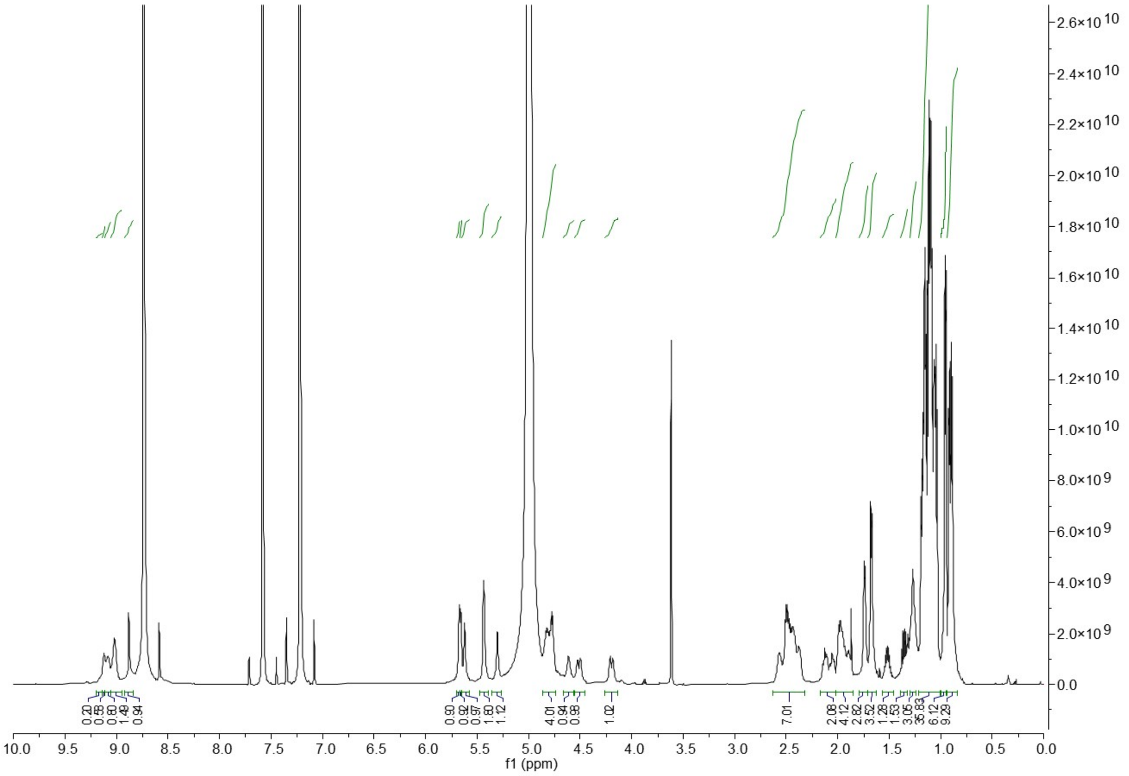


**Figure S26.** $^{1}$H-NMR spectrum of **7** (600 MHz, 298 K, pyridine-$d_5$).

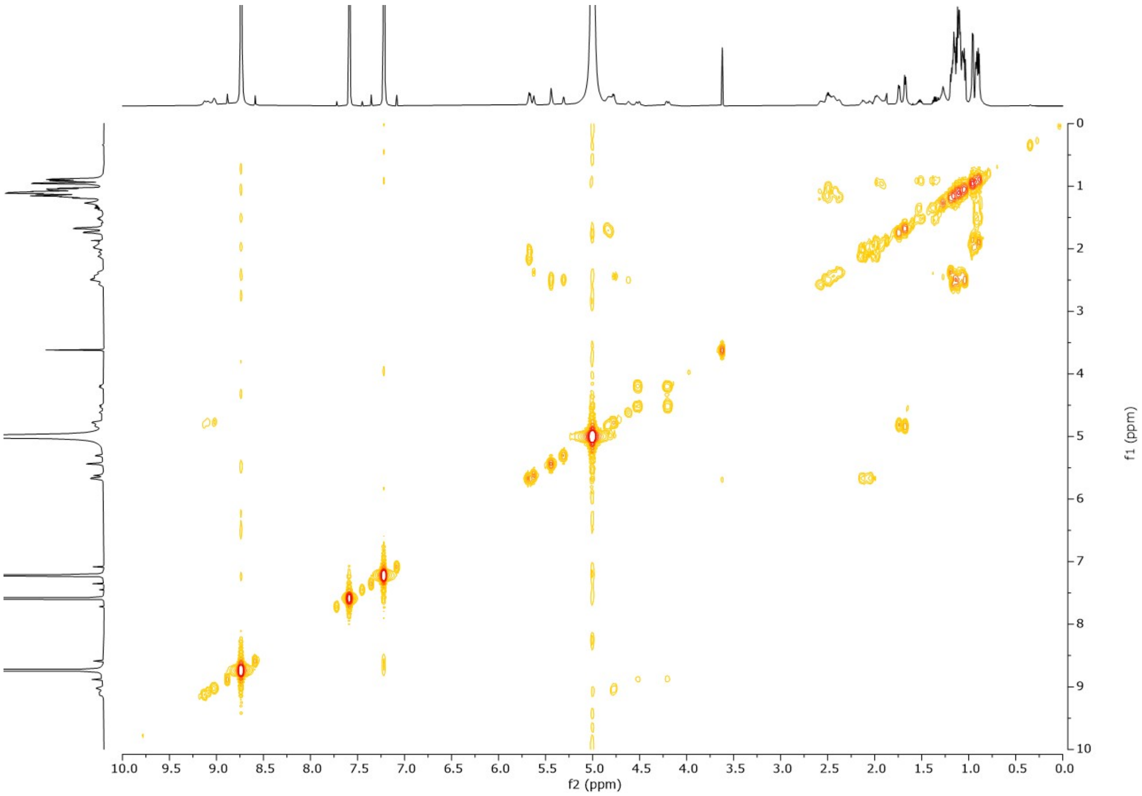

**Figure S27.** $^{1}H$, $^{1}H$-COSY-NMR spectrum of **7** (600 MHz, 298 K, pyridine-*d*5).

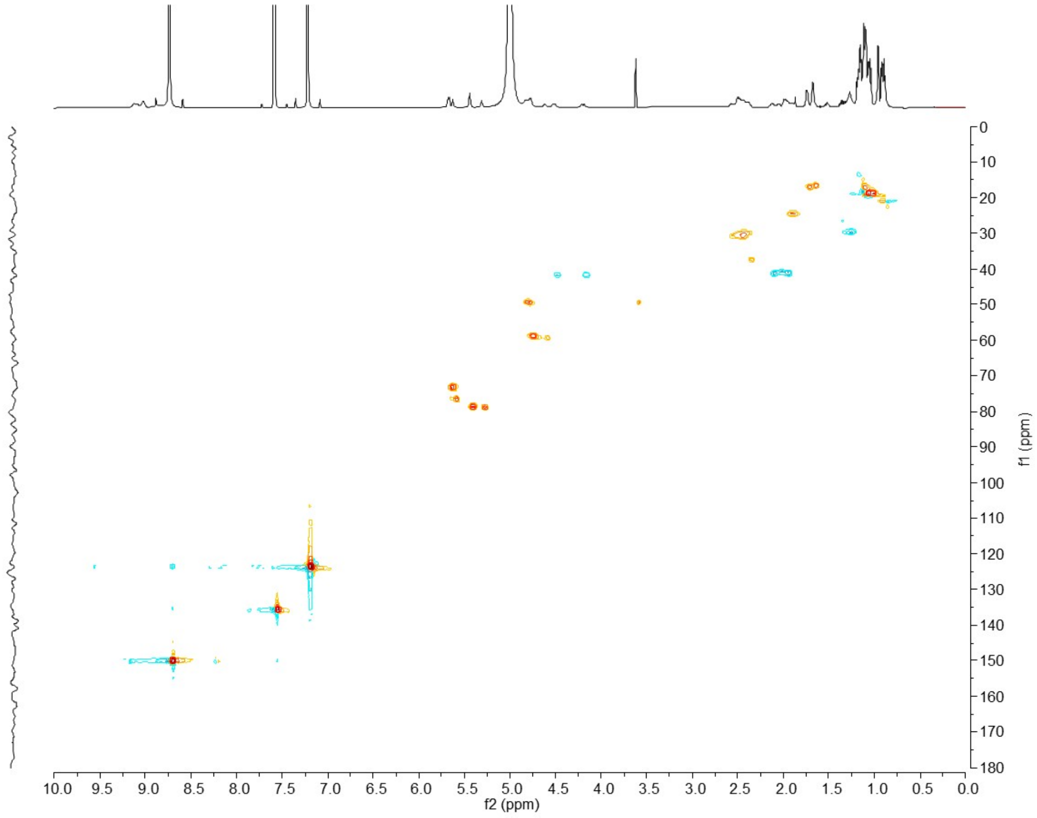

**Figure S28.** $^{1}H$,$^{13}C$-HSQC-NMR spectrum of **7** (600 MHz, 150 MHz, 298 K, pyridine-*d*5).

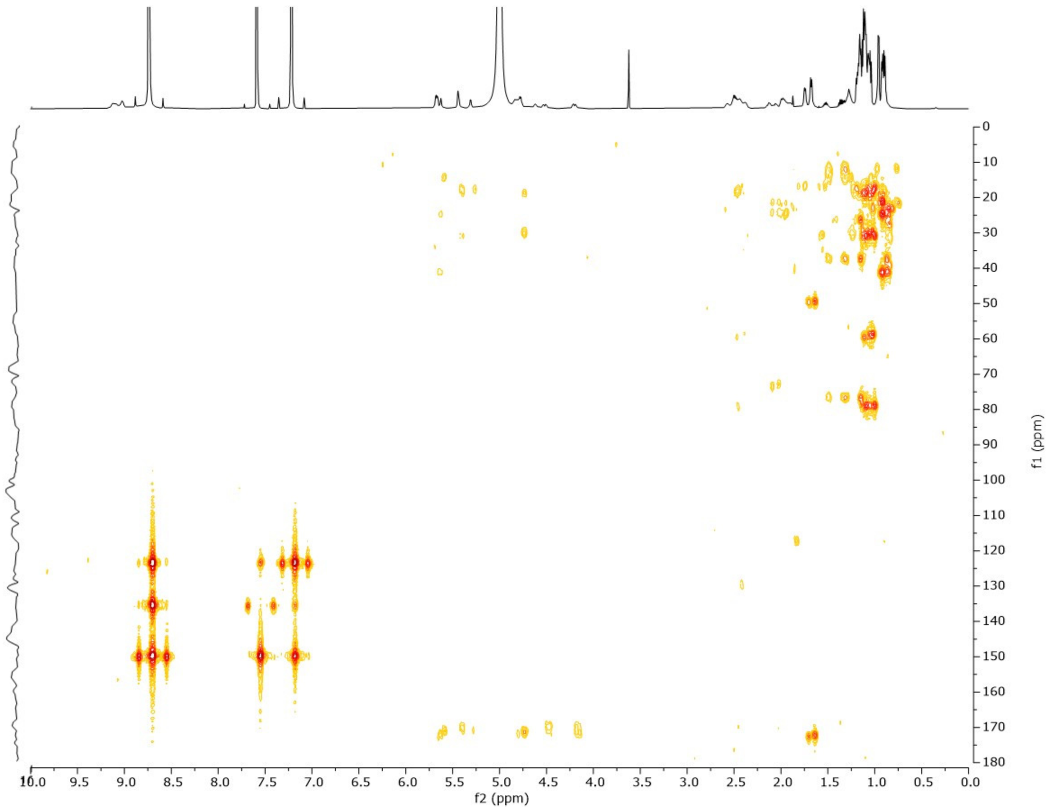


**Figure S29.** $^{1}H,^{13}C$-HMBC-NMR spectrum of **7** (600 MHz, 150 MHz, 298 K, pyridine-$d_5$).

F. $MS^n$-Data of cereulide (**1**) and isocereulides H–N (**2–8**)

**Cereulide (1):**

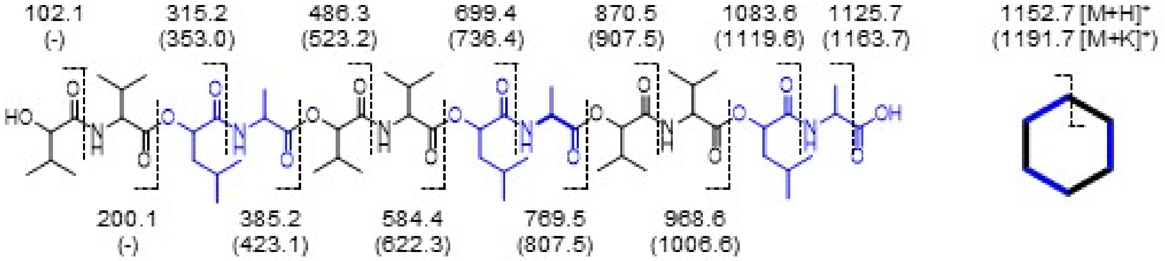


MS

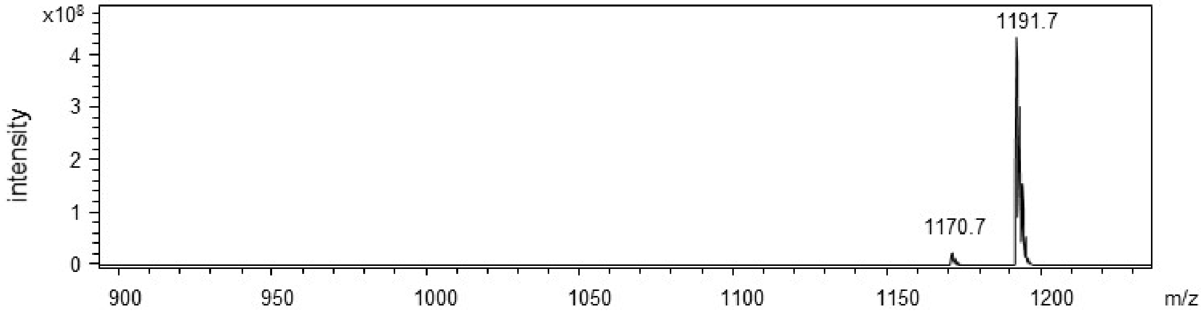


$MS^2$ (1191.7 → x)

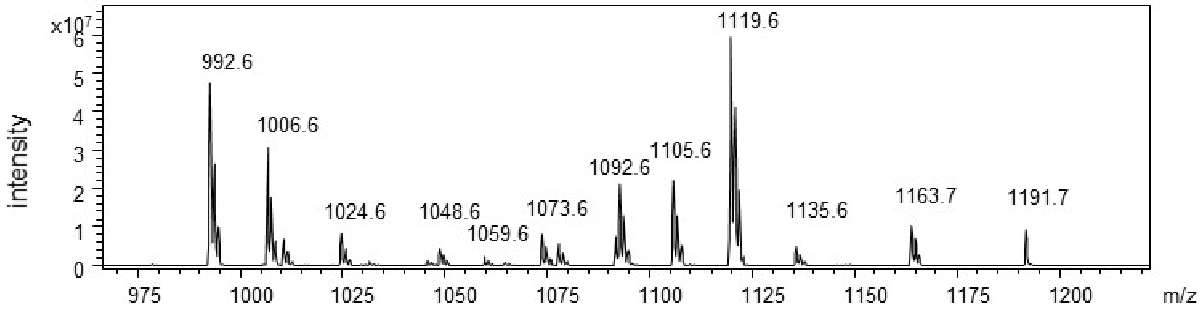


$MS^3$ (1006.5 → x)

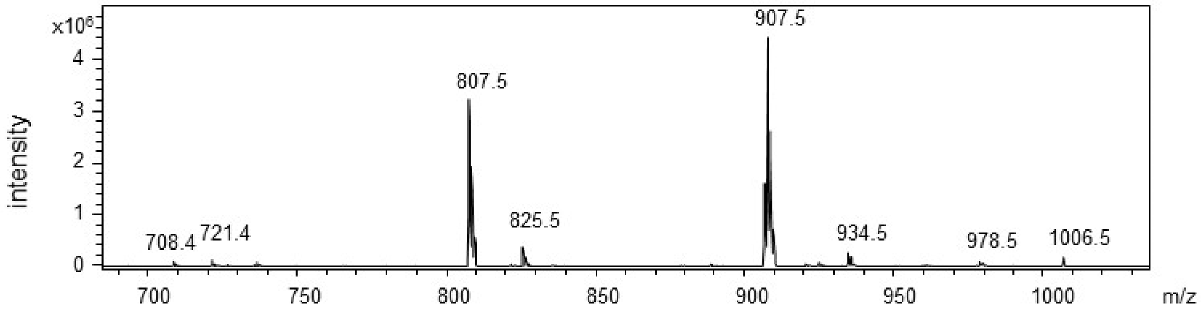

MS$^4$ (807.4 → x)

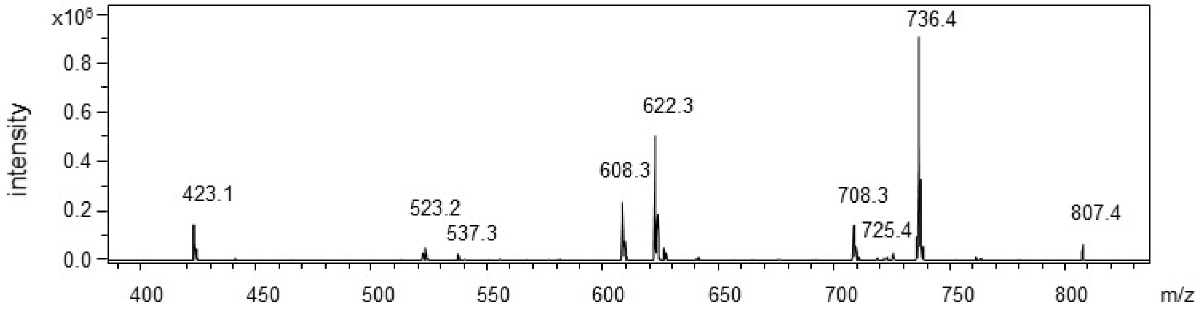


MS$^5$ (622.3 → x)

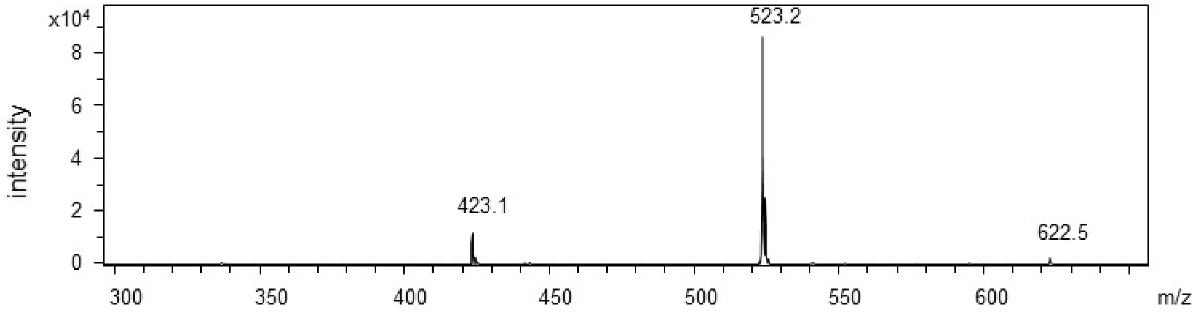


MS$^6$ (423.1 → x)

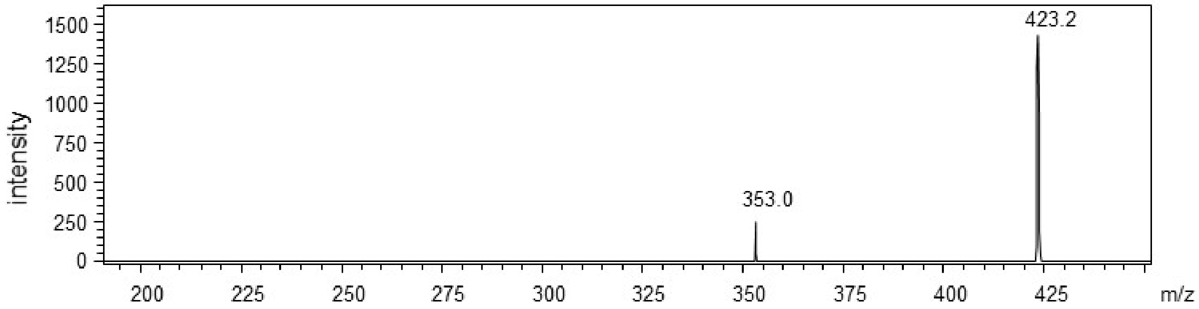


**Isocereulide H (2):**

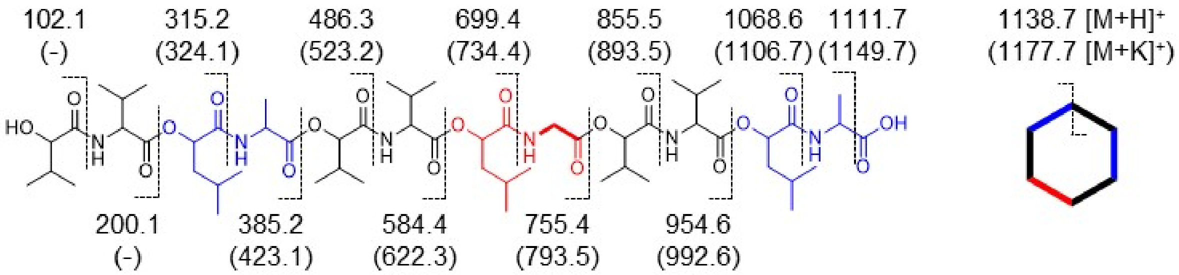

MS

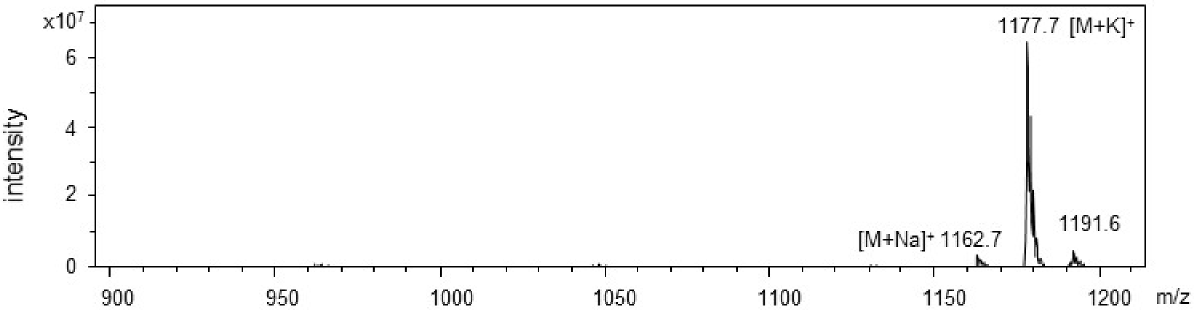


MS$^2$ (1177.7 → x)

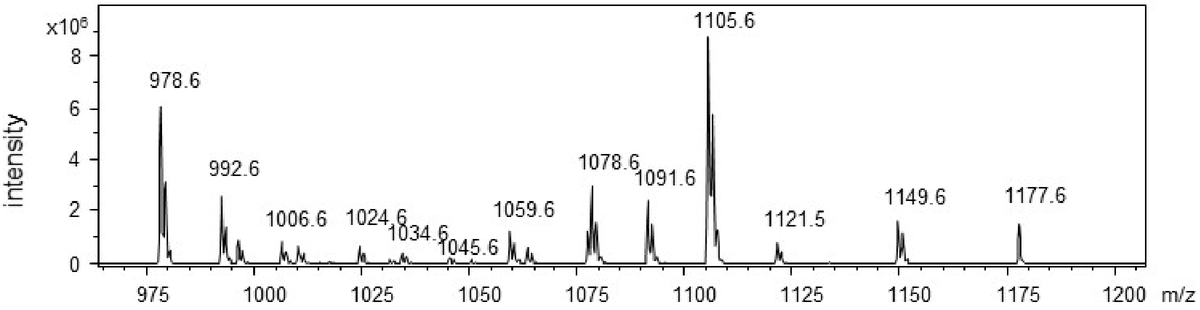


MS$^3$ (992.5 → x)

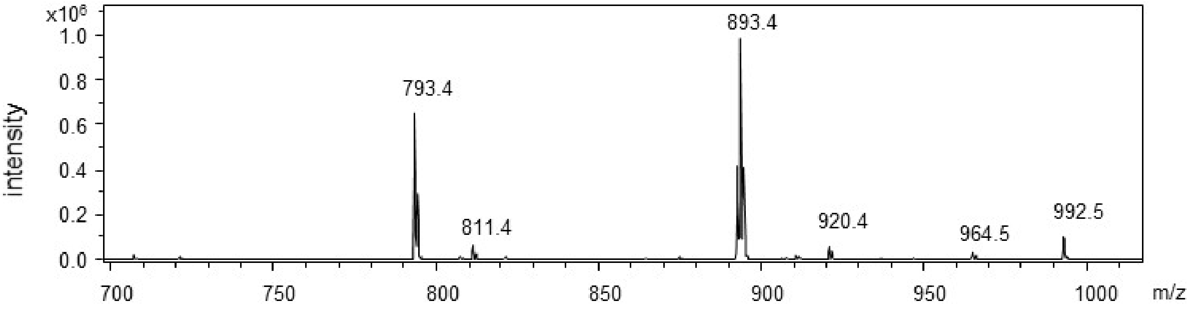


MS$^4$ (793.4 → x)

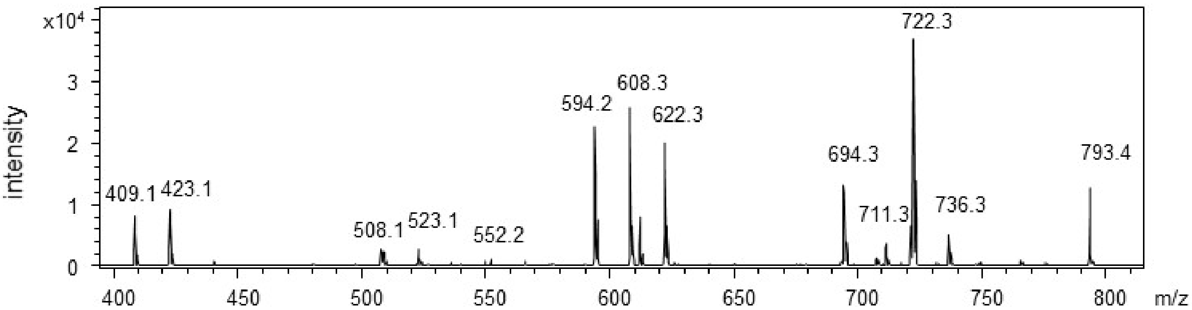

MS$^5$ (622.3 → x)

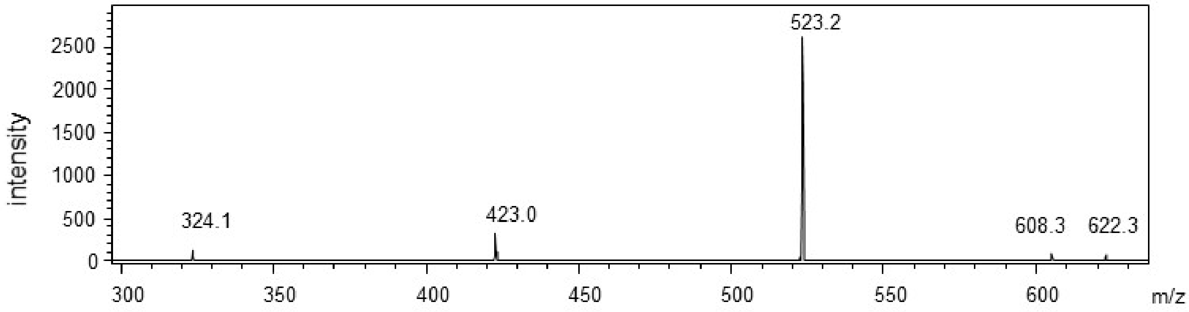


**Isocereulide I (3):**

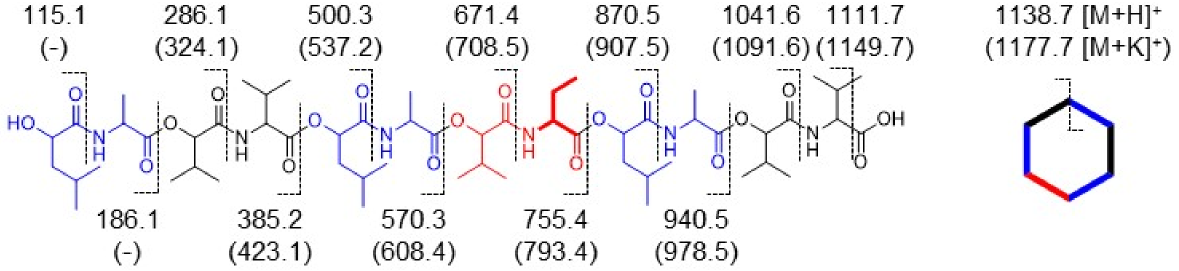


MS

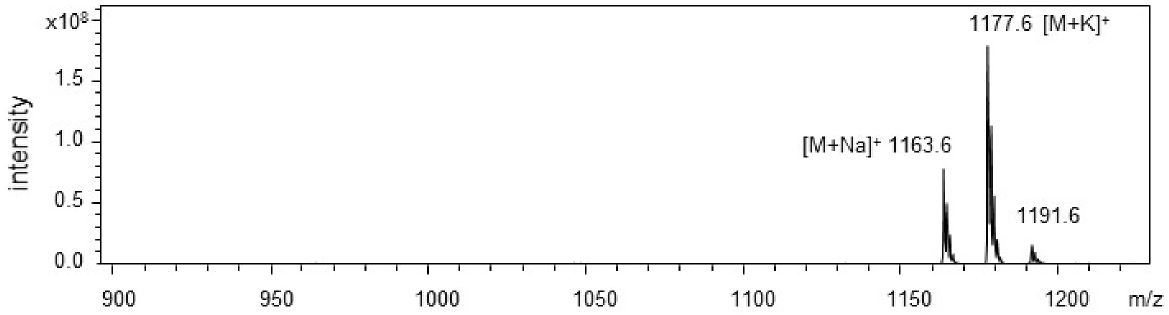


MS$^2$ (1177.7 → x)

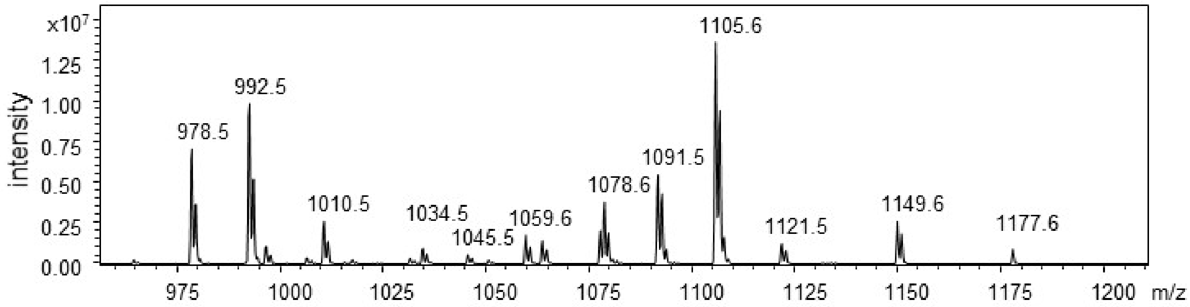

MS$^3$ (978.5 → x)

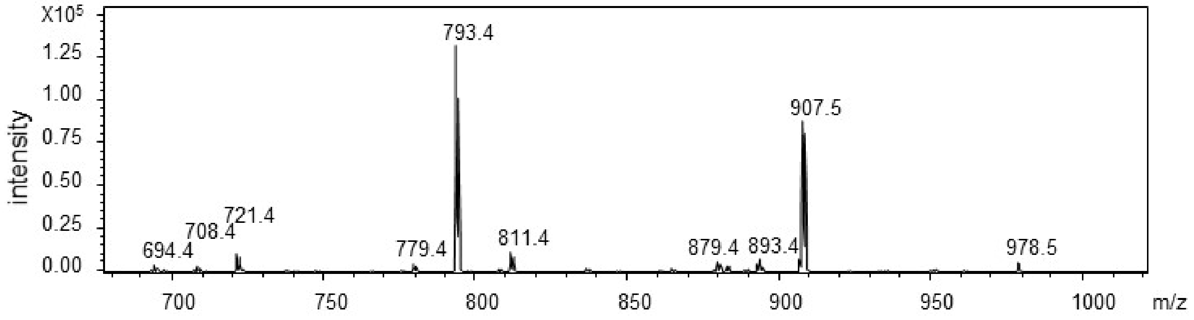


MS$^4$ (793.4 → x)

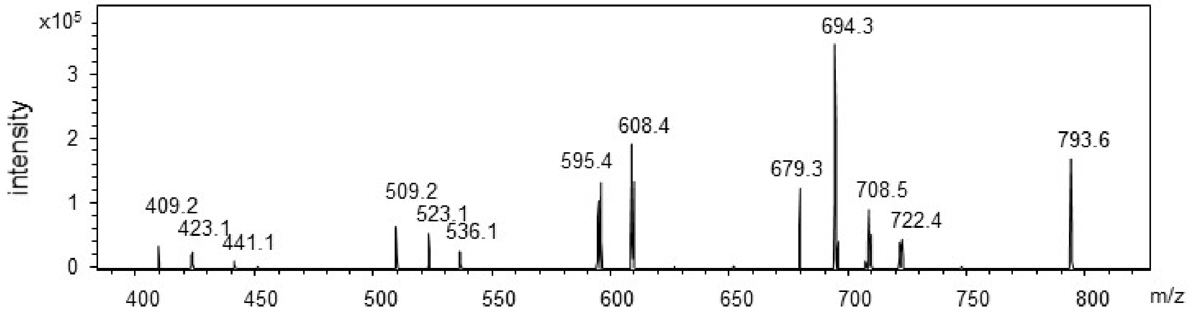


MS$^5$ (608.4 → x)

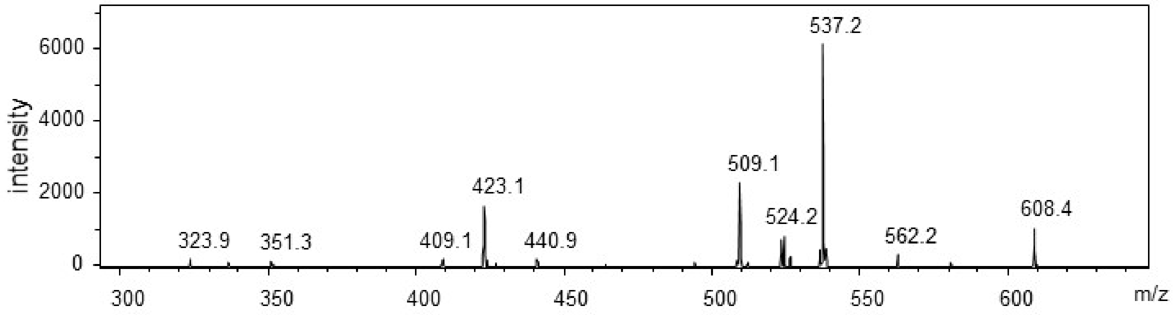


MS$^6$ (423.1 → x)

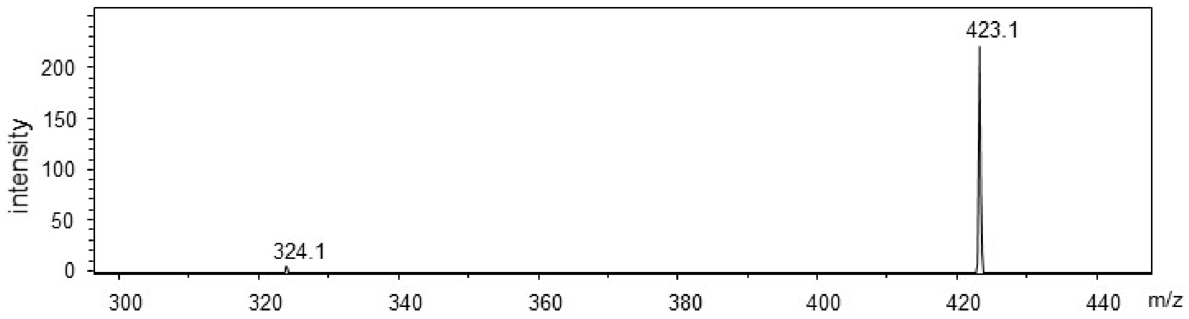

**Isocereulide J (4):**

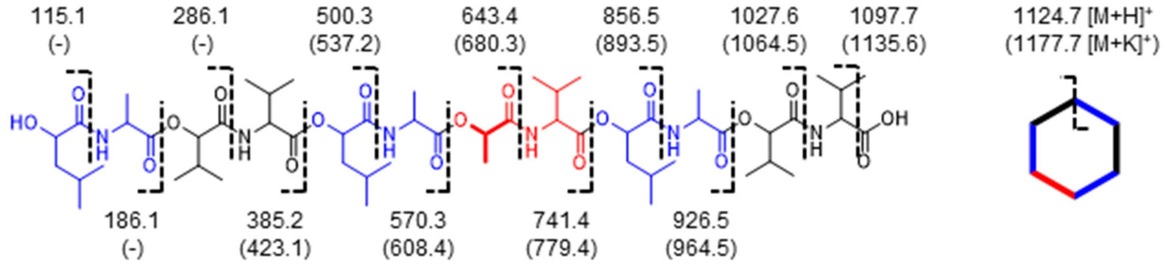


MS

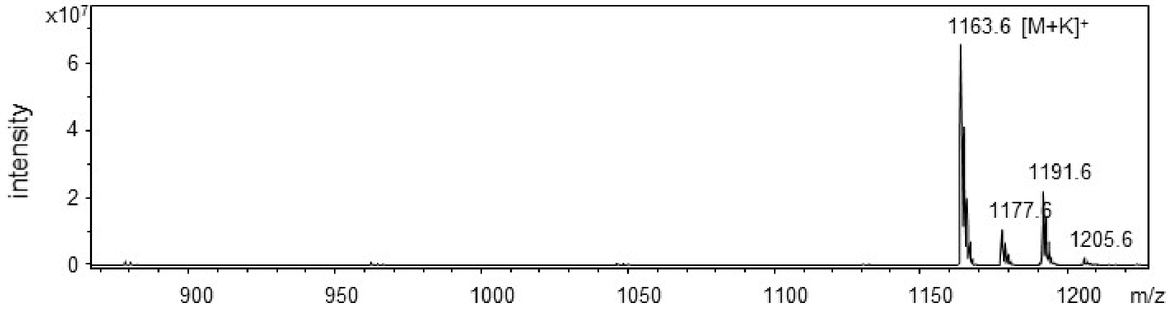


MS$^2$ (1163.7 → x)

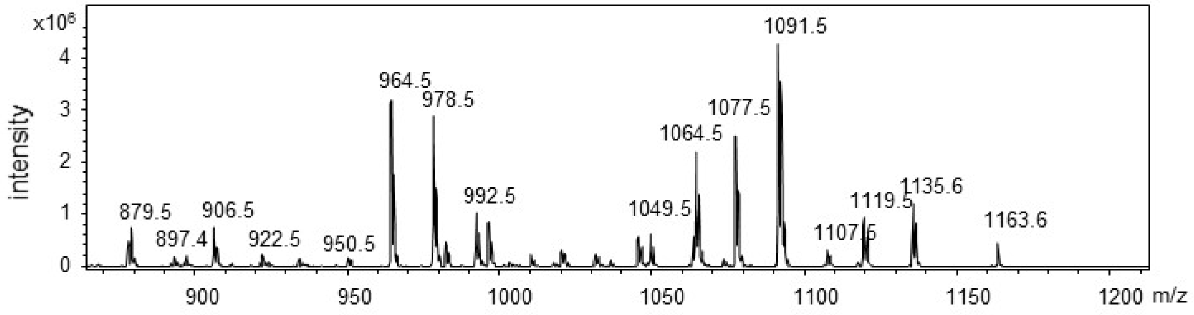


MS$^3$ (964.5 → x)

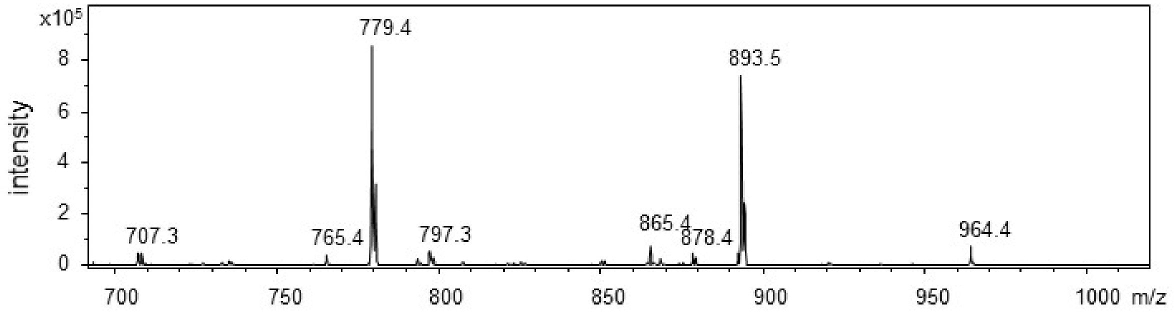

MS$^4$ (779.3 → x)

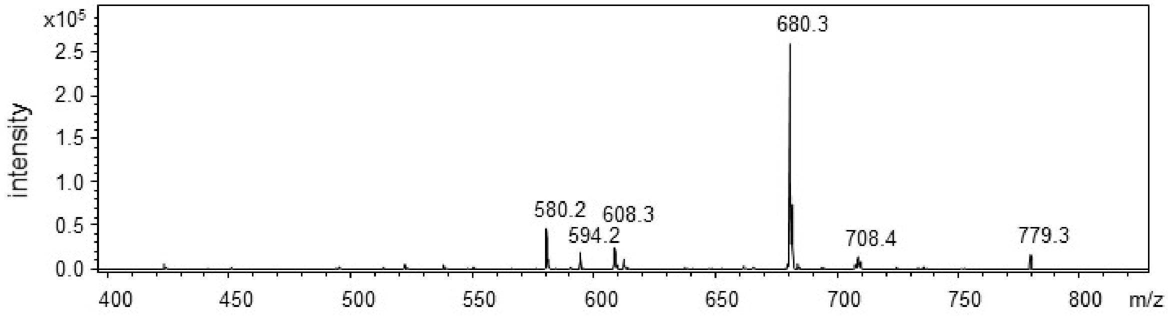


MS$^5$ (608.3 → x)

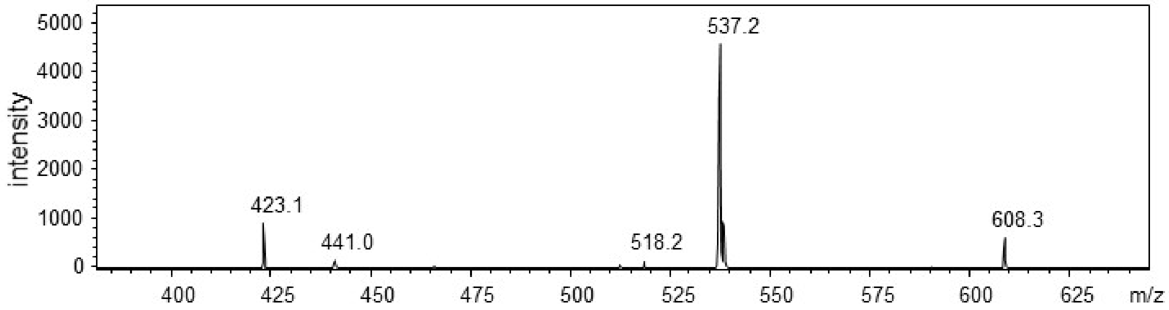


MS$^6$ (537.3 → x)

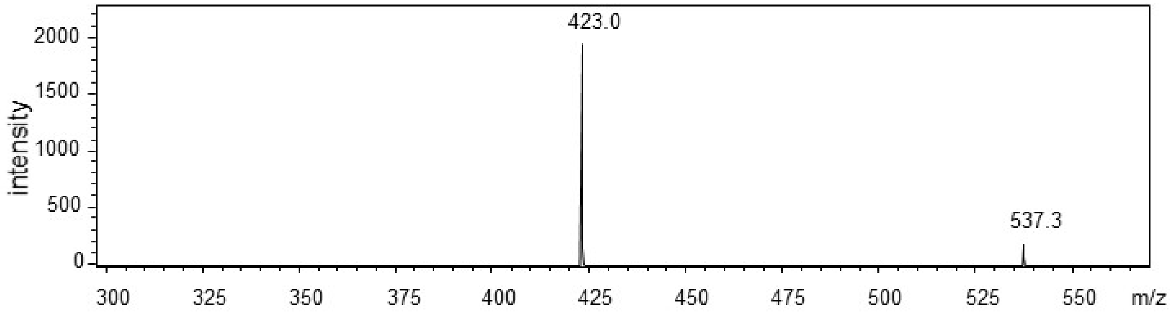


**Isocereulide K (5):**

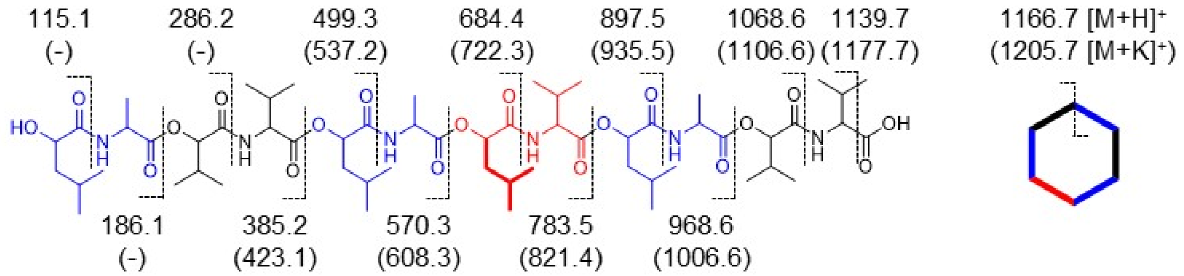

MS

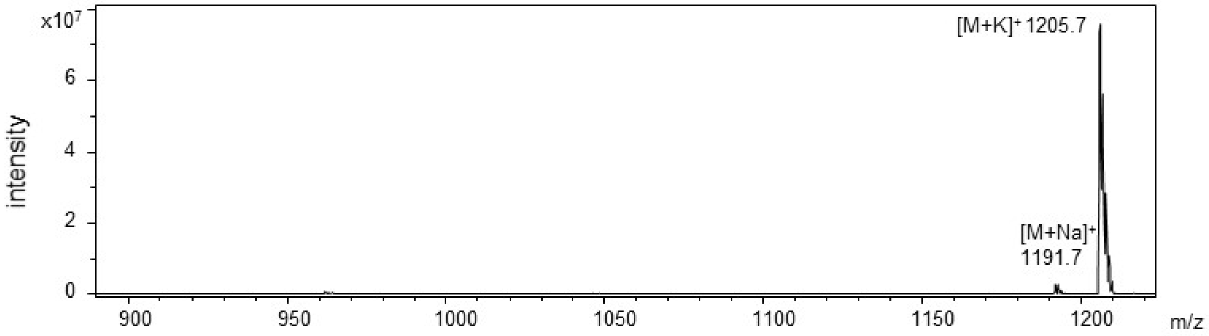


MS$^{2}$ (1205.7 → x)

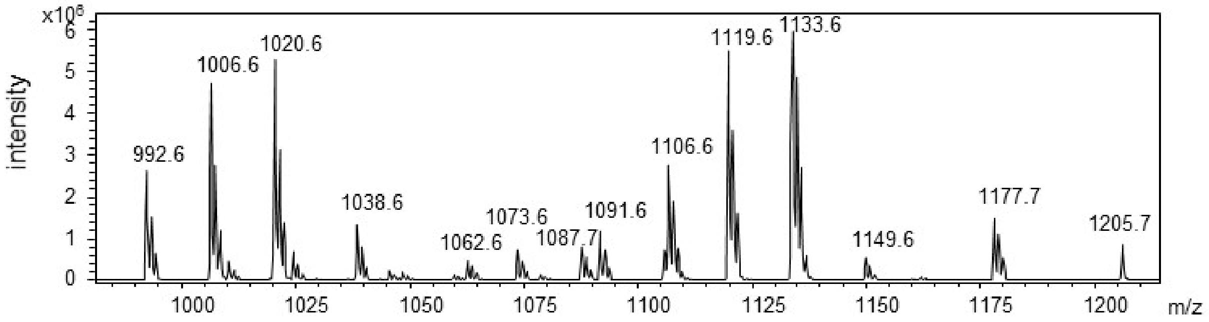


MS$^{3}$ (1006.5 → x)

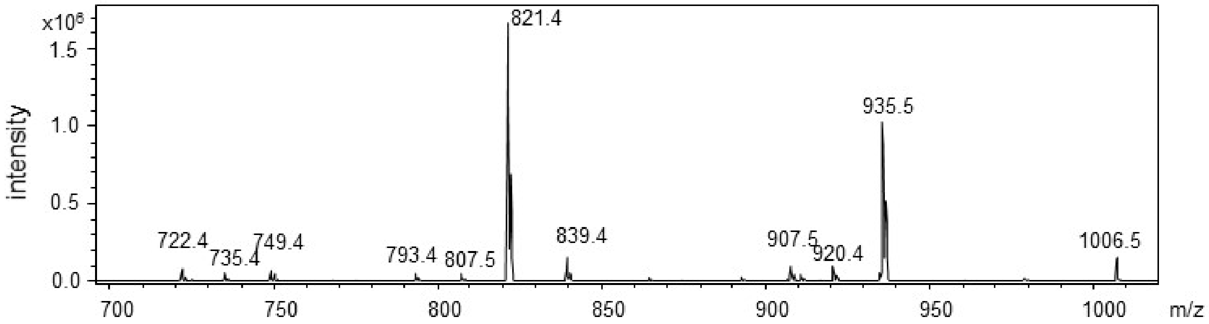


MS$^{4}$ (821.4 → x)

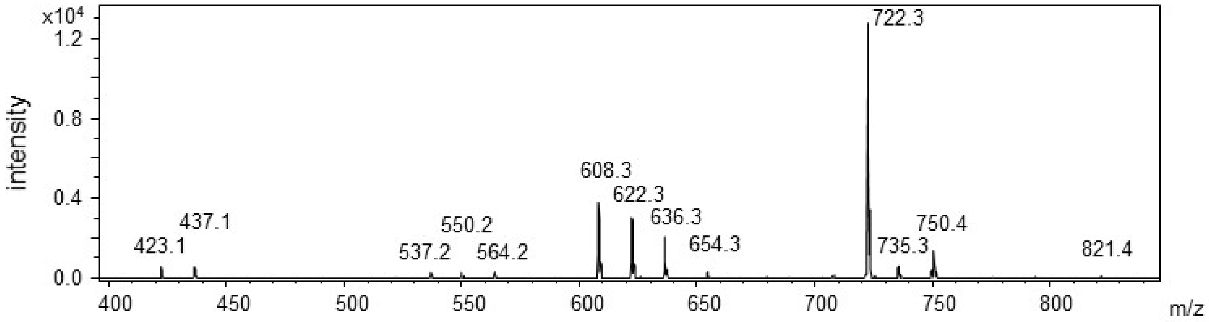

MS$^5$ (608.3 → x)

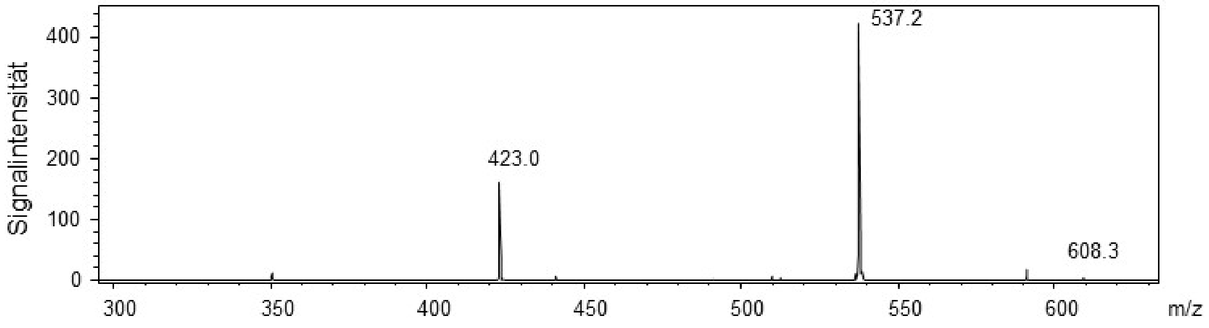


**Isocereulide L (6, upper structure) and N (8, lower structure):**

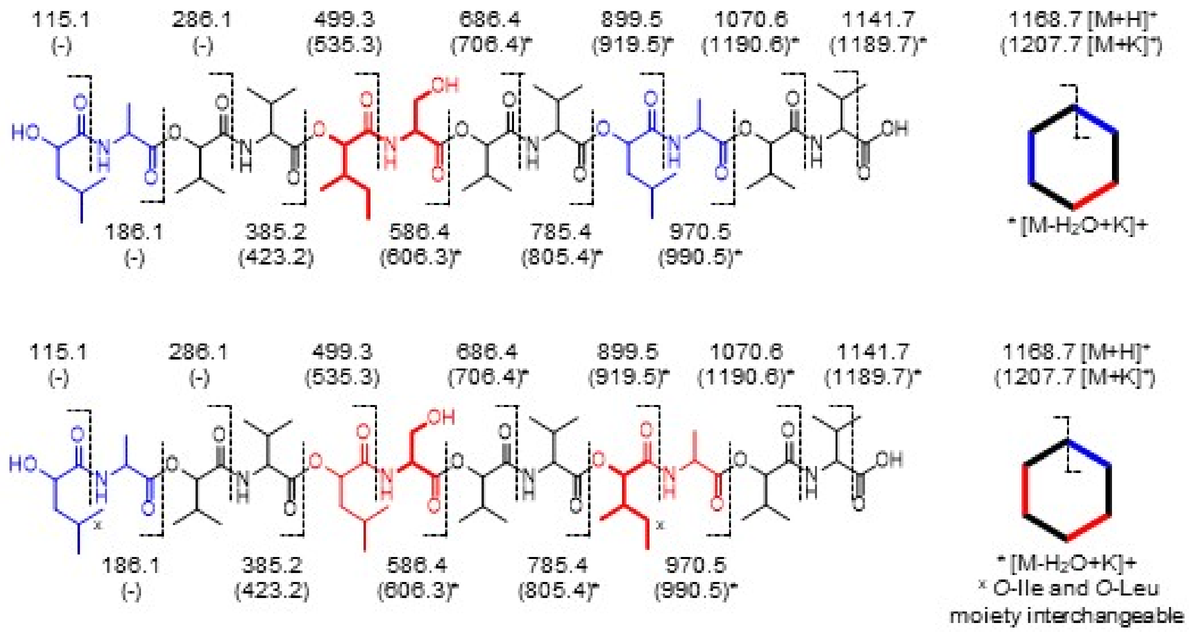


MS

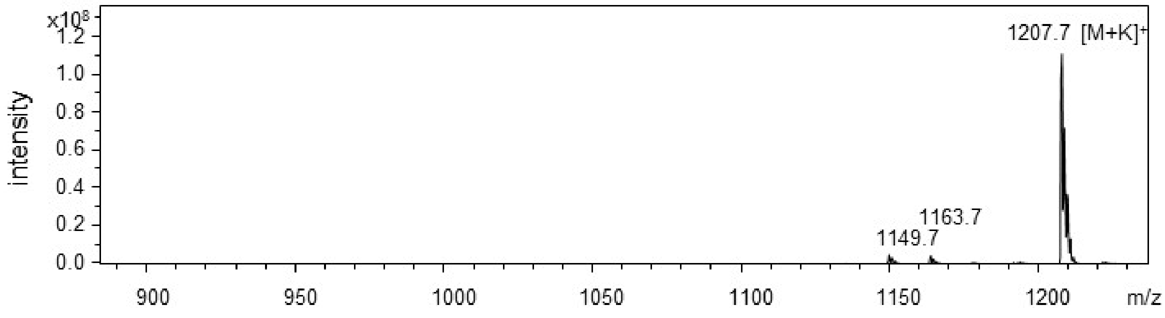

MS$^2$ (1207.7 → x)

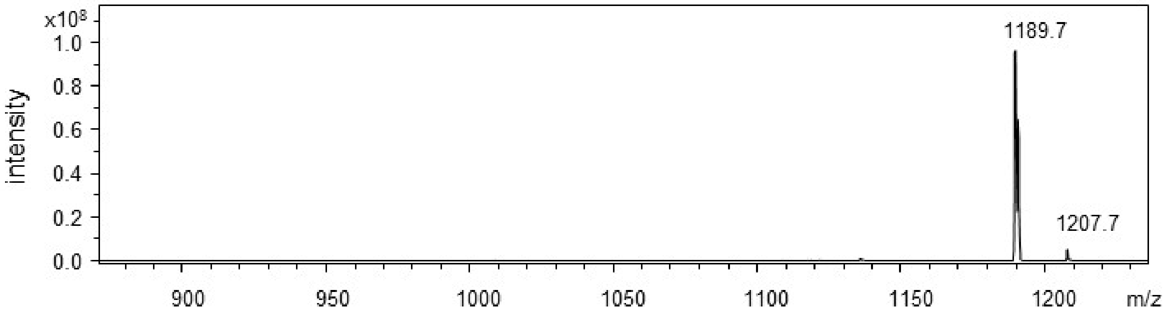


MS$^3$ (1189.7 → x)

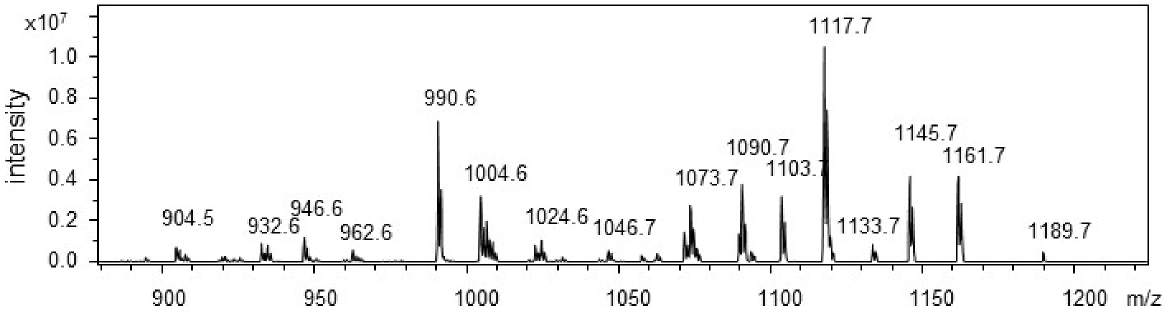


MS$^4$ (990.6 → x)

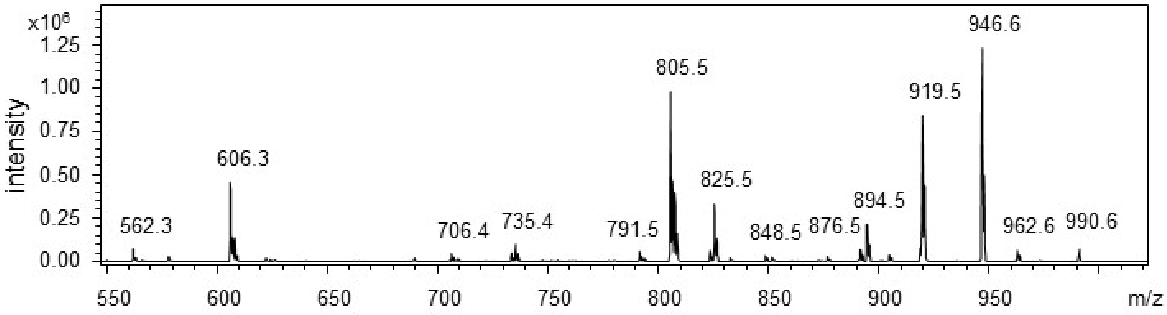

MS$^5$ (805.4 → x)

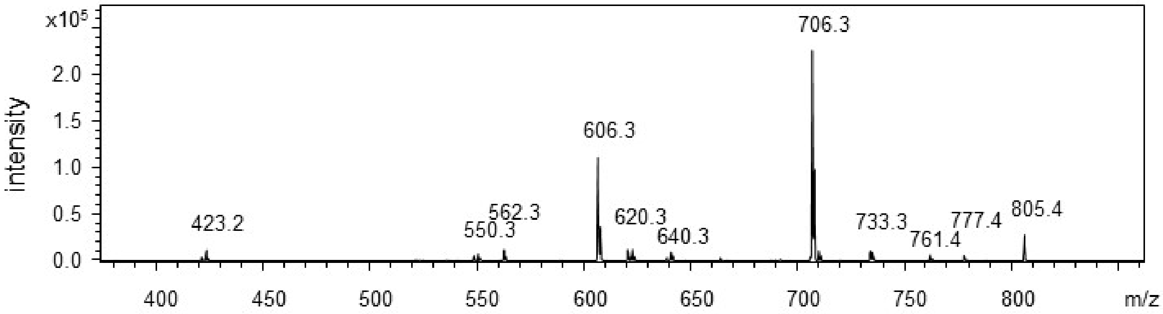


MS$^6$ (606.3 → x)

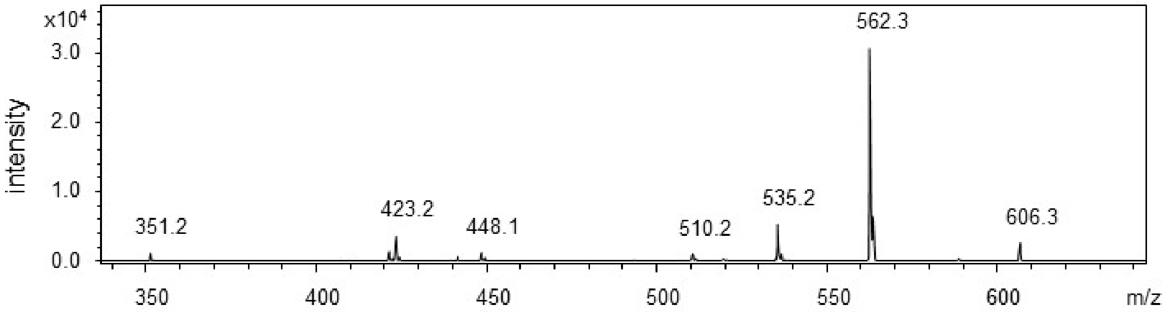


**Isocereulide M (7):**

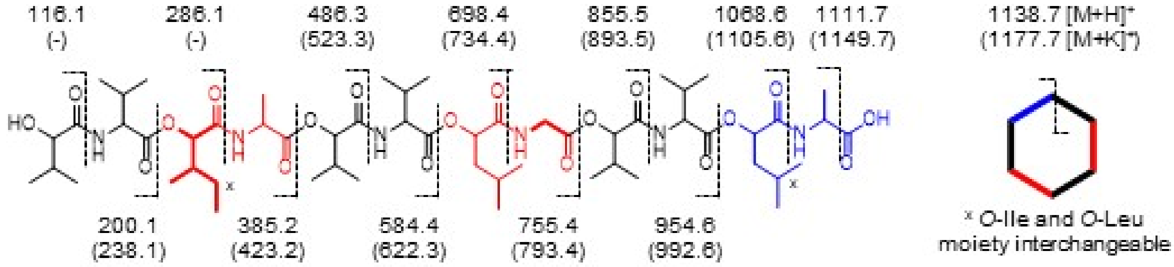

MS

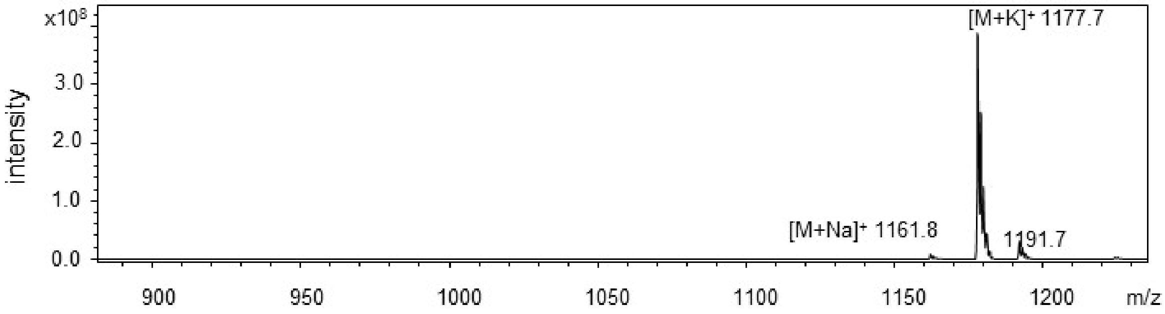


MS$^2$ (1177.7 → x)

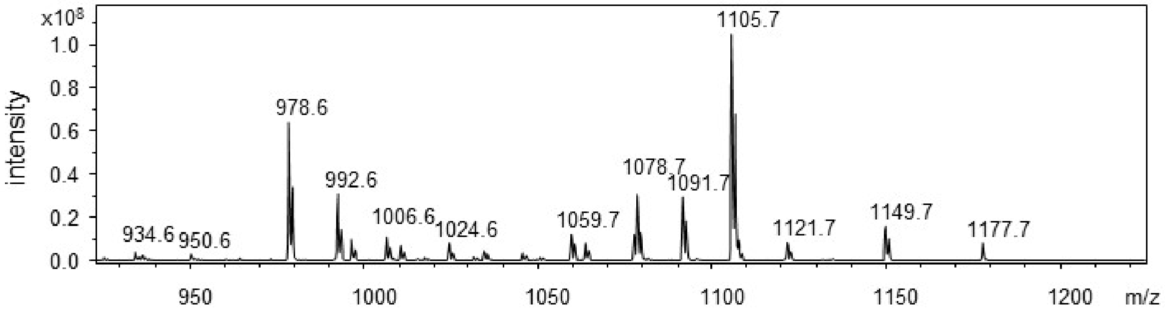


MS$^3$ (992.6 → x)

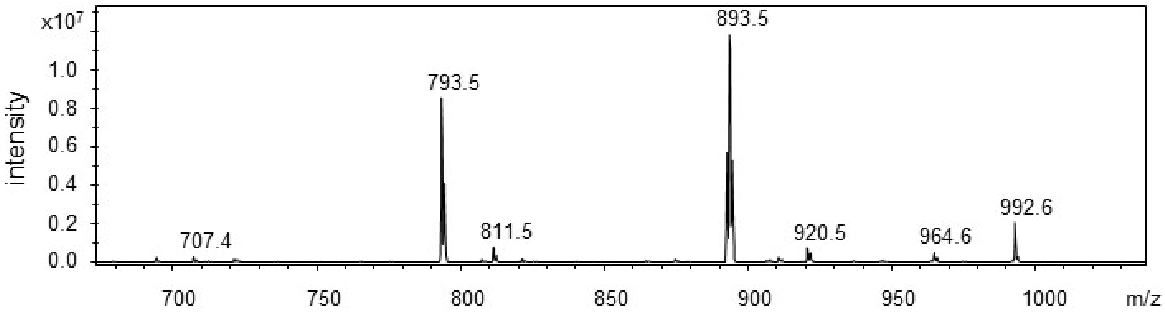


MS$^4$ (793.4 → x)

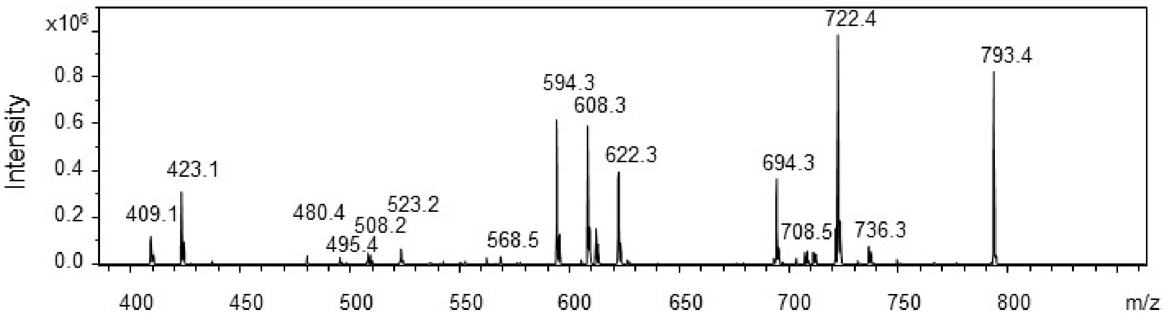

MS$^5$ (622.3 → x)

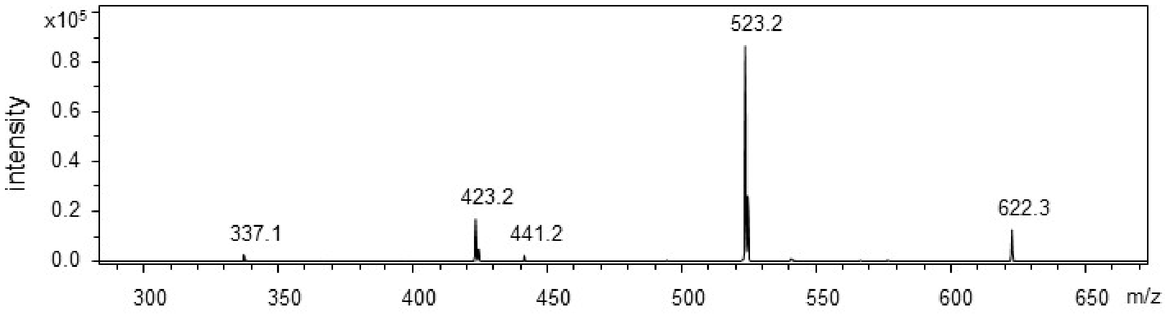


MS$^6$ (523.2 → x)

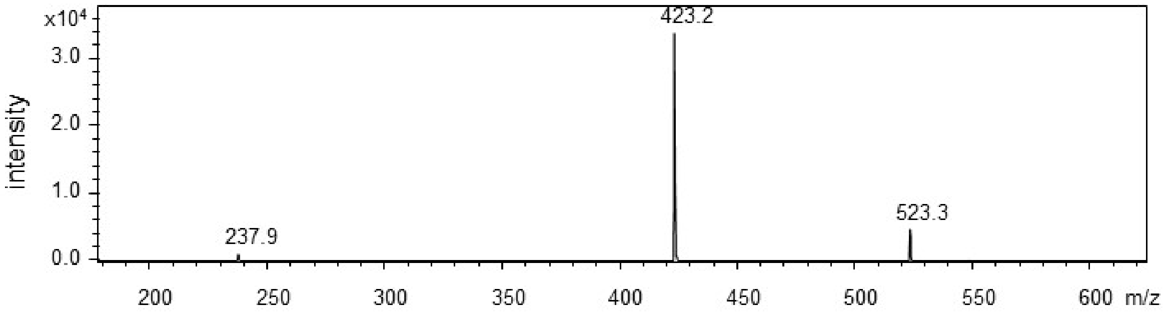

G. Toxin composition of samples used in HEp-2 cell assay and their $EC_{50}$ values

**Table S2.** Sample composition of purified cereulide (**1**) and isocereulides H–N (**2–8**) for the HEp-2 cell assay.

| Sample[a] | Percentile share of cereulide and isocereulides A–N in the tested samples (%)[b] | | | | | | | | | | | | | | $EC_{50}$ value[c] (ng/mL) |
|---|---|---|---|---|---|---|---|---|---|---|---|---|---|---|---|
| | Cer | iCerA | iCerB | iCerC | iCerD | iCerE | iCerF | iCerG | iCerH | iCerI | iCerJ | iCerK | iCerL/N | iCerM | |
| Cereulide (**1**) | 99.3 | 0.1 | 0.5 | – | – | 0.1 | – | – | – | – | – | – | – | – | 2.44 ± 0.27 |
| iCer H (**2**) | 8.7 | – | – | 0.2 | 0.1 | – | – | – | 88.1 | 1.4 | 1.5 | – | – | – | 6.62 ±1.93 |
| iCer I (**3**) | 7.6 | – | 0.4 | – | – | – | – | – | – | 60.0 | 32.0 | – | – | – | 1.75 ± 0.26 |
| iCer J (**4**) | 9.8 | – | 4.4 | – | – | 1.2 | – | 2.3 | – | 1.1 | 81.1 | 0.1 | – | – | 3.45 ± 0.44 |
| iCer K (**5**) | 0.5 | 3.0 | – | – | – | – | – | 3.3 | – | – | – | 93.2 | – | – | 3.56 ± 0.41 |
| iCer L/N (**6**+**8**) | – | – | – | 1.9 | – | – | – | – | – | – | – | – | 98.1 | – | 3.91 ± 0.80 |
| iCer M (**7**) | 6.8 | – | – | – | – | – | – | – | – | – | – | – | – | 93.2 | 4.43 ± 0.47 |

[a] Sample used to perform the HEp2-cell assay, named upon the major component. “Isocereulide” abbreviated as “iCer”.

[b] Percentile shares of cereulide and isocereulides A – N in the tested samples (isocereulide L and N are quantified together) with “–“ indicating no quantifiable amounts of the respective analyte; quantification performed *via* UPLC-MS/MS.

[c] $EC_{50}$ value determined *via* HEp2-cell assay in ng/mL

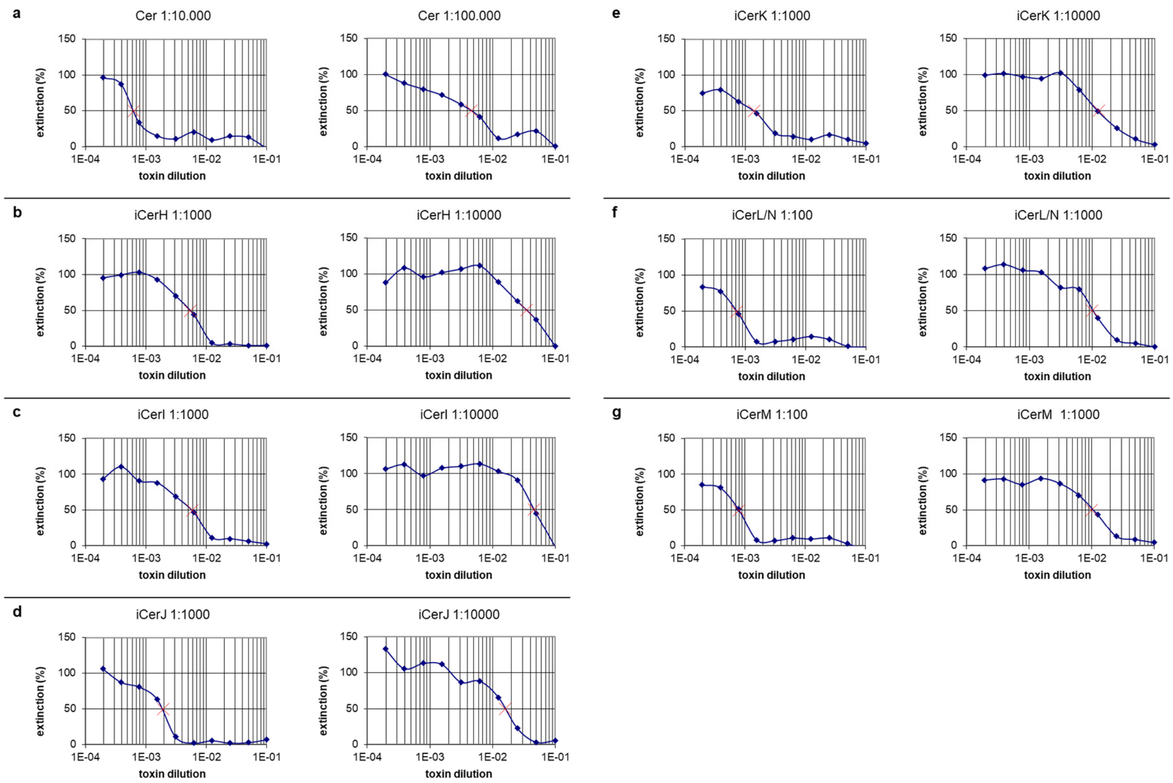


**Figure S30.** Representative raw data from HEp-2 cell culture assays of (**a**) cereulide in dilutions 1:10000 and 1:100000, (**b**) isocereulide H (**2**) in dilutions 1:1000 and 1:10000, and (**c**) isocereulide I (**3**) in dilutions 1:1000 and 1:10000, (**d**) isocereulide J (**4**) in dilutions 1:1000 and 1:10000 (**e**) isocereulide K (**5**) in dilutions 1:1000 and 1:10000, (**f**) isocereulide L/N (**6+8**) in dilutions 1:100 and 1:1000, and (**g**) isocereulide M (**7**) in dilutions 1:100 and 1:1000. Samples were diluted in EtOH, and each substance was tested in four biological repilca of the two different dilutions.

References

(1) Marxen, S.; Stark, T. D.; Frenzel, E.; Rütschle, A.; Lücking, G.; Pürstinger, G.; Pohl, E. E.; Scherer, S.; Ehling-Schulz, M.; Hofmann, T. *Anal. Bioanal. Chem.* **2015**, *407*, 2439–2453, DOI: 10.1007/s00216-015-8511-y.
(2) Walser, V.; Kranzler, M.; Ehling-Schulz, M.; Stark, T. D.; Hofmann, T. F. *Molecules* **2021**, *26*, 1360, DOI: 10.3390/molecules26051360.
(3) Bauer, T.; Stark, T.; Hofmann, T.; Ehling-Schulz, M. *J. Agric. Food Chem.* **2010**, *58*, 1420–1428, DOI: 10.1021/jf9033046.
(4) Ohmori, T.; Mutaguchi, Y.; Doi, K.; Ohshima, T. *J. Biosci. Bioeng.* **2012**, *114*, 457–459, DOI: 10.1016/j.jbiosc.2012.05.004.
(5) Chan, W. C.; White, P. D.; Editors. *Fmoc Solid Phase Peptide Synthesis: A Practical Approach*; Oxford Univ. Press, 2000.
